# Late Pleistocene glacial transitions in North America altered major river drainages, as revealed by deep-sea sediment

**DOI:** 10.1038/s41598-018-32268-7

**Published:** 2018-09-14

**Authors:** Andrea Fildani, Angela M. Hessler, Cody C. Mason, Matthew P. McKay, Daniel F. Stockli

**Affiliations:** 1 Statoil Research Center, Austin, TX 78730 USA; 2 The Deep Time Institute, Austin, TX 78755 USA; 3 Department of Geosciences, University of West Georgia, Corrollton, GA 30118 USA; 4 Department of Geography, Geology, and Planning, Missouri State University, Springfield, MO 65897 USA; 5 Jackson School of Geosciences, University of Texas at Austin, Austin, TX 78713 USA

## Abstract

Sediment eroded from continents during ice ages can be rapidly (<10^4^ years) transferred via rivers to the deep-sea and preserved in submarine fans, becoming a viable record of landscape evolution. We applied chemical weathering proxies and zircon geo-thermo-chronometry to late Pleistocene sediment recovered from the deep-sea Mississippi fan, revealing interactions between the Laurentide ice sheet (LIS) and broader Mississippi–Missouri catchment between ca. 70,000 and 10,000 years ago (70 to 10 ka). Sediment contribution from the Missouri catchment to the Mississippi fan was low between 70 and 30 ka but roughly doubled after the Last Glacial Maximum (LGM). Therefore, pre-LGM glacial advance profoundly altered the vast Missouri drainage through ice dams and/or re-routing of the river, thereby controlling the transfer of continental debris and freshwater toward southern outlets.

## Introduction

Often occurring over geologically short (<10^4^ years) time scales, large-scale paleo-geographic changes related to Pleistocene ice sheets significantly affected the evolution and migration patterns of flora and fauna, including early humans^1,2^. For instance, in North America the demise of the ‘bridge’ connecting the Laurentide and Cordilleran ice sheets may have allowed southward passage by early humans^3^, and the meltwater generated by the final collapse of this bridge ~14.5 ka caused significant sea level rise and global climate change^4^. However, geomorphologic evidence for earlier cycles of ice advance and retreat have been mostly obscured by erosion and deposition associated with the LGM (30 to 19 ka)^5,6^, particularly in North America^7^, where ice reached latitudes as low as 38°N^8,9^.

Where the terrestrial record of sediment produced by ice interacting with landmasses is lacking or, when present, highly discontinuous, we can look to far-flung sediment accumulations (e.g., deep-sea fans), which house thick accumulations of sediment eroded from and transported across continental-scale drainages^10^, and are thereby archives of climate and tectonic change occurring over different time frames^11,12^. Composition and maturity of sediment delivered to continental margins and eventually to the deep sea are affected by upstream boundary conditions — *e*.*g*., temperature, weathering regimes, bedrock type, glacial cover, hydrology^12,13^ — and therefore any signals of terrestrial ice sheet-sedimentary dynamics may be extracted by examining submarine sediment accumulations. Furthermore, on the North American continent, catastrophic glacial meltwater outbursts have facilitated the transport of sediment and any accompanying environmental signals across broad, stable catchment areas over glacial–interglacial time scales^12,14^.

## The Mississippi Source to Sink System

**Figure 1:**
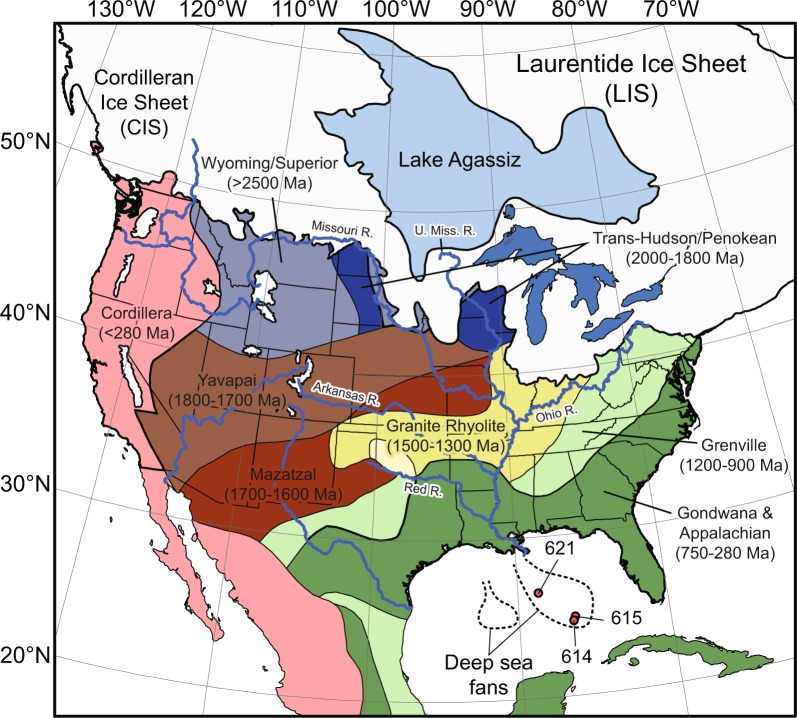
Gulf of Mexico deep-water fans (dashed lines) are fed by the Mississippi River drainage. Deep-water Sites from the Deep Sea Drilling Program are denoted. The spatial relationship between U-Pb zircon age domains (modified after^12^ and references herein) and the maximum Laurentide glacial ice extent^8^ demonstrate the correlation between high, >2.0 Ga zircons in Gulf of Mexico sediment and glacial outwash during late Wisconsin deglaciation. Headwaters of the Missouri, Mississippi, and Ohio Rivers are influenced by ice-sheets dynamics.

The Mississippi submarine fan, at the terminus of the late Pleistocene Mississippi River system (Fig. 1), holds more than 290,000 km^3^ of Pleistocene aged detritus, representing a sedimentary record of climatic fluctuations through multiple glacial–interglacial cycles (e.g., Marine Isotope Stages (MIS) 1–15. The Mississippi–Missouri drainage basin spans latitudinal gradients (~49°N to 29°N; Fig. 1) and was affected by high magnitude Pleistocene climate change that resulted in glacial diversion of rivers, meltwater mega-floods and the delivery of freshwater and sediment to the Gulf of Mexico (GOM) following the LGM <15 ka^12,15,16^. However, little direct evidence for the pre-LGM configuration and southern extent of the LIS—and its transformation of major river drainages—is preserved in North America. Here we integrate data from the deepest sediment core drilled by the Deep Sea Drilling Program (DSDP) Leg 96 on the Mississippi fan (Fig. 1) to reveal major modifications to the Mississippi–Missouri drainage by the southern LIS over the past ~70 ka. Growth of the LIS began over the eastern Canadian Arctic ~116 ka (MIS 5d), and experienced punctuated episodes of expansion (~65 ka: MIS 4) and retreat (~50–40 ka: MIS 3) until reaching its maximum extent during the LGM approximately 26–25 ka (MIS 2). Mountain glaciers were near or at their maximum extent by ~30 ka, which is broadly contemporaneous with the interval when global ice sheets first reached their maxima^5^.

Channel belts in the lower Mississippi valley indicate that the Mississippi River was meandering during the last interglacial (MIS 5b) as late as 85 ± 7 to 83 ± 7 ka, then switched to braided ~77 ± 4 ka as a result of increased discharge and/or sediment load due to glaciation in its headwaters^17^. Microfossil assemblages, oxygen and carbon isotopes curves obtained from slackwater lake deposits along the Mississippi Valley recorded cooler climates in the middle Mississippi valley after 55 ka interpreted as influenced by ice-sheet growth^18^. Ice lobes diverted the ancient Mississippi River (i.e., the Lake Michigan Lobe) and extensive ice movements reconstructed by tills and lacustrine deposits (i.e., Des Moines and James ice lobes)^18^ depict an active ice-sheet that exerted influence on the upper Mississippi and Ohio fluvial drainage patterns^19^ and on the Missouri River catchment, although the details of such interactions are still poorly documented.

The late Wisconsin Mississippi river-to-sea system was one of Earth’s largest sediment-routing networks during the most recent glacio-eustatic cycle^20^. Between the LGM and the Younger Dryas cold event (~12 kyr) it has been speculated that about 90% of North American meltwaters were discharged into the GOM via the Mississippi River drainage system^16^, making the GOM a key record of LIS decay, and making the southern U.S. the only continental margin containing evidence of meltwater outflows contemporaneous with global meltwater pulse mwp-1A^16^. Much of the sediment carried by these meltwaters ended up in the Bryant and Mississippi fans (Fig. 1).

**Figure 2:**
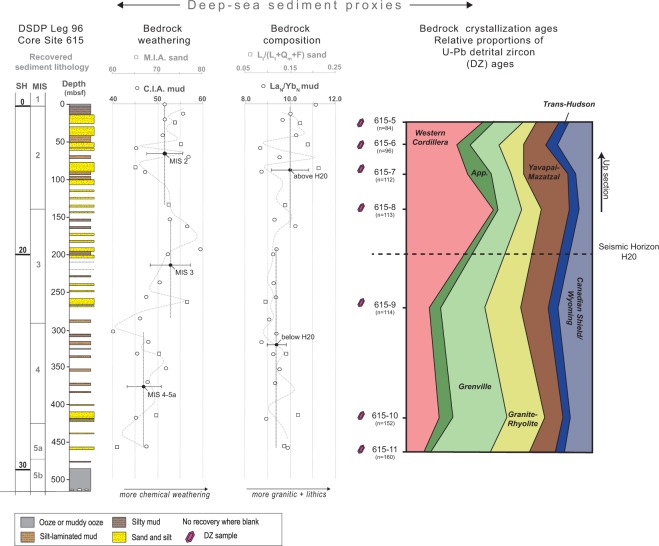
Stratigraphic column reconstructed with recovered core from Site 615 with corresponding sediment proxies for catchment bedrock weathering, composition, and age. Weathering proxies CIA (mud; Al_2_O_3_/[Al_2_O_3_ + CaO_silicate_ + Na_2_O + K_2_O])^25^ and MIA (sand; Q_m_/[Q_m_ + F]^26^ show that bedrock weathering increases with decreasing ice volume across H20; composition proxies La_N_/Yb_N_ (mud) and L_t_/Q_m_ + F + L_t_ (sand) show a change in overall provenance across H20. Q_m_ = monocrystalline quartz; F = total feldspar; L_t_ = total lithic grains; N = normalized to chondrite. Detrital zircon (DZ) U-Pb age components reflect provenance from North American terranes (color-coded) given as relative proportions (normalized to total number of zircon grains). Low proportion of Western Cordillera ages and higher proportion of Grenville ages are targeted evidence for diminished Missouri River influence below H20. SH = Seismic Horizon; MIS = Marine Isotope Stage.

In 1984, the DSDP Leg 96 drilled a series of boreholes into the Mississippi fan and Orca sub-basin, and a single site (615) recovered sediment deposited prior to the LGM, from MIS 5 (~70 ka) until recent. Seismic stratigraphy of the Mississippi fan defined eight prominent acoustic reflectors (or horizons) of regional extent identifying at least seven seismic-stratigraphic packages thought to correlate to climatic variation^21^. Ultimately, only the core at Site 615 penetrated the two shallowest seismic reflectors, H20 and H30 (Fig. 2), reaching interglacial deposits correlated to MIS 5^22^. H20 and H30 represent lithological contrasts in the stratigraphic packaging with more sand-prone deposits of the shallower interval (H0-20), and the mud-prone deposits of the deeper stratigraphic package (H20-30; Fig. 2). H20, the youngest mappable seismic horizon^21^, is interpreted to represent a regional erosion surface formed during an increase in sediment delivery to the Gulf of Mexico around the MIS 3–2 transition^23^. Sediment supply was estimated to be an order-of-magnitude higher during deposition of the stratigraphic package between the seafloor and H20, compared to the underlying interval H20-30; the relative volume of sediment deposited during accumulation of H0-20 was ∼17,500 km^3^ compared to only ∼3,500 km^3^ for H20-30^23^. The Mississippi River morphology and dynamics changed around 77 ± 4 ka because of forced modulation of discharge and sediment load^17^. Climate, varying through higher amplitude fluctuations during the Pleistocene than during the prior ~30 million years (e.g.^24^), would have had widespread effects on subaerial processes like chemical weathering, erosion, and the transport or sequestration of sediment in waterways. Much of the direct landscape response (e.g., soils, channelized drainages) to these processes has been eroded away. However, we know that some sedimentary signals (e.g., sand and clay composition) have been faithfully transferred downstream through large drainage basins and that sedimentary deposits preserve evidence of upstream climate change along continental margins^13^ and in deep-sea fans (e.g.^12^).

## Results

We targeted DSDP Site 615 to address inorganic geochemical, mineralogical, and geochronological variability of the clay and sand fractions covering MIS 5a to MIS 1. We sampled all available, non-carbonate lithologies from Site 615 (Fig. 2), from which sediment was somehow evenly (but not continuously; see gaps on Fig. 2) recovered in 47 cores to ~450 m below the seafloor. The inorganic composition of late Wisconsin sediment from the Mississippi fan was used as a proxy for two landscape parameters:^1^ the chemical weathering of bedrock and^2^ the original composition of bedrock (Fig. 2). The Chemical Index of Alteration (CIA) for clay and the Mineral Index of Alteration (MIA) for sand both quantify the loss of mobile elements and minerals relative to immobile elements and resistant minerals, respectively, during the *in situ* chemical weathering of bedrock (Data Repository)^25,26^. The degree of chemical weathering generally increases with higher temperatures and more rainfall^13,27^, such that a higher index points to a warmer, wetter climate. We see that in the Mississippi fan sediments, CIA and MIA fluctuate predictably with stadial-interstadial cycles; index values are relatively low during the colder MIS 4–5a and MIS 2 intervals and higher during the interstadial MIS 3 interval (Fig. 2). While this does not provide a new constraint on Pleistocene climate per se, the relationship confirms that Mississippi fan sediment is a reliable record of upstream landscape processes.

The signals for bedrock composition (L_t_/L_t_ + Q_m_ + F for sand; La_N_/Yb_N_ for mud; Fig. 2) change independently of the weathering indices, instead reflecting a shift in the drainage geography roughly contemporaneous with the H20 seismic horizon. Below H20, muds have a chondrite-normalized rare-earth element (REE) composition that was derived from overall less-fractionated bedrock compared to muds above H20. Sands above H20 contain more lithic rock fragments relative to quartz and feldspars, and the grains above H20 are relatively enriched in felsic volcanic fragments and polycrystalline quartz compared to carbonate and plutonic quartz (Supplementary Data in Repository). What the mud and sand composition implies, therefore, is that the Mississippi drainage adjusted abruptly between MIS 3 and MIS 2, such that more of its tributaries were eroding quartzose metamorphic and felsic volcanic bedrock and associated deposits.

Can we pinpoint which sector(s) of the Mississippi-Missouri drainage were affected by ice sheet dynamics just prior to the LGM? By analyzing the precise U-Pb crystallization and U-Th/He exhumation (i.e., cooling) ages of individual zircon crystals in Mississippi fan sands, we delineate major changes to the Missouri River catchment, which is today the largest contributor of sediment and freshwater to the Mississippi River system^28^. Previous studies involving U-Pb and U-Th/He age dating of detrital zircons in the youngest Mississippi fan strata revealed continental-scale sediment transfer to the deep Gulf of Mexico in response to millennial-scale (<10^4^ yrs) climate change where enhanced glacial erosion effectively expanded the Mississippi catchment north into the Superior terranes and west–southwest into the Western Cordillera volcanic terranes^12^ compared to the modern drainage. We contrast these data with new samples collected below seismic horizon H20 to see if the detrital thermo-chronological provenance signal would corroborate the results from mud geochemistry and sand petrography.

DZ age distributions and zircon U-Th/He cooling ages in sediment from the deep-sea Mississippi Fan show significant changes in age spectra across the H20 reflector, while DZ age spectra also exhibit a change across the LGM to Holocene transition (Fig. 2). Detrital zircon U-Pb age spectra for samples 615-5 through 8 (above H20) contain large proportions of the Western Cordillera age component (<280 Ma), varying proportions of grains from the mid-continent (Granite-Rhyolite Province), southwestern U.S. (Yavapai-Mazatzal) and Great Lakes/Canada (Superior) regions, and moderate proportions of grains of Grenville age, the most common DZ age component in the eastern U.S. and Canada. Below H20, samples contain much higher proportions of Grenville, Granite-Rhyolite, and Archean age components, and generally far fewer Western Cordillera grains (Fig. 2). These patterns support a scenario of a stark increase in sediment supply from terranes rich in Western Cordillera age zircons (e.g.^12,28^) across the MIS 3–2 transition. The contrast is clearly expressed using multidimensional scaling (MDS map; Data Repository) where samples above H20 are most similar to Missouri River samples, and samples below H20 show affinity to Upper Mississippi, Ohio, Red River, and Arkansas samples.

Mixing models allow us to more robustly quantify the relative contribution of distinct sediment source areas within the terrestrial Mississippi system to sedimentary deposits preserved in the Mississippi fan over the last ca. 70,000 years. We use a top-down unmixing approach to model relative contributions (Data Repository) of parent components — published DZ age distributions from the modern major tributaries to the Mississippi system^28^ — to daughter mixtures (composites), or DZ age distributions from deep-sea fan samples (see Data Repository). Mixing model results from samples measured across H20 indicate a significant change in sediment source areas within the Mississippi system. Unmixing the oldest DZ samples recovered below H20 (MIS 3 and older, or ca. >30–70 ka) results in contributions from the Missouri River component of only 15–36%. Remaining contributions from tributary components to composite fan mixtures are distributed between the Upper Mississippi River (26–40%), Ohio River (5–19%), and Red River components (10–31%). The Arkansas River component is present in only sample 615-11, where it contributes 17% to the mixture.

Samples above H20 (MIS 2; <30–10 ka) yield sediment mixing proportions with at least double those of mid-glacial contributions from the Missouri River which reaches up to 86% of sediment. The Upper Mississippi parent component supplies the next greatest proportion of sediment, or between 10–26%, the Ohio River component contributed only 1% of the sediment found in each sample above H20 (see Data Repository^28^).

**Figure 3:**
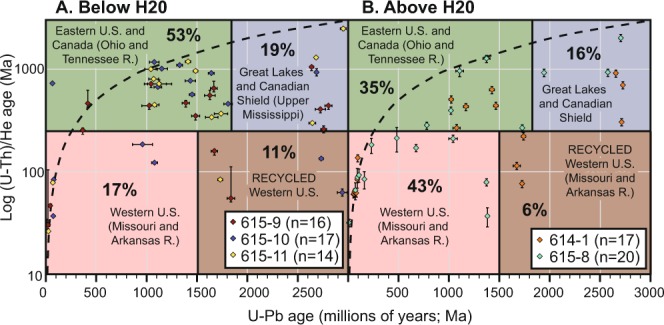
U-Th/He plots show differences in provenance (in terms of bedrock uplift and sediment yielding) above and below seismic horizon H20; it is noticeable the relative paucity of Western U.S.-derived grains and the Eastern U.S. and Canadian shield yielded grains below seismic reflector H20.

New detrital zircon (U-Th)/He ages for 47 U-Pb dated zircon grains from H20-H30 fall into distinct thermo-chronologic modes and, when compared to data from MIS2 (H0-H20 interval), the largest modes correspond to the exhumation ages assigned to Appalachia (Eastern U.S.A., and Eastern Canada) and Grenville, Granite-Rhyolite (or Mid-Continent), and Superior provinces. The distinctive younger U-Th)/He ages, related to Western Cordilleran magmatism and uplift after ~280 Ma, are sparse (Fig. 3) in contrast to the latest Wisconsin (H0-20) DZ U-Pb crystallization ages and (U-Th)/He cooling ages. We can conclude that the Missouri and Upper Mississippi catchments represent the only probable regions where erosion of the underlying bedrock would generate sand rich in zircon grains of both Western Cordillera and Superior ages^12,28^. These catchments correspond to regions that would have experienced glacially-enhanced sediment production during the LGM^29^, and later increased sediment transfer via meltwater megafloods during deglaciation^16,30^. Expansion of the Missouri–Upper Mississippi catchment via glacio-isostatic adjustment around the time of the LGM (e.g.^20^), together with deglaciation and glacial outburst megafloods, may have contributed to the high proportion of Missouri–Upper Mississippi sediment found in late Wisconsin submarine fan deposits^12^. Deglaciation and glacial outburst floods heavily modified the landscape and helped deliver coarser-grained sediment into the marine realm over geologically short (<10^4^ yr) time frames^12,14^. It has been postulated that these glacial outbursts triggered hyperpycnal flows delivering sediment into the deep-water geologically instantaneously (within days to months)^16,30^.

## Summary

**Figure 4:**
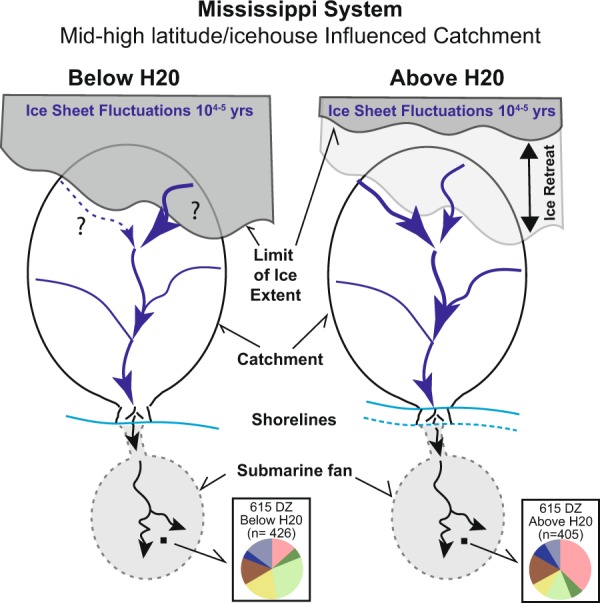
Simplified sketch diagram of the larger Mississippi System (Missouri-Mississippi River System to Mississippi deep-sea fan) highlighting ice-sheets fluctuation and overall ice modulation on the northernmost river catchments and the changes in provenance (DZ data) recorded in the deep-sea fan sediments (pie diagram proportions obtained from Fig. 2).

The Missouri River catchment contributed less than half of the detrital zircon yield to the Mississippi fan during MIS 4-5a (70–30 ka) than it did during late MIS 3 - MIS 2, when there was a major increase in sediment volume supplied to the GOM^23^ (Fig. 4). The relative paucity of sand below H20 could be interpreted as a reduction of coarse-grained sediment supply (and corresponding freshwater) to the marine realm during the MIS 4–5 glacial advance. We hypothesize that throughout MIS 4, the Missouri River was dammed by dynamic ice streams and lobes as extensive ice-sheets did influence the Missouri River catchment geomorphology^31^. Subsequent ice retreat and full connection of the Missouri catchment with the Mississippi River would have had strong effects on^1^ the landscape, and its migratory pathways for western North American biota and^2^ the atmosphere–hydrosphere system, through delivery and sequestration of continental debris and freshwater into the Gulf of Mexico, itself an influence on Atlantic Ocean circulation. Overall, the Mississippi deep-sea fan deposits reveal that glacial-interglacial transitions in North America did modify major river systems over the last 70 ka, that the modern geomorphological expression of the Missouri River is a product of the LGM, and that changes to the landscape were efficiently broadcasted to and preserved as sedimentary proxies in a submarine fan over geologically short (<10^4^ yr) time scales.

## Methods

We collected unconsolidated samples of sands and muds from DSDP Leg 96, Site 615, from below and above a regional seismic horizon (H20) within the Mississippi Fan (sampling depths: 615-5 ~25 m; 615-6 ~53 m; 615-7 ~85; 615-8 ~134.5 m; 615-9 ~264 m; 615-10 ~415.5; 615-11 ~457 m; see Fig. 2). A total of 21 mudstone samples and 8 samples of sands were collected to address inorganic geochemical, mineralogical, and geochronological variability of the clay and sand fractions. Sand mineralogy was determined by 300-grain petrographic point counts on a grid with 1 mm spacing, where thin sections were stained for potassium feldspar and plagioclase feldspar using sodium cobaltinitrite and barium chloride plus potassium rhodizonate, respectively. Mud samples were pulverized to 75 microns in Cr steel, then analyzed for major and trace elements by whole-sample ore grade x-ray fluorescence (XRF), and exploration grade inductively-coupled plasma mass spectrometry (ICP-MS; sodium peroxide fusion; graphite crucible) and inductively-coupled plasma atomic emission spectroscopy (ICP-AES; sodium peroxide fusion; graphite crucible) at SGS Mineral Analytical Services in Lakefield, Ontario (Canada).

We integrate six new Detrital Zircon (DZ) samples (~1–2 kg of sand), and one published sample^12^ from sediment cores recovered from the deep-sea Mississippi fan during DSDP Leg 96, Site 615^21^. All new DZ samples went through mineral separation and data were collected at the (U-Th) He and U-Pb Geo-Thermochronometry Laboratories at University of Texas, Austin, following the procedures herein stated (12; and https://www.jsg.utexas.edu/he-lab/); more details of the analytical methods are provided in the data repository.) These new data were integrated with published U-Pb DZ data from onshore samples of sand from each major tributary to the Mississippi: the Missouri, Upper Mississippi, Ohio, Arkansas, and Red River, and three amalgamated samples from the lower fluvio-deltaic Mississippi (Fig. 1)^27,32,33^.

A subset of zircon grains was selected for U-Pb crystallization and U-Th/He exhumation (i.e., double dating) ages of individual zircon crystals in Mississippi fan sands, to delineate major changes to the Missouri River catchment (see methods in 12; procedures at the UT Geo-Thermochronometry Laboratories).

## Sediment Unmixing Using Detrital Zircons

We use DZ geochronology to quantify relative contributions of sediment from Mississippi River tributaries to the deep-sea, using a top-down sediment unmixing approach^28,34–36^. The Mississippi catchment is well-suited to the application of DZ unmixing techniques because^1^: source terranes contain unique DZ age modes and proportions, and^2^ DZ populations in each major tributary to the Mississippi River, Delta, and Pleistocene deep-sea fan have been characterized^12,32,33^. We applied the Mason and colleagues^28^ approach to a new suite of sediment samples from DSDP leg 96, Site 615, collected below and above a regional seismic horizon (H20) in the Mississippi fan, representing deposition from the middle Wisconsin (ca. 30–80 ka, below H20) through the late Wisconsin glacial (ca. 10-30ka, above H20).

We followed published methods to model relative contributions of parent components—DZ age distributions from tributaries—to daughter mixtures (composites), or DZ age distributions from the deep-sea fan (after^28,34^). The probability density of DZ ages in a daughter mixture can be represented by a weighted sum of parent densities sampled at age *x*:

1 $$D(x)=\sum _{k=1}^{N}{\lambda }_{k}{P}_{k}(x),\,\sum _{k=1}^{N}{\lambda }_{k}=1$$

where *D*(*x*) is a model age density for the daughter composite constructed using KDEs (*P*_*k*_(*x*)) for the *N* composite samples defined as parent components in each model. We use an adaptive estimator that adjusts Gaussian kernel bandwidths based on local sample density to generate KDEs for each DZ sample^37,38^. We then use a Monte Carlo simulation to model 2e^6^ possible combinations of mixing weights (*λ*_*k*_), which sum to 100, and then select the model mixture *D(x)* with the lowest total variation distance from the daughter KDE:

2 $$\mathop{\min }\limits_{\lambda }(\frac{1}{2}\sum _{i=1}^{N}|D({x}_{i},\lambda )-{P}_{D}({x}_{i})|)$$

This mixture model assumes parent and daughter DZ samples accurately represent true source area DZ age distributions, and that daughter composites are pure mixtures of only the defined parent components. The model must also assume that KDEs used to model contributions are accurate representations of the population from which they were drawn. The results of our modeling are displayed in Fig. Supp. 1. We acknowledge that^1^ some Pleistocene sample composites have proportions of age modes that cannot be reproduced by modern tributary samples, and that^2^ increasing the number of measured grains in parent components (i.e.»100) would provide a more robust characterization of each sample. However, despite these constraints, estimates of relative sediment contributions to the modern fluvio-deltaic Mississippi based on DZ mixture models have excellent positive correlation to measured and historic relative suspended sediment loads measured in tributaries to the lower Mississippi system since ca. the 1900s^28^.

Above H20, samples 615-5 through 615-8 contain large proportions of the Western Cordillera mode (<280 Ma), varying proportions of Granite-Rhyolite, Yavapai-Mazatzal and Superior modes, and moderate proportions of the Grenville age mode. Below H20, samples contain much higher proportions of Grenville, Granite-Rhyolite, and Archean age modes, and generally far fewer Western Cordillera grains. A decrease in the Western Cordillera age mode is significant for samples 615-9, and 615-11, yet less pronounced in 615-10 (Fig. 2; and Data Repository). These patterns support a model of increasing sediment supply through time from source terranes rich in Western Cordillera age zircons (*e*.*g*.^12,28^).

## Electronic supplementary material

Dataset_ALL

## Data Availability

All data are available and accessible in Data Repository File.
