# Supplementary material for: Late Pleistocene glacial transitions in North America altered major river drainages, as revealed by deep-sea sediment
Source: Sci Rep. 2018 Sep 14;8:13839. doi: 10.1038/s41598-018-32268-7 (PMC6138750; doi:10.1038/s41598-018-32268-7)
Supplement: Supplementary file 1 — Dataset_ALL [file 41598_2018_32268_MOESM1_ESM.pdf]

Supplementary Information for Manuscript:

Late Pleistocene glacial transitions in North America altered major river drainages, as revealed by deep-sea sediment

Authors: Andrea Fildani, Angela M. Hessler, Cody C. Mason, Matthew P. McKay, and Daniel F. Stockli

Fildani et al. (2018) supplemental information and data

1. U-Pb zircon isotopic data
    615-5 1
    615-6 4
    615-7 7
    615-8 11
    615-9 15
    615-10 20
    615-11 25

2. Detrital zircon mixing models
    Methodology 30
    Results 31

3. (U-Th)/He zircon isotopic data 34

4. Sand framework analysis 38

5. Mud geochemistry 39

U-Pb isotopic data for sample 615-5

	[U] ppm	U/Th	$^{207}Pb/^{235}U$	2σ error	$^{206}Pb/^{238}U$	2σ error	RHO	$^{207}Pb/^{235}U$ Age Ma	2σ error	$^{206}Pb/^{238}U$ Age (Ma)	2σ error	$^{207}Pb/^{206}Pb$ Age (Ma)	2σ error	Best age (Ma)	2σ error	% Disc*	Rim or core
615_5B_1	435	1.38	0.03670	0.003	0.00522	0.00018	0.46	36.6	2.8	33.6	1.1	230	180	33.6	1.1	8.2	Rim
615_5B_1	149	1.60	4.27200	0.059	0.29370	0.00510	0.59	1688.0	11.0	1660.0	25.0	1723	24	1723.0	24.0	3.7	Core
615_5B_2	173	1.05	4.10800	0.042	0.27980	0.00310	0.67	1656.8	8.1	1590.0	16.0	1745	16	1745.0	16.0	8.9	
615_5B_3	397	2.12	0.07930	0.002	0.01168	0.00014	0.01	77.4	2.1	74.9	0.9	133	65	74.9	0.9	3.3	
615_5B_4	310	1.69	0.07630	0.002	0.01183	0.00016	0.03	74.6	2.1	75.8	1.0	81	72	75.8	1.0	1.6	
615_5B_5	176.8	1.36	0.08660	0.004	0.01238	0.00026	0.25	84.2	3.2	79.3	1.6	241	83	79.3	1.6	5.8	
615_5B_6	308	1.17	0.03500	0.002	0.00534	0.00018	0.22	34.9	2.0	34.3	1.1	120	150	34.3	1.1	1.7	
615_5B_7	129	1.71	4.21200	0.038	0.29630	0.00200	0.40	1675.8	7.4	1673.0	10.0	1670	16	1670.0	16.0	0.2	
615_5B_8	163.4	1.50	0.56800	0.014	0.07065	0.00078	0.12	456.1	8.9	440.1	4.7	585	60	440.1	4.7	3.5	
615_5B_9	166	1.05	0.09020	0.005	0.01302	0.00032	0.05	87.5	4.3	83.4	2.1	220	120	83.4	2.1	4.7	
615_5B_10	84.8	0.65	0.02930	0.004	0.00420	0.00024	0.11	29.2	3.8	27.0	1.5	270	260	27.0	1.5	7.5	
615_5B_11	258.7	1.04	0.09390	0.003	0.01401	0.00024	0.11	91.1	3.0	89.7	1.5	125	78	89.7	1.5	1.5	
615_5B_12	189.3	3.40	0.05750	0.003	0.00891	0.00019	0.15	56.7	2.6	57.2	1.2	90	100	57.2	1.2	0.9	
615_5B_13	97	2.20	1.86500	0.027	0.17980	0.00200	0.49	1068.4	9.7	1066.0	11.0	1072	27	1072.0	27.0	0.6	
615_5B_14	113.1	0.47	13.83000	0.100	0.54050	0.00430	0.58	2737.8	7.0	2787.0	18.0	2700	12	2700.0	12.0	3.2	
615_5B_15	409	4.54	0.19480	0.004	0.02855	0.00035	0.52	180.6	3.5	181.5	2.2	177	47	181.5	2.2	0.5	
615_5B_16	47.3	0.54	4.24000	0.110	0.28670	0.00510	0.46	1680.0	21.0	1625.0	26.0	1750	54	1750.0	54.0	7.1	
615_5B_17	1662	10.15	0.09850	0.002	0.01460	0.00013	0.02	95.3	1.7	93.4	0.8	142	42	93.4	0.8	2.0	
615_5B_18	94.4	1.49	0.24120	0.007	0.03505	0.00051	0.02	219.1	5.9	222.1	3.2	177	71	222.1	3.2	1.4	
615_5B_19	712	1.80	0.07280	0.002	0.01070	0.00015	0.28	71.3	1.5	68.6	0.9	161	49	68.6	0.9	3.7	
615_5B_20	504	1.72	0.07650	0.002	0.01146	0.00014	0.08	75.0	1.9	73.4	0.9	115	57	73.4	0.9	2.1	
615_5B_21	228	1.56	0.20150	0.010	0.02730	0.00050	0.51	186.1	7.9	173.6	3.2	317	95	173.6	3.2	6.7	
615_5B_22	138.7	1.09	0.65400	0.013	0.07787	0.00087	0.35	510.5	7.8	483.4	5.2	633	38	483.4	5.2	5.3	
615_5B_24	224.6	0.78	0.06190	0.002	0.00931	0.00021	0.12	60.9	2.3	59.7	1.4	134	87	59.7	1.4	2.0	
615_5B_25	58.5	0.66	12.15000	0.150	0.49430	0.00480	0.72	2617.0	12.0	2589.0	21.0	2636	14	2636.0	14.0	1.8	
615_5_1	60	1.03	3.89000	0.120	0.27740	0.00780	0.13	1606.0	26.0	1576.0	39.0	1665	28	1665.0	28.0	5.3	
615_5_2	81.6	1.64	12.74000	0.150	0.51120	0.00690	0.80	2660.0	11.0	2665.0	30.0	2668	15	2668.0	15.0	0.1	
615_5_4	42.5	0.54	5.14000	0.100	0.33730	0.00800	0.77	1841.0	17.0	1872.0	38.0	1811	26	1811.0	26.0	3.4	
615_5_10	241.8	1.85	4.69600	0.064	0.31120	0.00510	0.85	1766.0	11.0	1746.0	25.0	1796	15	1796.0	15.0	2.8	
615_5_11	45.1	1.22	1.85300	0.034	0.17760	0.00290	0.22	1063.0	12.0	1054.0	16.0	1087	36	1087.0	36.0	3.0	
615_5_12	317	0.83	0.03670	0.002	0.00556	0.00016	0.35	36.5	1.8	35.7	1.0	110	92	35.7	1.0	2.2	
615_5_14	123	2.50	0.06970	0.004	0.01119	0.00028	0.23	68.3	3.4	71.7	1.8	40	100	71.7	1.8	5.0	
615_5_20	322	0.50	1.84400	0.030	0.17700	0.00230	0.55	1060.0	11.0	1051.0	13.0	1101	28	1101.0	28.0	4.5	

	[U] ppm	U/Th	$^{207}Pb/^{235}U$	2σ error	$^{206}Pb/^{238}U$	2σ error	RHO	$^{207}Pb/^{235}U$ Age Ma	2σ error	$^{206}Pb/^{238}U$ Age (Ma)	2σ error	$^{207}Pb/^{206}Pb$ Age (Ma)	2σ error	Best age (Ma)	2σ error	% Disc*	Rim or core
615_5_21	388.1	1.83	0.03420	0.001	0.00525	0.00010	0.17	34.2	1.2	33.8	0.6	125	78	33.8	0.6	1.3	
615_5_22	217.6	1.64	0.20420	0.005	0.02938	0.00044	0.24	189.0	3.9	186.7	2.7	225	53	186.7	2.7	1.2	
615_5_25	61.5	0.85	2.52100	0.035	0.22160	0.00320	0.47	1279.0	10.0	1290.0	17.0	1252	27	1252.0	27.0	3.0	
615_5_26	315	2.65	0.19260	0.004	0.02833	0.00048	0.44	178.8	3.6	180.1	3.0	192	47	180.1	3.0	0.7	
615_5_27	431	1.30	0.18200	0.003	0.02726	0.00037	0.52	169.7	2.7	173.4	2.3	142	36	173.4	2.3	2.2	
615_5_28	283	1.57	0.10040	0.003	0.01468	0.00024	0.15	97.1	2.5	93.9	1.5	206	64	93.9	1.5	3.3	
615_5_32	42.7	1.11	2.37700	0.036	0.21840	0.00320	0.43	1237.0	11.0	1275.0	17.0	1184	32	1184.0	32.0	7.7	
615_5_34	156.8	1.16	2.05100	0.031	0.19040	0.00280	0.70	1132.0	10.0	1124.0	15.0	1151	26	1151.0	26.0	2.3	
615_5_35	194	1.17	0.18640	0.004	0.02759	0.00046	0.30	173.5	3.2	175.4	2.9	182	46	175.4	2.9	1.1	
615_5_36	35.3	0.35	14.25000	0.230	0.55100	0.00910	0.77	2765.0	15.0	2828.0	38.0	2723	18	2723.0	18.0	3.9	
615_5_37	109.6	1.19	11.94000	0.210	0.46800	0.00900	0.90	2600.0	17.0	2473.0	40.0	2696	13	2696.0	13.0	8.3	
615_5_39	100.8	1.05	5.03600	0.082	0.32650	0.00580	0.78	1825.0	14.0	1820.0	28.0	1829	22	1829.0	22.0	0.5	
615_5_40	64.5	1.83	2.10800	0.036	0.19260	0.00330	0.38	1152.0	12.0	1135.0	18.0	1172	32	1172.0	32.0	3.2	
615_5_42	41.7	0.58	4.81300	0.056	0.31950	0.00430	0.65	1786.3	9.9	1786.0	21.0	1759	20	1759.0	20.0	1.5	
615_5_43	142.8	1.93	0.02700	0.002	0.00400	0.00014	0.10	27.0	1.7	25.8	0.9	110	130	25.8	0.9	4.6	
615_5_44	121.8	1.96	2.96600	0.051	0.24130	0.00410	0.82	1398.0	13.0	1393.0	21.0	1380	25	1380.0	25.0	0.9	
615_5_45	111	1.58	0.21160	0.006	0.03072	0.00057	0.28	194.7	4.9	195.0	3.5	157	64	195.0	3.5	0.2	
615_5_47	130.3	1.79	4.44200	0.072	0.30470	0.00630	0.67	1719.0	13.0	1714.0	31.0	1712	28	1712.0	28.0	0.1	
615_5_48	5.82	1.13	26.70000	2.000	0.61100	0.02900	0.73	3374.0	79.0	3070.0	110.0	3556	86	3556.0	86.0	13.7	
615_5_49	95.4	1.69	3.24800	0.041	0.26200	0.00400	0.62	1467.9	9.8	1500.0	20.0	1428	23	1428.0	23.0	5.0	
615_5_53	22.5	6.41	7.99000	0.220	0.33180	0.00920	0.25	2229.0	25.0	1846.0	45.0	2634	56	2634.0	56.0	29.9	Rim
615_5_54	373	2.06	0.09130	0.003	0.01444	0.00024	0.33	88.6	2.6	92.4	1.5	37	59	92.4	1.5	4.3	
615_5_55	16.34	27.60	7.81000	0.180	0.33550	0.00560	0.47	2207.0	20.0	1864.0	27.0	2519	35	2519.0	35.0	26.0	
615_5_58	92.7	1.40	2.02200	0.035	0.19140	0.00380	0.72	1122.0	12.0	1129.0	21.0	1119	23	1119.0	23.0	0.9	
615_5_59	35	1.92	1.69000	0.034	0.16690	0.00280	0.31	1005.0	12.0	997.0	15.0	1075	37	1075.0	37.0	7.3	
615_5_61	35.4	0.78	2.89400	0.047	0.23410	0.00380	0.49	1381.0	12.0	1358.0	20.0	1407	33	1407.0	33.0	3.5	
615_5_63	114	2.13	1.70900	0.030	0.17040	0.00320	0.67	1011.0	11.0	1014.0	18.0	1019	28	1019.0	28.0	0.5	
615_5_64	144.6	1.58	1.68900	0.024	0.16750	0.00260	0.67	1003.6	9.1	998.0	15.0	1013	24	1013.0	24.0	1.5	
615_5_65	147.3	1.65	0.02940	0.003	0.00436	0.00018	0.06	29.4	3.4	28.0	1.2	210	220	28.0	1.2	4.8	
615_5_66	432.6	13.80	1.63900	0.029	0.15930	0.00250	0.85	986.0	11.0	953.0	14.0	1077	18	1077.0	18.0	11.5	
615_5_67	53.4	0.35	5.71000	0.100	0.34500	0.00600	0.69	1933.0	15.0	1914.0	28.0	1945	24	1945.0	24.0	1.6	
615_5_68	809	0.46	0.04600	0.001	0.00704	0.00010	0.23	45.8	1.0	45.2	0.7	90	50	45.2	0.7	1.3	
615_5_74	419	17.79	1.87000	0.024	0.18590	0.00260	0.71	1070.4	8.6	1099.0	14.0	1058	26	1058.0	26.0	3.9	Rim
615_5_74	197.6	1.37	3.03200	0.066	0.24010	0.00540	0.83	1415.0	17.0	1387.0	28.0	1444	21	1444.0	21.0	3.9	Core
615_5_80	30.5	1.57	6.85200	0.065	0.31340	0.00430	0.46	2091.9	8.4	1757.0	21.0	2422	23	2422.0	23.0	27.5	

	[U] ppm	U/Th	$^{207}Pb/^{235}U$	2σ error	$^{206}Pb/^{238}U$	2σ error	RHO	$^{207}Pb/^{235}U$ Age Ma	2σ error	$^{206}Pb/^{238}U$ Age (Ma)	2σ error	$^{207}Pb/^{206}Pb$ Age (Ma)	2σ error	Best age (Ma)	2σ error	% Disc*	Rim or core
615_5_92	75.1	0.64	0.58200	0.014	0.07420	0.00130	0.37	465.9	8.8	461.3	7.5	447	54	461.3	7.5	1.0	
615_5_94	61.2	0.92	1.93400	0.030	0.18820	0.00260	0.59	1092.0	10.0	1111.0	14.0	1066	27	1066.0	27.0	4.2	
615_5_95	76.2	0.77	14.0800	0.160	0.53440	0.00710	0.79	2755.0	11.0	2759.0	30.0	2753	14	2753.0	14.0	0.2	
615_5_96	85.7	1.26	2.80200	0.050	0.23510	0.00390	0.67	1355.0	13.0	1361.0	20.0	1318	22	1318.0	22.0	3.3	
615_5_97	242	1.94	0.07000	0.002	0.01060	0.00022	0.08	68.7	2.0	68.0	1.4	62	71	68.0	1.4	1.0	
615_5_98	191.8	2.61	0.06950	0.009	0.01060	0.00033	0.71	68.0	8.5	68.0	2.1	0	110	68.0	2.1	0.0	
615_5_99	860	2.01	0.03420	0.001	0.00534	0.00010	0.22	34.2	1.1	34.3	0.7	23	72	34.3	0.7	0.4	Rim
615_5_100	112	1.22	0.08100	0.004	0.01254	0.00026	0.15	79.0	3.3	80.3	1.6	48	81	80.3	1.6	1.6	
615_5_102	155.8	0.93	4.02400	0.099	0.28940	0.00750	0.86	1636.0	20.0	1637.0	37.0	1624	26	1624.0	26.0	0.8	
615_5_111	55.7	1.68	0.67900	0.025	0.07690	0.00370	0.72	525.0	15.0	481.0	21.0	761	70	481.0	21.0	8.4	
615_5_112	39.1	0.99	1.95400	0.038	0.18770	0.00290	0.49	1098.0	13.0	1109.0	16.0	1085	37	1085.0	37.0	2.2	
615_5_113	33.2	0.53	12.61000	0.170	0.49590	0.00770	0.67	2652.0	12.0	2595.0	33.0	2665	20	2665.0	20.0	2.6	
615_5_114	20.6	11.90	4.15000	0.120	0.24920	0.00480	0.38	1661.0	23.0	1434.0	25.0	1953	47	1953.0	47.0	26.6	
615_5_118	19.4	1.07	4.28500	0.066	0.30040	0.00450	0.53	1693.0	12.0	1692.0	22.0	1692	28	1692.0	28.0	0.0	
615_5_119	126.5	1.85	0.08220	0.004	0.01270	0.00028	0.17	80.1	3.3	81.3	1.8	77	85	81.3	1.8	1.5	
615_5_120	407	1.32	0.07870	0.002	0.01219	0.00020	0.17	76.9	2.0	78.1	1.3	50	55	78.1	1.3	1.6	

U-Pb isotopic data for sample 615-6

	[U] ppm	U/Th	$^{207}Pb/^{235}U$	2σ error	$^{206}Pb/^{238}U$	2σ error	RHO	$^{207}Pb/^{235}U$ Age Ma	2σ error	$^{206}Pb/^{238}U$ Age (Ma)	2σ error	$^{207}Pb/^{206}Pb$ Age (Ma)	2σ error	Best age (Ma)	2σ error	% Disc*	Rim or core
615_6_1	29.5	0.44	4.797	0.09	0.3206	0.0061	0.68	1782	15	1791	30	1783	24	1783.0	24.0	0.4	
615_6_2	212.0	1.09	1.849	0.03	0.1801	0.0040	0.81	1062	12	1067	22	1043	27	1043.0	27.0	2.3	
615_6_3	527.0	1.91	0.063	0.00	0.0095	0.0002	0.32	62	1	61	1	86	45	60.9	1.2	1.3	
615_6_5	28.5	1.37	2.185	0.04	0.1985	0.0027	0.19	1177	12	1167	14	1207	41	1207.0	41.0	3.3	
615_6_6	62.4	1.16	0.091	0.01	0.0133	0.0005	0.30	88	8	85	3	250	180	85.2	2.9	3.3	
615_6_9	79.8	0.44	9.720	0.23	0.4270	0.0130	0.89	2407	22	2289	57	2522	23	2522.0	23.0	9.2	
615_6_10	31.6	2.52	2.197	0.04	0.2007	0.0034	0.40	1180	13	1179	18	1168	39	1168.0	39.0	0.9	
615_6_12	118.0	1.32	2.403	0.03	0.2100	0.0028	0.61	1244	8	1229	15	1284	21	1284.0	21.0	4.3	
615_6_14	66.9	1.80	2.538	0.04	0.2151	0.0035	0.62	1286	11	1255	19	1329	27	1329.0	27.0	5.6	
615_6_15	399.0	3.18	12.180	0.15	0.4932	0.0077	0.83	2619	12	2583	33	2653	15	2653.0	15.0	2.6	
615_6_17	107.6	1.83	6.310	0.10	0.3646	0.0071	0.82	2023	15	2003	34	2040	19	2040.0	19.0	1.8	
615_6_18	27.7	0.92	6.920	0.11	0.3743	0.0052	0.53	2101	14	2049	24	2151	26	2151.0	26.0	4.7	
615_6_19	148.5	1.38	4.134	0.05	0.2689	0.0036	0.81	1660	9	1535	18	1830	14	1830.0	14.0	16.1	
615_6_21	99.8	1.14	3.750	0.06	0.2738	0.0052	0.62	1581	13	1560	27	1627	27	1627.0	27.0	4.1	
615_6_23	75.0	2.78	1.839	0.03	0.1724	0.0034	0.50	1058	11	1025	18	1123	36	1123.0	36.0	8.7	
615_6_25	88.0	1.80	2.067	0.04	0.1930	0.0039	0.66	1137	12	1137	21	1146	26	1146.0	26.0	0.8	
615_6_27	49.7	1.24	2.425	0.04	0.2042	0.0031	0.39	1249	11	1197	17	1340	31	1340.0	31.0	10.7	
615_6_29	72.3	1.29	15.510	0.20	0.5337	0.0088	0.77	2846	12	2764	38	2914	16	2914.0	16.0	5.1	
615_6_30	59.0	0.75	2.371	0.04	0.2085	0.0035	0.57	1236	11	1220	19	1281	29	1281.0	29.0	4.8	
615_6_31	120.1	2.41	1.937	0.03	0.1841	0.0036	0.74	1093	12	1089	19	1103	26	1103.0	26.0	1.3	
615_6_32	141.8	1.87	11.290	0.15	0.4748	0.0074	0.82	2546	12	2507	33	2581	15	2581.0	15.0	2.9	
615_6_33	104.9	1.73	1.846	0.04	0.1704	0.0026	0.42	1060	15	1014	14	1167	41	1167.0	41.0	13.1	
615_6_34	89.1	1.32	13.700	0.15	0.5370	0.0075	0.83	2728	10	2770	31	2699	13	2699.0	13.0	2.6	
615_6_35	113.4	0.81	0.098	0.00	0.0134	0.0003	0.28	95	3	86	2	340	66	85.9	2.1	9.3	
615_6_37	55.1	1.31	13.960	0.21	0.5327	0.0092	0.73	2746	14	2752	39	2740	20	2740.0	20.0	0.4	
615_6_38	27.2	0.77	1.539	0.04	0.1588	0.0034	0.33	946	14	952	19	946	53	946.0	53.0	0.6	
615_6_39	33.7	0.81	12.670	0.18	0.4975	0.0086	0.69	2656	13	2601	37	2693	20	2693.0	20.0	3.4	
615_6_42	43.4	1.91	1.721	0.03	0.1692	0.0026	0.46	1017	12	1007	14	1051	31	1051.0	31.0	4.2	
615_6_43	224.2	1.44	4.217	0.05	0.2898	0.0044	0.81	1678	10	1640	22	1717	17	1717.0	17.0	4.5	
615_6_46	430.0	1.78	0.101	0.00	0.0150	0.0003	0.45	98	2	96	2	163	52	96.0	1.9	1.7	
615_6_47	425.0	1.44	12.470	0.20	0.4946	0.0085	0.80	2641	15	2589	37	2670	17	2670.0	17.0	3.0	
615_6_48	150.0	1.84	5.880	0.11	0.3518	0.0080	0.85	1956	16	1941	38	1980	20	1980.0	20.0	2.0	
615_6_49	247.0	1.60	1.736	0.02	0.1716	0.0022	0.65	1021	7	1021	12	1037	21	1037.0	21.0	1.5	

	[U] ppm	U/Th	$^{207}Pb/^{235}U$	2σ error	$^{206}Pb/^{238}U$	2σ error	RHO	$^{207}Pb/^{235}U$ Age Ma	2σ error	$^{206}Pb/^{238}U$ Age (Ma)	2σ error	$^{207}Pb/^{206}Pb$ Age (Ma)	2σ error	Best age (Ma)	2σ error	% Disc*	Rim or core
615_6_50	3952.0	9.26	4.161	0.08	0.2863	0.0068	0.85	1666	16	1623	34	1735	21	1735.0	21.0	6.5	
615_6_51	193.0	1.08	1.864	0.03	0.1800	0.0027	0.72	1069	9	1067	15	1056	22	1056.0	22.0	1.0	
615_6_53	227.0	1.30	5.408	0.08	0.3400	0.0057	0.79	1885	13	1886	27	1895	18	1895.0	18.0	0.5	
615_6_54	70.6	0.70	3.908	0.08	0.2829	0.0050	0.71	1618	16	1605	25	1638	25	1638.0	25.0	2.0	
615_6_55	155.7	1.02	0.106	0.00	0.0157	0.0004	0.35	102	3	101	3	142	71	100.5	2.5	1.5	
615_6_58	134.4	1.72	3.206	0.04	0.2560	0.0030	0.68	1459	10	1469	15	1441	19	1441.0	19.0	1.9	
615_6_59	113.8	0.74	6.290	0.10	0.3610	0.0059	0.83	2017	14	1986	28	2048	14	2048.0	14.0	3.0	
615_6_60	128.0	1.63	0.538	0.01	0.0700	0.0011	0.52	437	6	436	6	425	36	436.1	6.4	0.2	
615_6_62	573.0	1.23	0.075	0.00	0.0116	0.0003	0.39	73	2	74	2	36	42	74.4	1.6	1.6	
615_6_63	149.0	0.76	4.226	0.06	0.2991	0.0052	0.80	1678	12	1686	26	1660	20	1660.0	20.0	1.6	
615_6_64	360.0	4.31	1.923	0.04	0.1826	0.0030	0.82	1088	13	1081	16	1106	20	1106.0	20.0	2.3	
615_6_66	38.8	0.58	4.229	0.08	0.3010	0.0062	0.66	1677	16	1695	31	1668	29	1668.0	29.0	1.6	
615_6_67	88.4	1.25	6.245	0.08	0.3661	0.0052	0.76	2010	10	2010	25	2012	15	2012.0	15.0	0.1	
615_6_68	109.0	0.74	3.018	0.04	0.2417	0.0029	0.60	1411	9	1399	16	1425	20	1425.0	20.0	1.8	
615_6_71	135.0	1.59	2.299	0.04	0.2051	0.0049	0.75	1211	13	1206	27	1217	27	1217.0	27.0	0.9	
615_6_72	288.0	1.38	0.422	0.01	0.0569	0.0009	0.61	358	5	357	5	369	29	356.7	5.3	0.2	
615_6_73	141.6	1.22	3.118	0.04	0.2499	0.0034	0.75	1437	10	1438	18	1432	18	1432.0	18.0	0.4	
615_6_74	191.0	1.20	0.103	0.00	0.0153	0.0003	0.08	100	3	98	2	131	73	97.8	2.1	1.7	
615_6_76	161.2	0.46	0.525	0.01	0.0689	0.0011	0.53	428	6	429	7	455	34	429.3	6.7	0.3	
615_6_77	132.1	0.64	14.010	0.19	0.5232	0.0088	0.82	2750	13	2711	37	2790	16	2790.0	16.0	2.8	
615_6_78	118.7	2.26	2.024	0.03	0.1898	0.0027	0.71	1124	10	1120	14	1138	21	1138.0	21.0	1.6	
615_6_79	493.2	2.64	0.084	0.00	0.0124	0.0002	0.39	82	2	79	1	135	49	79.1	1.3	3.5	
615_6_80	499.0	39.50	4.661	0.06	0.2997	0.0037	0.88	1760	10	1694	20	1845	13	1845.0	13.0	8.2	
615_6_81	71.4	1.41	2.567	0.06	0.2199	0.0046	0.53	1290	17	1286	23	1322	37	1322.0	37.0	2.7	
615_6_82	102.7	0.66	13.030	0.18	0.5138	0.0092	0.80	2683	14	2672	39	2707	18	2707.0	18.0	1.3	
615_6_84	153.8	4.11	4.228	0.09	0.2908	0.0059	0.96	1682	16	1645	29	1735	18	1735.0	18.0	5.2	
615_6_89	80.0	1.60	4.520	0.12	0.2880	0.0100	0.77	1733	22	1630	50	1869	26	1869.0	26.0	12.8	
615_6_90	65.5	0.65	3.090	0.04	0.2483	0.0039	0.55	1434	11	1432	20	1429	28	1429.0	28.0	0.2	
615_6_91	133.2	1.09	3.149	0.04	0.2497	0.0031	0.60	1444	9	1437	16	1464	20	1464.0	20.0	1.8	
615_6_99	122.8	1.87	3.173	0.04	0.2548	0.0042	0.71	1450	10	1463	22	1442	23	1442.0	23.0	1.5	
615_6_100	152.7	0.82	4.381	0.05	0.3000	0.0039	0.74	1708	9	1691	19	1738	17	1738.0	17.0	2.7	
615_6_101	275.0	4.47	4.770	0.06	0.3171	0.0046	0.85	1781	11	1775	23	1782	15	1782.0	15.0	0.4	
615_6_102	147.7	1.24	2.708	0.06	0.2053	0.0043	0.75	1330	17	1204	23	1529	28	1529.0	28.0	21.3	
615_6_104	297.6	1.90	2.261	0.03	0.2045	0.0027	0.67	1200	9	1199	14	1189	23	1189.0	23.0	0.8	
615_6_105	456.0	0.99	0.205	0.00	0.0294	0.0006	0.58	189	3	187	4	209	40	187.0	3.8	1.2	

	[U] ppm	U/Th	$^{207}Pb/^{235}U$	2σ error	$^{206}Pb/^{238}U$	2σ error	RHO	$^{207}Pb/^{235}U$ Age Ma	2σ error	$^{206}Pb/^{238}U$ Age (Ma)	2σ error	$^{207}Pb/^{206}Pb$ Age (Ma)	2σ error	Best age (Ma)	2σ error	% Disc*	Rim or core
615_6_107	409.0	1.44	0.068	0.00	0.0105	0.0002	0.25	66	2	67	1	60	52	67.4	1.2	1.5	
615_6_108	55.3	0.58	0.873	0.02	0.1025	0.0021	0.30	639	9	629	12	663	50	629.0	12.0	1.5	
615_6_109	273.9	1.94	12.690	0.21	0.4940	0.0086	0.90	2658	15	2592	38	2714	14	2714.0	14.0	4.5	
615_6_113	146.1	2.22	0.218	0.01	0.0311	0.0006	0.32	200	5	198	4	204	56	197.6	3.5	1.2	
615_6_114	163.0	2.00	5.994	0.08	0.3469	0.0059	0.81	1974	12	1919	28	2023	18	2023.0	18.0	5.1	
615_6_117	284.0	4.72	0.510	0.01	0.0669	0.0012	0.15	418	6	417	8	417	32	417.1	7.5	0.2	
615_6_120	142.3	2.72	16.120	0.19	0.5541	0.0081	0.87	2884	12	2840	34	2917	11	2917.0	11.0	2.6	
615-6-B1	168.0	2.04	0.078	0.00	0.0117	0.0002	0.18	77	3	75	2	115	67	75.1	1.5	2.3	
615-6-B11	143.5	1.91	0.214	0.01	0.0302	0.0004	0.23	197	5	192	2	267	58	191.9	2.2	2.6	
615-6-B12	95.4	2.53	0.103	0.00	0.0144	0.0004	0.08	100	5	92	2	230	100	92.2	2.2	7.5	
615-6-B14	81.8	1.22	0.079	0.01	0.0112	0.0003	0.03	77	5	72	2	240	140	71.5	1.9	7.5	
615-6-B19	117.9	1.46	2.897	0.04	0.2370	0.0027	0.55	1380	10	1373	14	1395	22	1395.0	22.0	1.6	
615-6-B2	192.1	1.04	0.107	0.00	0.0161	0.0003	0.11	103	3	103	2	116	65	102.6	1.8	0.8	
615-6-B20	65.3	1.21	0.094	0.01	0.0132	0.0004	0.16	91	5	85	3	240	120	84.7	2.5	6.8	
615-6-B21	497.0	1.42	0.108	0.00	0.0151	0.0003	0.31	104	2	96	2	278	55	96.3	1.7	7.7	
615-6-B22	21.2	0.50	5.219	0.08	0.3276	0.0049	0.17	1855	13	1826	24	1877	35	1877.0	35.0	2.7	
615-6-B23	142.1	1.45	0.203	0.01	0.0293	0.0005	0.19	187	5	186	3	195	61	186.2	3.1	0.5	
615-6-B27	511.0	2.43	0.062	0.00	0.0088	0.0002	0.04	61	2	57	1	235	74	56.7	1.1	6.4	
615-6-B29	112.3	1.36	0.571	0.04	0.0661	0.0012	0.58	456	23	413	7	670	120	412.7	7.2	9.5	
615-6-B30	135.1	1.86	0.104	0.00	0.0149	0.0003	0.32	101	4	95	2	217	92	95.4	2.1	5.2	
615-6-B32	87.5	1.53	6.795	0.08	0.3844	0.0040	0.58	2085	10	2097	18	2068	21	2068.0	21.0	1.4	
615-6-B33	270.3	2.14	0.100	0.00	0.0146	0.0003	0.09	97	2	93	2	180	59	93.4	1.6	3.6	
615-6-B34	211.7	2.96	0.211	0.01	0.0302	0.0005	0.27	194	5	192	3	230	66	192.0	2.8	1.1	
615-6-B35	146.4	1.01	0.201	0.01	0.0264	0.0006	0.02	186	6	168	4	416	88	168.1	3.6	9.7	
615-6-B36	175.0	1.84	0.074	0.00	0.0114	0.0003	0.13	72	4	73	2	0	110	73.0	1.9	1.5	
615-6-B5	111.2	0.61	3.341	0.05	0.2293	0.0028	0.47	1490	13	1333	14	1713	27	1713.0	27.0	22.2	
615-6-B6	81.1	0.73	0.415	0.01	0.0520	0.0008	0.02	352	9	327	5	480	76	327.2	5.0	6.9	
615-6-B9	434.0	1.55	0.106	0.00	0.0156	0.0002	0.10	102	2	100	1	163	60	99.5	1.2	2.5	

U-Pb isotopic data for sample 615-7

	[U] ppm	U/Th	$^{207}Pb/^{235}U$	2σ error	$^{206}Pb/^{238}U$	2σ error	RHO	$^{207}Pb/^{235}U$ Age Ma	2σ error	$^{206}Pb/^{238}U$ Age (Ma)	2σ error	$^{207}Pb/^{206}Pb$ Age (Ma)	2σ error	Best age (Ma)	2σ error	% Disc*	Rim or core
615_7_1	332.0	0.97	0.173	0.00	0.0254	0.0004	0.23	162	3	162	2	175	46	161.5	2.3	0.2	
615_7_3	595.0	1.05	0.523	0.01	0.0683	0.0015	0.80	427	6	426	9	440	31	426.0	8.9	0.1	
615_7_4	97.7	1.17	13.740	0.15	0.5279	0.0065	0.79	2731	10	2732	27	2725	11	2725.0	11.0	0.3	
615_7_5	77.9	0.86	13.390	0.21	0.4930	0.0089	0.76	2706	15	2582	38	2785	18	2785.0	18.0	7.3	
615_7_6	87.6	1.56	4.227	0.07	0.2856	0.0046	0.67	1678	13	1619	23	1745	22	1745.0	22.0	7.2	
615_7_8	510.0	1.66	0.263	0.00	0.0365	0.0005	0.50	237	4	232	3	278	36	231.5	2.9	2.4	
615_7_9	267.0	1.86	0.315	0.01	0.0439	0.0008	0.62	278	5	277	5	314	41	276.9	5.0	0.5	
615_7_10	164.0	2.43	0.329	0.01	0.0450	0.0011	0.75	290	7	284	7	330	44	283.9	6.9	2.1	
615_7_13	709.0	2.02	3.966	0.09	0.2751	0.0068	0.85	1625	18	1569	35	1695	24	1695.0	24.0	7.4	
615_7_14	74.4	1.20	1.796	0.03	0.1689	0.0027	0.38	1043	12	1006	15	1114	37	1114.0	37.0	9.7	
615_7_15	282.0	3.64	4.426	0.04	0.2926	0.0025	0.57	1717	8	1654	12	1784	15	1784.0	15.0	7.3	
615_7_16	96.1	1.09	3.785	0.05	0.2735	0.0040	0.70	1589	10	1558	21	1620	20	1620.0	20.0	3.8	
615_7_18	148.3	0.99	0.075	0.00	0.0110	0.0003	0.16	73	4	70	2	180	110	70.2	2.2	4.1	
615_7_19	1152.0	10.10	3.905	0.04	0.2711	0.0026	0.86	1615	8	1546	13	1708.9	9.8	1708.9	9.8	9.5	
615_7_20	240.0	1.27	0.073	0.00	0.0107	0.0002	0.27	71	3	69	2	155	71	68.9	1.5	3.2	
615_7_21	219.9	1.25	0.782	0.01	0.0940	0.0013	0.56	586	7	579	8	618	33	579.0	7.9	1.2	
615_7_22	625.0	0.71	0.178	0.00	0.0246	0.0004	0.37	166	4	157	3	310	49	156.6	2.5	5.8	
615_7_23	68.8	0.49	4.784	0.09	0.3011	0.0059	0.81	1780	16	1696	29	1896	22	1896.0	22.0	10.5	
615_7_24	448.0	6.83	0.225	0.01	0.0315	0.0003	0.13	206	5	200	2	269	66	199.9	2.0	2.9	
615_7_28	223.3	1.67	0.031	0.00	0.0045	0.0001	0.08	31	1	29	1	210	100	29.1	0.9	6.7	
615_7_30	2.4	1.16	6.870	0.99	0.3760	0.0280	0.14	2060	130	2050	130	2010	190	2010.0	190.0	2.0	
615_7_33	123.9	0.88	3.903	0.05	0.2758	0.0040	0.75	1614	10	1570	20	1660	17	1660.0	17.0	5.4	
615_7_34	246.0	1.85	0.107	0.00	0.0161	0.0003	0.36	104	2	103	2	107	46	102.8	1.9	0.7	
615_7_35	49.0	0.56	0.074	0.01	0.0104	0.0004	0.07	72	6	67	2	200	170	66.5	2.4	7.5	
615_7_36	322.0	1.48	4.455	0.05	0.3037	0.0039	0.80	1723	9	1712	20	1742	14	1742.0	14.0	1.7	
615_7_37	227.1	0.99	3.520	0.14	0.2540	0.0077	0.85	1526	32	1458	40	1612	41	1612.0	41.0	9.6	
615_7_38	68.3	0.85	8.930	0.14	0.4375	0.0072	0.71	2336	14	2338	32	2320	21	2320.0	21.0	0.8	
615_7_39	144.2	1.00	3.902	0.05	0.2645	0.0042	0.71	1615	12	1516	22	1737	19	1737.0	19.0	12.7	
615_7_41	216.7	1.62	0.676	0.02	0.0787	0.0024	0.39	524	13	488	14	680	80	488.0	14.0	6.9	
615_7_43	121.0	1.55	4.288	0.07	0.2955	0.0048	0.75	1692	13	1668	24	1719	21	1719.0	21.0	3.0	
615_7_44	280.0	1.45	1.819	0.04	0.1760	0.0040	0.71	1053	14	1045	22	1053	37	1053.0	37.0	0.8	
615_7_45	103.3	0.72	12.590	0.18	0.4893	0.0076	0.73	2651	14	2567	33	2713	19	2713.0	19.0	5.4	
615_7_46	66.8	0.79	5.254	0.08	0.3295	0.0061	0.77	1860	14	1835	30	1890	21	1890.0	21.0	2.9	

	[U] ppm	U/Th	$^{207}Pb/^{235}U$	2σ error	$^{206}Pb/^{238}U$	2σ error	RHO	$^{207}Pb/^{235}U$ Age Ma	2σ error	$^{206}Pb/^{238}U$ Age (Ma)	2σ error	$^{207}Pb/^{206}Pb$ Age (Ma)	2σ error	Best age (Ma)	2σ error	% Disc*	Rim or core
615_7_47	294.7	1.86	3.910	0.12	0.2612	0.0082	0.87	1613	24	1495	42	1771	30	1771.0	30.0	15.6	
615_7_48	131.2	1.78	3.922	0.07	0.2771	0.0054	0.81	1617	15	1576	27	1663	21	1663.0	21.0	5.2	
615_7_49	194.4	0.89	0.091	0.01	0.0133	0.0006	0.41	90	5	85	4	200	110	84.8	3.5	5.3	
615_7_50	338.0	1.18	0.081	0.00	0.0122	0.0002	0.20	79	3	78	1	113	63	78.3	1.4	0.9	
615_7_51	215.9	2.92	4.594	0.07	0.3060	0.0046	0.75	1749	12	1724	22	1763	19	1763.0	19.0	2.2	
615_7_52	335.0	3.09	0.113	0.00	0.0169	0.0005	0.48	109	4	108	3	100	80	107.8	2.8	0.8	Rim
615_7_52	331.0	5.77	3.310	0.12	0.2253	0.0089	0.79	1482	28	1309	47	1715	41	1715.0	41.0	23.7	Core
615_7_53	58.4	1.07	2.887	0.06	0.2279	0.0054	0.85	1380	17	1329	31	1442	32	1442.0	32.0	7.8	
615_7_54	290.0	1.73	0.111	0.00	0.0164	0.0003	0.33	107	3	105	2	128	55	104.9	1.9	1.8	
615_7_55	52.2	1.36	0.081	0.01	0.0114	0.0004	0.07	79	6	73	2	210	140	72.7	2.4	7.9	
615_7_56	206.6	1.28	0.079	0.00	0.0114	0.0004	0.57	77	3	73	2	150	78	72.9	2.3	5.4	
615_7_57	195.0	1.50	2.227	0.04	0.1962	0.0043	0.65	1191	13	1154	23	1252	37	1252.0	37.0	7.8	
615_7_58	457.0	1.84	0.061	0.00	0.0091	0.0002	0.49	60	2	59	2	151	71	58.5	1.5	2.8	
615_7_59	130.7	1.39	2.969	0.05	0.2365	0.0046	0.83	1398	13	1368	24	1446	17	1446.0	17.0	5.4	
615_7_60	186.8	1.94	0.062	0.00	0.0087	0.0002	0.16	61	4	56	1	240	120	56.0	1.4	8.2	
615_7_61	37.2	2.29	0.568	0.02	0.0731	0.0015	0.11	456	11	455	9	433	83	454.9	9.0	0.2	
615_7_62	83.4	3.21	1.709	0.03	0.1660	0.0027	0.02	1011	12	990	15	1046	33	1046.0	33.0	5.4	
615_7_63	110.2	2.27	3.004	0.05	0.2392	0.0037	0.73	1407	13	1382	19	1442	22	1442.0	22.0	4.2	
615_7_64	174.6	1.15	15.420	0.24	0.5490	0.0110	0.82	2839	15	2818	44	2851	20	2851.0	20.0	1.2	
615_7_65	557.0	0.99	0.094	0.00	0.0137	0.0002	0.24	91	3	88	2	163	59	88.0	1.5	3.1	
615_7_66	358.0	3.69	4.320	0.16	0.2781	0.0064	0.79	1696	30	1582	32	1813	43	1813.0	43.0	12.7	Rim
615_7_66	285.0	1.68	9.120	0.13	0.4078	0.0058	0.81	2349	13	2205	26	2484	18	2484.0	18.0	11.2	Core
615_7_67	174.0	1.43	0.039	0.00	0.0055	0.0002	0.09	38	2	35	1	230	140	35.2	1.1	8.1	
615_7_68	138.0	1.01	0.523	0.01	0.0670	0.0012	0.46	427	7	418	7	511	47	418.0	7.4	2.1	
615_7_70A_out	175.3	3.46	0.073	0.00	0.0105	0.0003	0.05	71	4	67	2	200	140	67.3	1.8	5.2	
615_7_71	349.0	2.34	0.074	0.00	0.0111	0.0003	0.37	72	2	71	2	142	77	71.4	1.9	0.8	
615_7_72	194.8	1.06	4.291	0.07	0.2998	0.0056	0.79	1690	14	1689	28	1714	24	1714.0	24.0	1.5	
615_7_73	105.6	1.51	0.064	0.00	0.0095	0.0002	0.03	62	5	61	2	120	130	60.6	1.5	2.9	
615_7_74	85.1	0.83	1.915	0.03	0.1823	0.0030	0.63	1087	11	1079	16	1123	25	1123.0	25.0	3.9	
615_7_75	171.0	2.51	0.208	0.00	0.0303	0.0005	0.43	192	4	192	3	183	48	192.1	3.3	0.1	
615_7_76	168.0	1.15	0.114	0.01	0.0162	0.0008	0.35	110	8	104	5	240	160	103.7	4.7	5.5	
615_7_78	180.5	0.69	1.685	0.05	0.1589	0.0054	0.79	1000	18	954	30	1113	39	1113.0	39.0	14.3	
615_7_79	280.0	0.77	0.082	0.00	0.0114	0.0003	0.04	80	3	73	2	293	86	73.3	1.9	8.6	
615_7_80	195.3	1.11	0.443	0.01	0.0595	0.0008	0.49	372	5	372	5	386	36	372.3	4.9	0.0	
615_7_81	249.0	1.17	0.069	0.00	0.0105	0.0003	0.27	68	3	68	2	98	82	67.6	1.8	0.0	

	[U] ppm	U/Th	$^{207}Pb/^{235}U$	2σ error	$^{206}Pb/^{238}U$	2σ error	RHO	$^{207}Pb/^{235}U$ Age Ma	2σ error	$^{206}Pb/^{238}U$ Age (Ma)	2σ error	$^{207}Pb/^{206}Pb$ Age (Ma)	2σ error	Best age (Ma)	2σ error	% Disc*	Rim or core
615_7_82	145.5	1.57	0.086	0.00	0.0126	0.0004	0.26	84	3	81	2	148	81	80.8	2.2	3.6	
615_7_83	475.0	2.64	0.516	0.01	0.0625	0.0012	0.51	422	8	392	8	614	46	392.1	7.7	7.2	
615_7_85	235.0	1.71	3.685	0.06	0.2648	0.0045	0.83	1570	12	1514	23	1634	16	1634.0	16.0	7.3	
615_7_86	232.0	89.00	0.601	0.02	0.0732	0.0034	0.77	478	14	455	20	545	80	455.0	20.0	4.8	Rim
615_7_86	12.1	2.44	1.437	0.07	0.1471	0.0047	0.04	901	29	885	26	930	130	930.0	130.0	4.8	Core
615_7_87	149.6	1.19	0.099	0.00	0.0143	0.0004	0.27	96	4	91	3	244	84	91.2	2.6	5.4	
615_7_89	68.8	0.73	5.160	0.15	0.3180	0.0110	0.71	1845	26	1780	54	1955	41	1955.0	41.0	9.0	
615_7_90	441.0	2.73	3.814	0.07	0.2625	0.0063	0.88	1596	16	1501	32	1713	19	1713.0	19.0	12.4	
615_7_92	86.5	1.02	2.119	0.04	0.1909	0.0044	0.73	1155	12	1125	24	1215	29	1215.0	29.0	7.4	
615_7_93	306.0	0.68	0.032	0.00	0.0046	0.0002	0.10	32	3	29	1	260	210	29.3	1.4	8.7	
615_7_94	665.0	5.20	0.444	0.01	0.0593	0.0009	0.56	373	4	371	5	377	24	371.4	5.4	0.5	
615_7_95	383.0	0.77	0.187	0.00	0.0272	0.0005	0.51	174	3	173	3	171	43	172.7	2.8	0.7	
615_7_96	122.8	0.56	0.571	0.01	0.0734	0.0010	0.29	458	7	456	6	468	45	456.4	6.0	0.4	
615_7_97	240.0	5.37	4.505	0.07	0.3090	0.0050	0.75	1731	12	1739	25	1714	23	1714.0	23.0	1.5	
615_7_99	483.0	0.93	0.117	0.00	0.0169	0.0003	0.52	113	3	108	2	169	57	108.3	2.1	3.8	
615_7_100	98.0	1.73	1.458	0.02	0.1491	0.0025	0.55	912	9	896	14	942	32	942.0	32.0	4.9	
615_7_101	130.0	2.17	0.067	0.00	0.0101	0.0003	0.28	66	3	65	2	134	91	64.9	1.9	1.2	
615_7_102	173.0	5.50	4.319	0.05	0.2969	0.0038	0.69	1696	10	1675	19	1712	17	1712.0	17.0	2.2	
615_7_104	217.4	1.43	4.616	0.07	0.3029	0.0052	0.52	1750	13	1708	25	1797	20	1797.0	20.0	5.0	
615_7_106	144.0	3.81	14.860	0.24	0.5750	0.0120	0.88	2805	16	2926	49	2702	15	2702.0	15.0	8.3	
615_7_107	208.0	1.49	0.073	0.00	0.0113	0.0002	0.46	72	2	72	1	70	71	72.2	1.4	0.4	
615_7_108	214.0	1.91	1.952	0.09	0.1665	0.0040	0.66	1098	30	993	22	1311	67	1311.0	67.0	24.3	
615_7_109	1001.0	2.47	0.068	0.00	0.0104	0.0001	0.44	67	1	67	1	109	39	66.5	0.9	0.3	
615_7_110	1029.0	1.19	5.010	0.16	0.2930	0.0110	0.81	1824	27	1656	53	2014	38	2014.0	38.0	17.8	
615_7_111	547.0	2.87	0.073	0.00	0.0105	0.0003	0.48	72	2	68	2	207	68	67.5	1.9	6.1	
615_7_112	55.5	1.68	2.122	0.06	0.1893	0.0046	0.55	1155	19	1123	27	1231	54	1231.0	54.0	8.8	
615_7_113	268.0	0.88	0.596	0.01	0.0747	0.0010	0.64	475	5	464	6	516	25	464.2	6.1	2.3	
615_7_115	275.3	2.07	1.979	0.03	0.1818	0.0022	0.65	1108	9	1076	12	1183	22	1183.0	22.0	9.0	
615_7_116	186.0	1.47	3.192	0.06	0.2448	0.0058	0.85	1457	15	1410	30	1527	25	1527.0	25.0	7.7	
615_7_117	357.0	2.68	0.121	0.00	0.0180	0.0004	0.61	116	3	115	2	143	44	115.2	2.3	0.5	
615_7_118	121.4	0.93	0.790	0.01	0.0947	0.0016	0.62	591	8	585	9	605	31	584.5	9.3	1.0	
615_7_119	461.0	0.96	0.172	0.00	0.0250	0.0004	0.57	161	3	159	3	161	37	159.3	2.7	1.2	
615_7_120	38.7	1.30	3.265	0.06	0.2536	0.0054	0.63	1473	14	1456	28	1481	33	1481.0	33.0	1.7	
615_7_121	164.2	1.40	3.347	0.08	0.2515	0.0058	0.80	1492	18	1445	30	1573	25	1573.0	25.0	8.1	
615_7_122	244.0	3.34	4.145	0.07	0.2951	0.0070	0.81	1662	15	1666	35	1648	25	1648.0	25.0	1.1	

	[U] ppm	U/Th	$^{207}Pb/^{235}U$	2σ error	$^{206}Pb/^{238}U$	2σ error	RHO	$^{207}Pb/^{235}U$ Age Ma	2σ error	$^{206}Pb/^{238}U$ Age (Ma)	2σ error	$^{207}Pb/^{206}Pb$ Age (Ma)	2σ error	Best age (Ma)	2σ error	% Disc*	Rim or core
615_7_123	85.6	0.92	4.091	0.05	0.2829	0.0044	0.67	1653	10	1606	22	1691	20	1691.0	20.0	5.0	
615_7_124	96.1	0.69	4.041	0.05	0.2880	0.0037	0.59	1644	10	1633	19	1666	21	1666.0	21.0	2.0	
615_7_125	61.7	0.94	1.911	0.04	0.1765	0.0022	0.47	1085	12	1048	12	1144	32	1144.0	32.0	8.4	
615_7_126	102.3	1.07	12.160	0.22	0.4524	0.0093	0.93	2616	17	2404	41	2780	19	2780.0	19.0	13.5	
615_7_128	70.7	0.69	1.898	0.04	0.1775	0.0036	0.62	1079	12	1053	20	1109	33	1109.0	33.0	5.0	
615_7_129	327.0	1.14	3.957	0.09	0.2683	0.0062	0.73	1625	18	1532	31	1750	25	1750.0	25.0	12.5	
615_7_130	133.0	0.94	2.857	0.04	0.2327	0.0040	0.78	1371	10	1348	21	1397	21	1397.0	21.0	3.5	
615_7_132	647.0	1.42	3.230	0.11	0.2289	0.0088	0.80	1462	27	1328	46	1682	42	1682.0	42.0	21.0	
615_7_133	309.8	1.67	5.471	0.09	0.3256	0.0063	0.89	1895	14	1816	31	1968	17	1968.0	17.0	7.7	

U-Pb isotopic data for sample 615-8

	[U] ppm	U/Th	$^{207}Pb/^{235}U$	2σ error	$^{206}Pb/^{238}U$	2σ error	RHO	$^{207}Pb/^{235}U$ Age Ma	2σ error	$^{206}Pb/^{238}U$ Age (Ma)	2σ error	$^{207}Pb/^{206}Pb$ Age (Ma)	2σ error	Best age (Ma)	2σ error	% Disc*	Rim or core
615_8_1	40.4	1.54	1.733	0.03	0.1717	0.0024	0.13	1020	11	1023	13	1042	44	1042.0	44.0	1.8	
615_8_2	515.0	3.12	1.706	0.01	0.1697	0.0010	0.58	1011	4	1011	6	1021	11	1021.0	11.0	1.0	
615_8_3	514.0	1.04	0.104	0.00	0.0150	0.0003	0.41	100	4	96	2	167	81	96.1	1.7	4.1	
615_8_4	168.4	1.15	0.076	0.00	0.0113	0.0002	0.37	74	3	73	2	151	85	72.6	1.5	2.0	
615_8_5	162.0	0.65	4.451	0.03	0.3050	0.0020	0.60	1722	6	1716	10	1731	12	1731.0	12.0	0.9	
615_8_6	2050.0	0.61	0.024	0.00	0.0036	0.0001	0.10	24	1	23	1	70	110	23.2	0.6	1.6	
615_8_7	160.5	0.88	4.743	0.03	0.3170	0.0022	0.52	1775	6	1775	11	1778	12	1778.0	12.0	0.2	
615_8_9	51.0	1.21	2.844	0.05	0.2354	0.0028	0.09	1366	13	1362	15	1372	37	1372.0	37.0	0.7	
615_8_10	909.0	2.72	0.057	0.00	0.0084	0.0002	0.23	56	2	54	1	158	96	54.0	1.1	3.4	
615_8_11	104.5	1.00	11.629	0.06	0.4912	0.0034	0.42	2575	5	2576	15	2577	10	2577.0	10.0	0.0	
615_8_12	411.0	3.48	2.747	0.03	0.2283	0.0019	0.61	1341	7	1326	10	1374	15	1374.0	15.0	3.5	
615_8_13	205.0	0.83	0.025	0.00	0.0040	0.0002	0.04	25	2	26	1	30	130	25.9	1.0	2.8	
615_8_14	396.5	2.53	0.076	0.00	0.0116	0.0002	0.20	74	2	75	1	60	46	74.6	1.1	0.7	
615_8_16	1076.0	0.75	0.093	0.00	0.0141	0.0004	0.38	90	3	90	3	128	93	89.9	2.8	0.2	
615_8_20	347.0	2.06	1.916	0.01	0.1825	0.0012	0.69	1087	5	1081	6	1093	11	1093.0	11.0	1.1	
615_8_21	476.4	0.37	0.029	0.00	0.0043	0.0001	0.12	29	2	28	1	180	140	27.7	0.9	5.7	
615_8_22	389.8	0.78	4.363	0.08	0.2965	0.0029	0.51	1704	14	1674	14	1751	24	1751.0	24.0	4.4	
615_8_23	38.1	1.45	3.890	0.13	0.2651	0.0073	0.55	1610	28	1515	37	1708	60	1708.0	60.0	11.3	
615_8_25	75.8	1.83	1.706	0.02	0.1700	0.0017	0.26	1010	8	1012	9	1003	30	1003.0	30.0	0.9	
615_8_26	76.0	0.65	0.087	0.01	0.0125	0.0004	0.02	84	5	80	2	210	120	79.7	2.2	5.5	
615_8_27	109.7	1.50	2.354	0.02	0.2098	0.0019	0.19	1228	7	1228	10	1237	21	1237.0	21.0	0.7	
615_8_28	105.6	1.20	0.260	0.01	0.0369	0.0006	0.12	235	5	233	4	261	66	233.3	3.5	0.6	
615_8_29	105.9	1.41	5.619	0.05	0.3434	0.0027	0.48	1921	7	1903	13	1943	15	1943.0	15.0	2.1	
615_8_30	67.3	1.04	2.095	0.04	0.1912	0.0019	0.21	1148	11	1128	11	1174	34	1174.0	34.0	3.9	
615_8_32	53.9	0.87	0.078	0.01	0.0113	0.0005	0.26	76	6	72	3	180	170	72.1	2.9	5.5	
615_8_33	183.1	1.61	5.137	0.04	0.3294	0.0021	0.48	1842	6	1835	10	1867	11	1867.0	11.0	1.7	
615_8_35	205.1	2.97	2.901	0.02	0.2390	0.0014	0.35	1382	5	1381	7	1381	15	1381.0	15.0	0.0	
615_8_36	215.2	2.14	0.088	0.00	0.0132	0.0002	0.12	85	3	84	2	112	79	84.4	1.5	0.9	
615_8_37	390.2	1.77	0.103	0.00	0.0159	0.0004	0.24	100	4	102	2	87	91	101.7	2.4	1.5	
615_8_38	190.4	1.13	0.039	0.00	0.0056	0.0002	0.10	39	2	36	1	250	120	35.8	1.0	7.7	
615_8_39	102.8	1.47	2.102	0.03	0.1908	0.0024	0.28	1149	11	1125	13	1207	28	1207.0	28.0	6.8	
615_8_40	229.2	1.79	0.083	0.00	0.0120	0.0002	0.35	81	3	77	2	176	75	76.9	1.5	5.2	
615_8_41	171.5	1.26	4.274	0.03	0.3020	0.0023	0.48	1688	6	1701	12	1685	15	1685.0	15.0	0.9	

	[U] ppm	U/Th	$^{207}Pb/^{235}U$	2σ error	$^{206}Pb/^{238}U$	2σ error	RHO	$^{207}Pb/^{235}U$ Age Ma	2σ error	$^{206}Pb/^{238}U$ Age (Ma)	2σ error	$^{207}Pb/^{206}Pb$ Age (Ma)	2σ error	Best age (Ma)	2σ error	% Disc*	Rim or core
615_8_42	77.7	1.09	2.912	0.04	0.2410	0.0023	0.48	1384	11	1392	12	1377	26	1377.0	26.0	1.1	
615_8_43	141.6	0.74	0.075	0.00	0.0111	0.0003	0.11	73	3	71	2	151	97	71.3	1.9	2.9	
615_8_44	52.2	0.74	4.109	0.06	0.2902	0.0031	0.37	1655	12	1642	16	1681	28	1681.0	28.0	2.3	
615_8_45	140.0	2.06	0.626	0.02	0.0781	0.0012	0.37	493	10	485	7	544	56	484.8	7.0	1.7	Rim
615_8_45	147.0	1.57	4.649	0.10	0.3063	0.0064	0.63	1758	17	1722	31	1828	18	1828.0	18.0	5.8	Core
615_8_46	82.5	0.75	4.329	0.05	0.3026	0.0028	0.47	1701	9	1704	14	1697	20	1697.0	20.0	0.4	
615_8_47	109.0	1.87	0.082	0.01	0.0116	0.0005	0.20	80	6	75	3	160	170	74.6	3.4	6.6	Rim
615_8_47	113.9	1.03	3.360	0.12	0.2418	0.0085	0.66	1495	28	1396	44	1645	55	1645.0	55.0	15.1	Core
615_8_48	216.8	0.92	0.038	0.00	0.0055	0.0003	0.14	38	4	36	2	200	220	35.6	1.6	6.8	
615_8_49	151.9	1.33	2.896	0.03	0.2353	0.0018	0.31	1381	6	1362	10	1417	17	1417.0	17.0	3.9	
615_8_50	108.1	1.56	1.926	0.02	0.1802	0.0015	0.21	1090	7	1068	8	1134	25	1134.0	25.0	5.8	
615_8_51	267.0	1.08	0.073	0.00	0.0113	0.0002	0.03	72	2	72	1	79	75	72.4	1.2	1.3	
615_8_52	660.0	2.37	0.094	0.00	0.0146	0.0002	0.24	91	2	94	1	76	44	93.5	1.2	2.4	
615_8_53	99.6	1.05	1.182	0.02	0.1284	0.0013	0.04	792	7	779	7	833	34	778.5	7.4	1.7	
615_8_55	264.0	1.72	0.101	0.00	0.0152	0.0003	0.01	98	4	97	2	118	94	97.1	2.1	0.5	
615_8_57	214.0	2.18	0.105	0.00	0.0156	0.0003	0.07	102	3	100	2	130	72	99.6	1.6	2.0	
615_8_58	125.8	1.33	3.231	0.04	0.2627	0.0024	0.40	1466	10	1503	12	1428	23	1428.0	23.0	5.3	
615_8_59	147.0	0.73	0.076	0.00	0.0113	0.0003	0.15	74	3	72	2	130	100	72.3	1.8	2.0	
615_8_61	286.4	0.96	0.088	0.00	0.0133	0.0003	0.23	86	3	85	2	143	88	84.9	1.7	0.8	
615_8_62	60.9	1.28	0.079	0.01	0.0114	0.0004	0.03	77	5	73	2	260	150	72.8	2.3	5.3	
615_8_63	74.8	3.16	4.702	0.04	0.3118	0.0030	0.12	1768	8	1749	15	1784	17	1784.0	17.0	2.0	
615_8_64	188.1	2.28	0.051	0.00	0.0083	0.0002	0.02	51	3	53	1	0	110	53.0	1.2	4.3	
615_8_65	208.0	1.01	0.116	0.00	0.0173	0.0003	0.05	112	3	110	2	170	70	110.2	2.0	1.2	
615_8_66	78.3	0.87	4.514	0.06	0.3093	0.0032	0.47	1733	11	1737	16	1731	22	1731.0	22.0	0.3	
615_8_67	302.0	0.67	0.029	0.00	0.0044	0.0001	0.32	29	1	28	1	110	94	28.1	0.8	3.1	
615_8_68	1391.0	1.54	0.062	0.00	0.0094	0.0002	0.47	61	2	60	1	87	55	60.4	1.1	1.6	
615_8_69	261.0	1.79	0.075	0.00	0.0115	0.0002	0.14	73	4	74	2	67	99	73.8	1.5	0.7	
615_8_71	298.3	1.72	0.071	0.00	0.0108	0.0002	0.13	70	2	70	1	91	76	69.5	1.2	0.3	
615_8_73	160.8	0.92	2.861	0.03	0.2308	0.0022	0.44	1371	8	1339	12	1432	21	1432.0	21.0	6.5	
615_8_74	78.1	0.63	0.165	0.01	0.0256	0.0005	0.11	155	6	163	3	54	91	163.0	3.3	5.2	
615_8_75	391.0	4.01	4.241	0.04	0.2969	0.0027	0.77	1682	7	1676	13	1695	11	1695.0	11.0	1.1	
615_8_76	247.0	1.12	0.094	0.00	0.0134	0.0003	0.13	91	4	86	2	267	99	85.6	1.9	6.3	
615_8_77	253.0	1.24	0.105	0.00	0.0150	0.0003	0.24	101	3	96	2	198	68	95.9	1.7	4.9	
615_8_78	122.0	1.07	0.012	0.00	0.0019	0.0002	0.07	12	2	12	1	-180	310	12.4	1.1	5.1	
615_8_79	218.0	1.14	0.106	0.00	0.0156	0.0003	0.06	102	3	100	2	136	68	99.8	1.6	2.3	

	[U] ppm	U/Th	$^{207}Pb/^{235}U$	2σ error	$^{206}Pb/^{238}U$	2σ error	RHO	$^{207}Pb/^{235}U$ Age Ma	2σ error	$^{206}Pb/^{238}U$ Age (Ma)	2σ error	$^{207}Pb/^{206}Pb$ Age (Ma)	2σ error	Best age (Ma)	2σ error	% Disc*	Rim or core
615_8_80	67.7	1.12	0.084	0.01	0.0121	0.0004	0.07	82	5	77	2	210	130	77.2	2.4	5.5	
615_8_81	277.0	1.69	0.094	0.00	0.0142	0.0003	0.25	91	4	91	2	155	93	90.9	1.8	0.3	
615_8_82	84.3	0.67	0.085	0.01	0.0118	0.0004	0.21	82	6	76	3	350	160	75.7	2.6	7.8	
615_8_83	58.6	0.91	1.930	0.03	0.1849	0.0021	0.40	1090	11	1093	11	1095	31	1095.0	31.0	0.2	
615_8_87	427.0	1.67	0.110	0.00	0.0150	0.0002	0.07	106	3	96	2	348	74	96.1	1.5	9.0	
615_8_88	111.5	1.38	4.297	0.04	0.2925	0.0029	0.55	1694	8	1654	14	1741	18	1741.0	18.0	5.0	
615_8_89	139.5	1.14	3.968	0.04	0.2803	0.0022	0.48	1627	8	1593	11	1674	16	1674.0	16.0	4.8	
615_8_90	502.4	2.16	2.652	0.02	0.2149	0.0018	0.47	1315	5	1255	10	1432	18	1432.0	18.0	12.4	
615_8_91	114.9	1.68	1.778	0.02	0.1761	0.0017	0.07	1038	7	1045	10	1021	27	1021.0	27.0	2.4	
615_8_92	72.7	1.18	10.190	0.27	0.3860	0.0100	0.76	2457	22	2103	47	2767	30	2767.0	30.0	24.0	
615_8_93	100.3	1.69	0.065	0.00	0.0103	0.0004	0.07	64	5	66	2	50	150	66.1	2.4	3.8	
615_8_94	41.7	0.81	12.830	0.13	0.5027	0.0050	0.44	2667	10	2625	21	2698	17	2698.0	17.0	2.7	
615_8_95	208.5	3.04	1.655	0.02	0.1608	0.0017	0.39	993	9	961	9	1072	25	1072.0	25.0	10.4	
615_8_96	373.0	0.71	0.090	0.00	0.0125	0.0003	0.18	87	4	80	2	290	120	80.2	2.0	8.0	
615_8_97	68.8	1.41	0.073	0.01	0.0109	0.0004	0.13	71	5	70	3	110	150	69.9	2.6	1.5	
615_8_98	290.5	1.02	4.789	0.03	0.3082	0.0019	0.56	1784	5	1732	10	1846	12	1846.0	12.0	6.2	
615_8_99	66.0	0.37	2.238	0.03	0.2000	0.0020	0.32	1194	9	1175	11	1224	26	1224.0	26.0	4.0	
615_8_101	90.5	0.80	0.074	0.00	0.0118	0.0004	0.07	72	4	76	2	10	120	75.6	2.2	4.7	
615_8_102	35.6	1.18	13.300	0.14	0.5187	0.0057	0.54	2704	10	2693	24	2711	17	2711.0	17.0	0.7	
615_8_103	232.0	1.37	0.106	0.00	0.0155	0.0003	0.23	103	4	99	2	182	93	98.9	2.0	3.5	
615_8_104	95.0	0.84	0.017	0.00	0.0024	0.0001	0.01	17	2	15	1	280	270	15.5	0.8	8.0	
615_8_105	28.3	0.63	9.150	0.12	0.4396	0.0050	0.47	2351	12	2348	22	2352	21	2352.0	21.0	0.2	
615_8_107	242.0	2.08	0.095	0.00	0.0144	0.0003	0.01	92	3	92	2	133	67	92.3	1.7	0.2	
615_8_108	72.3	3.54	2.491	0.04	0.2170	0.0028	0.31	1270	10	1268	15	1262	32	1262.0	32.0	0.5	
615_8_109	73.2	1.55	0.974	0.02	0.1107	0.0017	0.32	690	10	677	10	731	47	676.7	9.7	1.9	
615_8_110	154.0	1.41	3.414	0.03	0.2626	0.0025	0.40	1507	6	1503	13	1506	16	1506.0	16.0	0.2	
615_8_111	52.1	1.24	2.730	0.03	0.2308	0.0027	0.28	1337	8	1338	14	1361	28	1361.0	28.0	1.7	
615_8_112	197.7	1.71	0.273	0.02	0.0391	0.0027	0.70	245	14	247	17	300	150	247.0	17.0	0.8	Rim
615_8_112	179.4	2.75	2.890	0.10	0.2112	0.0065	0.91	1380	28	1234	35	1606	25	1606.0	25.0	23.2	Core
615_8_113	60.0	1.81	5.837	0.06	0.3485	0.0041	0.60	1953	10	1927	20	1966	19	1966.0	19.0	2.0	
615_8_114	89.9	0.86	4.186	0.05	0.2967	0.0035	0.73	1670	10	1675	17	1659	19	1659.0	19.0	1.0	
615_8_117	22.4	0.49	14.100	0.18	0.5315	0.0068	0.34	2757	12	2746	29	2744	22	2744.0	22.0	0.1	
615_8_118	22.0	1.13	1.685	0.09	0.1693	0.0042	0.18	999	35	1008	23	950	110	950.0	110.0	6.1	
615_8_119	156.3	1.81	4.617	0.04	0.3041	0.0029	0.40	1752	7	1711	14	1798	16	1798.0	16.0	4.8	
615_8_120	72.7	1.80	3.036	0.04	0.2451	0.0017	0.03	1416	10	1413	9	1408	25	1408.0	25.0	0.4	

	[U] ppm	U/Th	$^{207}Pb/^{235}U$	2σ error	$^{206}Pb/^{238}U$	2σ error	RHO	$^{207}Pb/^{235}U$ Age Ma	2σ error	$^{206}Pb/^{238}U$ Age (Ma)	2σ error	$^{207}Pb/^{206}Pb$ Age (Ma)	2σ error	Best age (Ma)	2σ error	% Disc*	Rim or core
615_8_121	169.3	1.02	4.332	0.03	0.2978	0.0024	0.39	1699	7	1680	12	1715	16	1715.0	16.0	2.0	
615_8_122	123.4	1.02	5.003	0.04	0.3231	0.0027	0.51	1820	6	1804	13	1838	15	1838.0	15.0	1.8	
615_8_123	124.0	0.58	0.246	0.01	0.0345	0.0005	0.21	223	6	219	3	253	58	218.5	3.2	2.1	
615_8_124	551.0	1.19	0.101	0.00	0.0152	0.0002	0.19	98	2	97	1	126	43	97.2	1.1	0.7	
615_8_125	177.0	2.13	0.206	0.01	0.0304	0.0004	0.29	191	5	193	3	161	63	192.8	2.7	1.2	
615_8_126	90.2	1.67	12.760	0.12	0.4989	0.0055	0.70	2662	9	2609	24	2703	11	2703.0	11.0	3.5	
615_8_127	80.1	1.07	1.661	0.03	0.1619	0.0025	0.56	994	13	967	14	1057	29	1057.0	29.0	8.5	
615_8_128	259.0	0.74	0.013	0.00	0.0020	0.0001	0.31	14	1	13	1	140	190	13.0	0.6	4.0	
615_8_129	550.0	1.19	0.098	0.00	0.0148	0.0002	0.05	94	2	95	1	102	54	94.7	1.3	0.3	
615_8_130	61.3	0.99	14.250	0.10	0.5363	0.0044	0.44	2767	7	2767	19	2761	12	2761.0	12.0	0.2	

U-Pb isotopic data for sample 615-9

	[U] ppm	U/Th	$^{207}Pb/^{235}U$	2σ error	$^{206}Pb/^{238}U$	2σ error	RHO	$^{207}Pb/^{235}U$ Age Ma	2σ error	$^{206}Pb/^{238}U$ Age (Ma)	2σ error	$^{207}Pb/^{206}Pb$ Age (Ma)	2σ error	Best age (Ma)	2σ error	% Disc*	Rim or core
615-9-b_2	0.1	no value	no value	NAN	no value	NAN	#VALUE!	no value	NAN	no value	NAN	no value	NAN				
615-9-b_3	47.4	29.00	2.343	0.08	0.2282	0.0067	0.51	1220	24	1322	35	1047	66	1047.0	66.0	26.3	
615-9-b_4	51.9	0.07	0.847	0.04	0.1052	0.0040	0.21	620	23	644	23	550	110	644.0	23.0	3.9	
615-9-b_5	63.5	1.17	4.749	0.10	0.3240	0.0062	0.47	1773	17	1807	30	1744	37	1744.0	37.0	3.6	
615-9-b_6	241.0	1.42	3.218	0.06	0.2648	0.0047	0.52	1459	14	1513	24	1390	34	1390.0	34.0	8.8	
615-9-b_7	79.0	0.92	4.605	0.08	0.3287	0.0045	0.51	1747	15	1831	22	1674	31	1674.0	31.0	9.4	
615-9-b_8	63.2	3.12	1.937	0.06	0.1922	0.0060	0.44	1086	22	1135	33	1027	73	1027.0	73.0	10.5	
615-9-b_9	0.1	no value	no value	NAN	no value	NAN	#VALUE!	no value	NAN	no value	NAN	no value	NAN				
615-9-b_10	38.3	0.12	0.707	0.05	0.0096	0.0006	0.11	535	30	61	4	4310	140	DISC	DISC	88.5	
615-9-b_11	191.0	3.30	0.119	0.03	0.0086	0.0008	0.59	105	18	55	5	1180	260	DISC	DISC	47.2	
615-9-b_12	103.0	1.56	4.840	0.14	0.3122	0.0083	0.70	1786	24	1749	41	1838	38	1838.0	38.0	4.8	
615-9-b_13	72.0	1.82	0.251	0.02	0.0355	0.0011	0.01	224	18	225	7	200	170	224.6	6.9	0.3	
615-9-b_14	560.0	4.52	4.470	0.11	0.3130	0.0091	0.65	1719	20	1751	45	1708	44	1708.0	44.0	2.5	
615-9-b_15	0.7	no value	no value	NAN	no value	NAN	#VALUE!	no value	NAN	no value	NAN	no value	NAN				
615-9-b_16	143.4	1.57	1.980	0.04	0.1900	0.0030	0.50	1107	14	1121	16	1076	36	1076.0	36.0	4.2	
615-9-b_17	501.0	1.28	4.878	0.08	0.3424	0.0067	0.74	1798	15	1896	32	1692	25	1692.0	25.0	12.1	
615-9-b_18	-0.1	no value	no value	NAN	no value	NAN	#VALUE!	no value	NAN	no value	NAN	no value	NAN				
615-9-b_19	1010.0	5.18	1.457	0.04	0.0959	0.0027	0.34	910	16	590	16	1814	53	DISC	DISC	35.2	
615-9-b_20	0.1	no value	no value	NAN	no value	NAN	#VALUE!	no value	NAN	no value	NAN	no value	NAN				
615-9-b_21	0.3	no value	no value	NAN	no value	NAN	#VALUE!	no value	NAN	no value	NAN	no value	NAN				
615-9-b_22	109.6	2.11	3.195	0.06	0.2539	0.0031	0.42	1453	14	1458	16	1426	32	1426.0	32.0	2.2	
615-9-b_23	202.0	1.17	1.989	0.05	0.1868	0.0051	0.73	1107	17	1102	27	1094	37	1094.0	37.0	0.7	
615-9-b_24	44.4	0.13	1.077	0.05	0.0114	0.0007	0.04	736	24	73	4	4750	150	DISC	DISC	90.0	
615-9-b_25	890.0	1.70	0.035	0.00	0.0046	0.0001	0.18	34	2	30	1	300	120	DISC	DISC	13.0	
615-9-b_26	58.2	2.22	2.770	0.10	0.2115	0.0061	0.40	1341	29	1234	33	1491	65	1491.0	65.0	17.2	
615-9-b_27	82.9	1.68	1.809	0.07	0.1747	0.0038	0.56	1042	26	1037	21	1042	64	1042.0	64.0	0.5	
615-9-b_28	67.6	2.44	2.292	0.07	0.2121	0.0029	0.19	1204	20	1240	16	1117	59	1117.0	59.0	11.0	
615-9-b_29	0.1	no value	no value	NAN	no value	NAN	#VALUE!	no value	NAN	no value	NAN	no value	NAN	#VALUE!	#VALUE!	#VALUE!	

	[U] ppm	U/Th	$^{207}Pb/^{235}U$	2σ error	$^{206}Pb/^{238}U$	2σ error	RHO	$^{207}Pb/^{235}U$ Age Ma	2σ error	$^{206}Pb/^{238}U$ Age (Ma)	2σ error	$^{207}Pb/^{206}Pb$ Age (Ma)	2σ error	Best age (Ma)	2σ error	% Disc*	Rim or core
615-9-b_30	305.0	4.94	2.032	0.05	0.1761	0.0044	0.74	1122	17	1044	24	1280	36	1280.0	36.0	18.4	
615-9-b_31	290.0	1.86	14.510	0.35	0.5490	0.0150	0.77	2779	24	2816	62	2757	29	2757.0	29.0	2.1	
615-9-b_32	333.0	1.53	0.050	0.00	0.0076	0.0002	0.62	49	5	49	1	70	160	49.1	1.4	0.2	
615-9-b_33	267.1	2.13	10.710	0.23	0.4553	0.0099	0.65	2494	20	2415	44	2566	30	2566.0	30.0	5.9	
615-9-b_34	7.8	4.40	2.930	0.19	0.0357	0.0023	0.25	1354	51	225	14	4480	150	DISC	DISC	83.4	
615-9-b_35	38.7	2.16	16.040	0.53	0.5610	0.0160	0.66	2868	31	2862	68	2879	39	2879.0	39.0	0.6	
615-9-b_36	322.0	17.50	1.979	0.07	0.1859	0.0059	0.57	1103	25	1104	34	1133	54	1133.0	54.0	2.6	
615-9-b_37	64.4	1.27	4.230	0.11	0.3060	0.0062	0.32	1673	22	1719	30	1628	53	1628.0	53.0	5.6	
615-9-b_38	606.0	2.35	0.034	0.00	0.0047	0.0002	0.29	33	3	30	1	240	140	DISC	DISC	10.2	
615-9-b_39	640.0	1.47	0.027	0.00	0.0042	0.0001	0.06	27	2	27	1	50	130	27.1	0.7	1.0	
615-9-b_40	0.1	no value	no value	NAN	no value	NAN	#VAL-UE!	no value	NAN	no value	NAN	no value	NAN	#VAL-UE!	#VAL-UE!	#VAL-UE!	
615-9-b_41	194.9	1.89	0.026	0.00	0.0041	0.0002	0.02	25	4	26	1	-10	240	26.3	1.2	3.5	
615-9-b_42	359.0	3.24	3.167	0.08	0.2465	0.0059	0.66	1443	20	1419	30	1488	40	1488.0	40.0	4.6	
615-9-b_43	291.0	3.44	13.630	0.44	0.5220	0.0190	0.65	2722	31	2719	86	2724	50	2724.0	50.0	0.2	
615-9-b_44	33.0	2.58	1.826	0.09	0.1774	0.0042	0.26	1041	30	1055	24	992	95	992.0	95.0	6.4	
615-9-b_45	55.0	0.18	0.762	0.04	0.0127	0.0006	0.18	568	24	81	4	4040	110	DISC	DISC	85.7	
615-9-b_46	239.0	1.56	0.077	0.01	0.0123	0.0004	0.10	75	6	79	2	50	140	78.7	2.4	4.9	
615-9-b_47	179.0	4.87	3.246	0.07	0.2301	0.0041	0.54	1467	15	1334	22	1667	35	1667.0	35.0	20.0	
615-9-b_48	507.0	15.20	2.589	0.07	0.2250	0.0046	0.68	1290	21	1307	24	1279	41	1279.0	41.0	2.2	
615-9-1	233.0	1.11	0.124	0.01	0.0119	0.0003	0.42	118	11	76	2	980	180	DISC	DISC	35.5	
615-9-2	506.0	5.10	1.701	0.02	0.1686	0.0020	0.42	1008	9	1004	11	1037	28	1037.0	28.0	3.2	
615-9-3	325.0	1.77	2.485	0.02	0.2183	0.0013	0.29	1268	7	1273	7	1250	19	1250.0	19.0	1.8	
615-9-5	30.4	0.90	2.026	0.09	0.1885	0.0058	0.59	1119	28	1120	33	1135	69	1135.0	69.0	1.3	
615-9-6	52.5	1.42	3.032	0.07	0.2449	0.0042	0.44	1411	18	1411	22	1453	41	1453.0	41.0	2.9	
615-9-8	282.0	0.63	0.574	0.01	0.0732	0.0008	0.13	460	8	456	5	471	51	455.5	4.6	1.1	
615-9-9	232.1	1.11	30.180	0.32	0.7056	0.0068	0.69	3494	10	3440	26	3523	12	3523.0	12.0	2.4	
615-9-11	18.6	2.64	0.461	0.05	0.0076	0.0008	0.03	362	33	49	5	3970	260	DISC	DISC	86.5	
615-9-12	738.0	0.97	13.420	0.19	0.5020	0.0066	0.88	2710	13	2621	28	2782	11	2782.0	11.0	5.8	
615-9-13	757.0	0.38	0.078	0.00	0.0117	0.0001	0.02	76	2	75	1	114	62	74.9	0.8	1.4	
615-9-15	122.7	2.79	16.260	0.60	0.5970	0.0160	0.84	2889	35	3014	64	2804	34	2804.0	34.0	7.5	
615-9-16	60.7	0.63	3.358	0.07	0.2573	0.0041	0.61	1491	16	1475	21	1534	32	1534.0	32.0	3.8	
615-9-17	186.7	1.52	2.200	0.04	0.1965	0.0028	0.59	1183	12	1156	15	1236	30	1236.0	30.0	6.5	
615-9-18	218.7	2.26	5.010	0.05	0.3250	0.0025	0.55	1820	8	1814	12	1836	16	1836.0	16.0	1.2	

	[U] ppm	U/Th	$^{207}Pb/^{235}U$	2σ error	$^{206}Pb/^{238}U$	2σ error	RHO	$^{207}Pb/^{235}U$ Age Ma	2σ error	$^{206}Pb/^{238}U$ Age (Ma)	2σ error	$^{207}Pb/^{206}Pb$ Age (Ma)	2σ error	Best age (Ma)	2σ error	% Disc*	Rim or core
615-9-19	681.0	0.84	3.043	0.03	0.2428	0.0022	0.46	1418	7	1401	11	1443	18	1443.0	18.0	2.9	
615-9-20	118.3	0.83	4.119	0.06	0.2939	0.0037	0.47	1659	12	1662	19	1651	27	1651.0	27.0	0.7	
615-9-22	137.0	1.56	0.217	0.01	0.0309	0.0006	0.17	198	8	196	4	245	91	196.3	3.8	0.9	
615-9-23	111.2	0.53	13.460	0.21	0.5225	0.0070	0.67	2711	15	2708	30	2707	20	2707.0	20.0	0.0	
615-9-24	213.0	0.76	0.078	0.00	0.0117	0.0003	0.16	76	5	75	2	110	120	74.9	1.6	1.4	
615-9-25	699.0	1.12	2.610	0.03	0.1631	0.0018	0.51	1304	9	974	10	1899	21	DISC	DISC	48.7	
615-9-26	268.6	2.46	4.283	0.09	0.2943	0.0057	0.52	1689	17	1662	28	1756	38	1756.0	38.0	5.4	
615-9-27	7550.0	0.64	0.161	0.01	0.0051	0.0001	0.71	151	5	33	0	3067	49	DISC	DISC	78.4	
615-9-28	190.5	0.36	0.507	0.04	0.0179	0.0006	0.65	414	29	115	4	2850	110	DISC	DISC	72.3	
615-9-29	185.5	1.81	3.587	0.05	0.2665	0.0022	0.42	1546	12	1523	11	1596	28	1596.0	28.0	4.6	
615-9-31	535.0	1.44	0.310	0.02	0.0357	0.0004	0.59	272	12	226	3	690	96	DISC	DISC	16.9	
615-9-32	71.9	1.28	12.230	0.18	0.4940	0.0071	0.63	2622	14	2587	31	2638	20	2638.0	20.0	1.9	
615-9-33	23.0	0.40	11.530	0.42	0.4560	0.0130	0.79	2559	34	2413	59	2679	35	2679.0	35.0	9.9	
615-9-35	421.0	1.29	12.280	0.22	0.3795	0.0045	0.66	2624	17	2073	21	3073	21	DISC	DISC	32.5	
615-9-36	195.7	0.66	12.110	0.12	0.4802	0.0044	0.68	2613	9	2527	19	2680	13	2680.0	13.0	5.7	
615-9-37	129.0	0.95	5.225	0.08	0.3236	0.0035	0.56	1856	13	1807	17	1893	22	1893.0	22.0	4.5	
615-9-38	118.1	0.69	0.026	0.00	0.0034	0.0001	0.05	26	4	22	1	280	270	DISC	DISC	15.2	
615-9-39	47.8	2.72	3.089	0.09	0.2490	0.0059	0.65	1428	23	1431	30	1419	46	1419.0	46.0	0.8	
615-9-40	404.0	1.32	1.668	0.03	0.1334	0.0020	0.61	995	11	808	11	1437	31	DISC	DISC	18.8	
615-9-41	76.8	1.82	1.616	0.04	0.1644	0.0021	0.27	975	17	981	12	959	55	959.0	55.0	2.3	
615-9-42	69.5	0.67	2.129	0.07	0.1756	0.0030	0.41	1156	22	1043	17	1403	58	1403.0	58.0	25.7	
615-9-43	47.6	0.35	0.852	0.04	0.0108	0.0005	0.11	628	20	69	3	4461	91	DISC	DISC	89.0	
615-9-45	131.0	0.64	13.870	0.12	0.5400	0.0051	0.54	2740	9	2783	21	2719	15	2719.0	15.0	2.4	
615-9-46	169.0	0.70	3.160	0.04	0.2551	0.0027	0.45	1447	11	1465	14	1433	27	1433.0	27.0	2.2	
615-9-48	353.0	0.59	0.021	0.00	0.0030	0.0001	0.02	21	2	19	0	280	190	19.2	0.5	7.2	
615-9-49	196.0	1.19	6.685	0.06	0.3789	0.0033	0.57	2071	8	2070	15	2073	14	2073.0	14.0	0.1	
615-9-51	240.8	0.30	4.248	0.07	0.2864	0.0027	0.52	1684	13	1623	14	1756	26	1756.0	26.0	7.6	
615-9-52	100.4	0.53	0.691	0.03	0.0770	0.0014	0.10	533	18	478	8	749	93	DISC	DISC	10.4	
615-9-53	80.9	0.87	10.390	0.16	0.4664	0.0068	0.61	2472	14	2466	30	2480	22	2480.0	22.0	0.6	
615-9-54	214.5	1.73	0.250	0.01	0.0356	0.0005	0.05	228	6	226	3	220	64	225.5	2.8	0.9	
615-9-55	253.0	1.44	3.038	0.05	0.2387	0.0023	0.35	1418	13	1380	12	1484	29	1484.0	29.0	7.0	
615-9-56	128.9	0.13	0.423	0.02	0.0057	0.0003	0.17	359	12	37	2	4349	79	DISC	DISC	89.8	
615-9-57	247.0	1.36	4.156	0.06	0.2928	0.0034	0.59	1666	12	1655	17	1683	23	1683.0	23.0	1.7	
615-9-58	76.4	0.18	9.680	0.17	0.3764	0.0063	0.70	2403	16	2057	29	2696	23	2696.0	23.0	23.7	
615-9-59	281.1	1.06	1.991	0.03	0.1866	0.0014	0.39	1114	8	1103	8	1140	24	1140.0	24.0	3.2	

	[U] ppm	U/Th	$^{207}Pb/^{235}U$	2σ error	$^{206}Pb/^{238}U$	2σ error	RHO	$^{207}Pb/^{235}U$ Age Ma	2σ error	$^{206}Pb/^{238}U$ Age (Ma)	2σ error	$^{207}Pb/^{206}Pb$ Age (Ma)	2σ error	Best age (Ma)	2σ error	% Disc*	Rim or core
615-9-60	64.5	0.62	13.150	0.21	0.5102	0.0075	0.77	2690	15	2655	32	2720	16	2720.0	16.0	2.4	
615-9-61	68.1	0.13	0.753	0.04	0.0127	0.0004	0.05	565	20	81	3	4031	85	DISC	DISC	85.6	
615-9-64	13.3	1.73	5.300	0.48	0.3120	0.0250	0.97	1794	58	1730	120	1973	68	1973.0	68.0	12.3	
615-9-65	72.4	1.59	3.136	0.08	0.2465	0.0039	0.63	1440	20	1420	20	1451	39	1451.0	39.0	2.1	
615-9-66	75.7	0.83	11.340	0.18	0.4637	0.0066	0.71	2549	15	2458	29	2632	19	2632.0	19.0	6.6	
615-9-67	76.6	0.84	3.190	0.07	0.2496	0.0033	0.56	1454	17	1436	17	1457	34	1457.0	34.0	1.4	
615-9-68	64.9	0.99	0.537	0.03	0.0673	0.0012	0.19	437	17	419	7	510	100	419.4	7.1	4.0	
615-9-69	35.0	0.46	4.320	0.11	0.3068	0.0067	0.63	1700	21	1723	33	1665	42	1665.0	42.0	3.5	
615-9-70	121.6	0.90	13.610	0.14	0.5283	0.0053	0.70	2724	10	2733	22	2714	13	2714.0	13.0	0.7	
615-9-71	78.2	0.90	1.642	0.05	0.1519	0.0025	0.39	986	17	911	14	1155	49	1155.0	49.0	21.1	
615-9-72	236.0	0.71	4.756	0.05	0.3158	0.0026	0.49	1777	8	1771	13	1783	17	1783.0	17.0	0.7	
615-9-73	370.1	1.08	4.183	0.04	0.2876	0.0022	0.43	1671	7	1631	11	1717	16	1717.0	16.0	5.0	
615-9-74	561.0	1.85	1.779	0.03	0.1741	0.0026	0.54	1037	10	1034	14	1054	29	1054.0	29.0	1.9	
615-9-76	191.1	0.67	0.269	0.01	0.0368	0.0004	0.11	241	7	233	3	281	69	232.8	2.6	3.6	
615-9-77	450.0	0.95	3.043	0.03	0.2437	0.0021	0.69	1417	9	1405	11	1435	16	1435.0	16.0	2.1	
615-9-78	195.4	1.05	11.480	0.13	0.4942	0.0047	0.71	2564	10	2588	20	2545	12	2545.0	12.0	1.7	
615-9-79	390.0	13.60	0.468	0.03	0.0589	0.0012	0.43	390	18	369	7	460	120	368.9	7.4	5.4	Rim
615-9-79	663.0	5.53	0.942	0.02	0.0989	0.0013	0.70	674	10	608	8	898	39	607.7	7.9	9.8	Core
615-9-79	242.0	1.57	1.460	0.03	0.1421	0.0016	0.24	915	12	857	9	1037	41	1037.0	41.0	17.4	Core
615-9-80	243.3	3.09	12.597	0.09	0.5019	0.0035	0.57	2650	7	2623	15	2673	10	2673.0	10.0	1.9	
615-9-82	38.5	1.57	2.788	0.08	0.2278	0.0047	0.68	1348	21	1322	25	1397	44	1397.0	44.0	5.4	
615-9-84	217.4	1.07	2.866	0.04	0.2342	0.0025	0.48	1373	10	1356	13	1389	22	1389.0	22.0	2.4	
615-9-85	680.0	0.64	0.024	0.00	0.0035	0.0001	0.15	24	2	23	0	130	110	22.8	0.4	4.1	
615-9-86	1114.0	27.40	0.633	0.02	0.0805	0.0019	0.33	497	14	499	11	476	80	499.0	11.0	0.4	Rim
615-9-86	256.0	2.51	2.517	0.04	0.2148	0.0022	0.21	1276	11	1254	12	1315	28	1315.0	28.0	4.6	Core
615-9-87	86.9	0.58	3.566	0.07	0.2568	0.0035	0.43	1540	15	1473	18	1614	32	1614.0	32.0	8.7	
615-9-88	-0.1	-0.14	79.000	79.00	0.3800	0.3800	#VALUE!	4500	4500	2100	2100	6600	6600	DISC	DISC	68.2	
615-9-90	495.0	79.00	0.535	0.01	0.0694	0.0005	0.15	435	5	433	3	434	34	432.7	3.1	0.6	
615-9-91	99.9	1.02	1.604	0.04	0.1570	0.0023	0.46	972	14	940	13	1050	45	1050.0	45.0	10.5	
615-9-94	193.3	0.83	2.000	0.03	0.1817	0.0016	0.33	1118	10	1076	9	1195	30	1195.0	30.0	9.9	
615-9-95	235.2	1.02	11.228	0.09	0.4459	0.0037	0.72	2542	8	2376	17	2676	10	2676.0	10.0	11.2	
615-9-96	882.0	0.78	4.202	0.04	0.2689	0.0023	0.71	1674	8	1535	12	1848	12	1848.0	12.0	16.9	Rim
615-9-96	232.5	0.99	5.241	0.10	0.3309	0.0046	0.53	1861	16	1842	22	1886	29	1886.0	29.0	2.3	Core
615-9-97	489.0	1.34	1.874	0.02	0.1788	0.0012	0.34	1072	8	1060	7	1087	21	1087.0	21.0	2.5	

	[U] ppm	U/Th	$^{207}Pb/^{235}U$	2σ error	$^{206}Pb/^{238}U$	2σ error	RHO	$^{207}Pb/^{235}U$ Age Ma	2σ error	$^{206}Pb/^{238}U$ Age (Ma)	2σ error	$^{207}Pb/^{206}Pb$ Age (Ma)	2σ error	Best age (Ma)	2σ error	% Disc*	Rim or core
615-9-98	274.0	1.07	0.085	0.00	0.0122	0.0002	0.00	83	5	78	1	240	120	77.9	1.2	5.9	
615-9-99	29.7	0.08	1.091	0.06	0.0138	0.0008	0.30	745	27	88	5	4448	96	DISC	DISC	88.2	
615-9-101	716.0	1.35	0.037	0.00	0.0057	0.0001	0.09	37	2	37	1	112	96	36.8	0.6	0.2	
615-9-102	108.8	1.82	0.033	0.00	0.0043	0.0002	0.10	33	4	28	1	270	250	DISC	DISC	14.9	
615-9-103	175.0	0.64	2.056	0.03	0.1890	0.0015	0.25	1135	9	1116	8	1165	27	1165.0	27.0	4.2	
615-9-105	145.5	1.73	1.671	0.03	0.1640	0.0017	0.45	995	13	979	9	1034	35	1034.0	35.0	5.3	
615-9-106	317.5	0.71	2.214	0.03	0.2003	0.0014	0.34	1184	8	1177	8	1201	23	1201.0	23.0	2.0	
615-9-108	72.5	2.97	2.210	0.07	0.2020	0.0032	0.50	1185	20	1186	17	1188	52	1188.0	52.0	0.2	
615-9-109	148.0	2.04	2.976	0.06	0.2301	0.0029	0.38	1400	15	1335	15	1493	34	1493.0	34.0	10.6	
615-9-110	165.3	1.94	4.805	0.06	0.3170	0.0032	0.54	1785	11	1774	15	1788	19	1788.0	19.0	0.8	
615-9-111	196.0	1.03	3.880	0.07	0.2753	0.0033	0.52	1613	15	1568	17	1692	30	1692.0	30.0	7.3	
615-9-112	101.1	0.75	3.009	0.04	0.2446	0.0022	0.29	1409	11	1410	12	1412	28	1412.0	28.0	0.1	
615-9-117	449.0	6.30	2.102	0.04	0.1928	0.0025	0.38	1148	12	1139	14	1159	31	1159.0	31.0	1.7	
615-9-118	65.9	0.52	2.139	0.06	0.1961	0.0042	0.50	1164	20	1153	23	1173	46	1173.0	46.0	1.7	
615-9-119	70.8	0.85	1.995	0.05	0.1887	0.0031	0.51	1112	15	1114	17	1141	43	1141.0	43.0	2.4	
615-9-120	337.0	1.07	2.220	0.02	0.2054	0.0013	0.35	1187	6	1204	7	1163	18	1163.0	18.0	3.5	

U-Pb isotopic data for sample 615-10

	[U] ppm	U/Th	$^{207}Pb/^{235}U$	2σ error	$^{206}Pb/^{238}U$	2σ error	RHO	$^{207}Pb/^{235}U$ Age Ma	2σ error	$^{206}Pb/^{238}U$ Age (Ma)	2σ error	$^{207}Pb/^{206}Pb$ Age (Ma)	2σ error	Best age (Ma)	2σ error	% Disc*	Rim or core
615-10-b_1	171.8	0.69	2.429	0.04	0.2202	0.0024	0.36	1251	12	1282	12	1198	31	1198.0	31.0	7.0	
615-10-b_2	930.0	14.00	2.049	0.08	0.1946	0.0064	0.76	1137	30	1146	34	1095	60	1095.0	60.0	4.7	
615-10-b_2b	107.9	2.06	2.732	0.05	0.2300	0.0038	0.50	1335	14	1334	20	1325	35	1325.0	35.0	0.7	
615-10-b_3	92.9	0.97	14.560	0.16	0.5664	0.0083	0.67	2785	10	2890	34	2710	19	2710.0	19.0	6.6	
615-10-b_4	324.8	2.68	2.302	0.07	0.1919	0.0031	0.14	1207	21	1131	17	1328	61	1328.0	61.0	14.8	
615-10-b_5	295.0	2.14	2.165	0.04	0.2003	0.0028	0.48	1170	12	1177	15	1155	33	1155.0	33.0	1.9	
615-10-b_6	61.7	0.96	4.217	0.10	0.3035	0.0068	0.36	1673	19	1706	34	1632	48	1632.0	48.0	4.5	
615-10-b_7	253.0	1.52	0.082	0.01	0.0126	0.0003	0.19	81	5	81	2	70	110	81.0	1.6	0.6	
615-10-b_8	843.0	1.75	0.104	0.00	0.0157	0.0002	0.20	101	3	100	1	122	58	100.2	1.4	0.5	
615-10-b_9	16.3	0.79	1.960	0.11	0.1830	0.0060	0.55	1094	40	1081	32	1130	120	1130.0	120.0	4.3	
615-10-b_10	55.9	0.11	0.257	0.05	0.0116	0.0009	0.22	223	37	74	5	2140	320	DISC	DISC	66.8	
615-10-b_11	69.2	1.52	1.952	0.05	0.1916	0.0024	0.38	1096	16	1130	13	1021	46	1021.0	46.0	10.7	
615-10-b_12	270.5	1.74	2.256	0.03	0.2093	0.0025	0.51	1198	9	1225	13	1143	26	1143.0	26.0	7.2	
615-10-b_13	0.0	no value	no value	NAN	no value	NAN	#VALUE!	no value	NAN	no value	NAN	no value	NAN	#VALUE!	#VALUE!	#VALUE!	
615-10-b_14	244.0	42.60	2.125	0.05	0.1993	0.0032	0.59	1155	16	1171	17	1114	39	1114.0	39.0	5.1	
615-10-b_15	56.8	1.69	4.836	0.08	0.3292	0.0050	0.49	1791	14	1833	24	1735	30	1735.0	30.0	5.6	
615-10-b_16	0.1	no value	no value	NAN	no value	NAN	#VALUE!	no value	NAN	no value	NAN	no value	NAN	#VALUE!	#VALUE!	#VALUE!	
615-10-b_17	149.1	2.33	4.210	0.06	0.2986	0.0044	0.50	1674	12	1683	22	1644	28	1644.0	28.0	2.4	
615-10-b_18	102.0	91.00	13.750	0.22	0.5449	0.0093	0.55	2730	15	2801	39	2672	26	2672.0	26.0	4.8	
615-10-b_19	116.5	0.96	0.224	0.01	0.0320	0.0007	0.12	204	8	203	4	203	92	203.1	4.4	0.4	
615-10-b_20	214.0	2.08	1.925	0.03	0.1837	0.0021	0.41	1088	10	1087	11	1081	29	1081.0	29.0	0.6	
615-10-b_21	36.5	1.91	1.822	0.06	0.1805	0.0028	0.30	1051	23	1069	15	1001	68	1001.0	68.0	6.8	
615-10-b_22	223.0	1.50	0.204	0.01	0.0297	0.0005	0.19	188	6	189	3	169	70	188.5	3.4	0.5	
615-10-b_23	929.0	3.11	0.075	0.00	0.0115	0.0003	0.26	74	3	74	2	110	80	73.5	1.9	0.3	
615-10-b_24	91.2	4.59	1.920	0.11	0.1880	0.0120	0.49	1073	37	1103	64	1030	110	1030.0	110.0	7.1	
615-10-b_25	554.0	11.90	2.027	0.05	0.1867	0.0042	0.58	1124	16	1102	23	1161	42	1161.0	42.0	5.1	
615-10-b_26	223.1	2.31	2.627	0.06	0.2315	0.0054	0.48	1308	18	1341	28	1254	49	1254.0	49.0	6.9	
615-10-b_27	294.0	2.59	0.078	0.00	0.0111	0.0002	0.03	76	4	71	1	230	100	71.0	1.2	7.1	
615-10-b_28	48.8	0.49	14.150	0.21	0.5430	0.0073	0.62	2757	14	2794	30	2733	20	2733.0	20.0	2.2	
615-10-b_29	27.3	1.70	2.169	0.07	0.1957	0.0038	0.29	1171	21	1151	20	1207	61	1207.0	61.0	4.6	
615-10-b_30	218.0	1.12	2.250	0.06	0.1841	0.0042	0.65	1192	20	1088	23	1389	40	1389.0	40.0	21.7	

	[U] ppm	U/Th	^{207}Pb/^{235}U	2σ error	^{206}Pb/^{238}U	2σ error	RHO	^{207}Pb/^{235}U Age Ma	2σ error	^{206}Pb/^{238}U Age (Ma)	2σ error	^{207}Pb/^{206}Pb Age (Ma)	2σ error	Best age (Ma)	2σ error	% Disc*	Rim or core
615-10-b_31	43.9	1.43	2.961	0.06	0.2450	0.0033	0.36	1394	16	1412	17	1373	40	1373.0	40.0	2.8	
615-10-b_32	34.4	1.23	0.858	0.04	0.1015	0.0022	0.13	622	23	623	13	600	100	623.0	13.0	0.2	
615-10-b_33	63.1	3.49	2.108	0.07	0.2013	0.0056	0.55	1145	23	1180	30	1078	60	1078.0	60.0	9.5	
615-10-b_34	246.0	2.58	0.062	0.00	0.0092	0.0002	0.16	61	4	59	1	140	120	58.9	1.3	3.1	
615-10-b_35	230.9	1.88	0.086	0.01	0.0122	0.0004	0.07	83	5	78	3	220	140	78.1	2.7	5.9	
615-10-b_36	545.0	13.80	2.985	0.05	0.2506	0.0037	0.63	1402	12	1441	19	1354	26	1354.0	26.0	6.4	
615-10-b_37	169.0	3.57	3.541	0.09	0.2719	0.0071	0.74	1535	22	1547	36	1508	37	1508.0	37.0	2.6	
615-10-b_38	58.5	1.03	13.910	0.24	0.5335	0.0094	0.54	2742	16	2753	39	2729	25	2729.0	25.0	0.9	
615-10-b_39	84.1	1.07	1.973	0.04	0.1888	0.0037	0.40	1103	15	1114	20	1079	47	1079.0	47.0	3.2	
615-10-b_40	71.5	1.57	2.189	0.07	0.2013	0.0057	0.56	1171	22	1180	31	1153	58	1153.0	58.0	2.3	
615-10-b_41	10.5	1.39	2.260	0.14	0.2069	0.0070	0.17	1183	43	1211	38	1120	140	1120.0	140.0	8.1	
615-10-b_42	290.5	7.13	2.209	0.05	0.1941	0.0039	0.62	1180	16	1142	21	1242	38	1242.0	38.0	8.1	
615-10-b_43	89.9	1.45	14.040	0.21	0.5527	0.0080	0.54	2751	14	2835	33	2682	23	2682.0	23.0	5.7	
615-10-b_44	101.1	2.19	14.020	0.15	0.5501	0.0061	0.67	2750	10	2824	25	2687	15	2687.0	15.0	5.1	
615-10-b_45	237.0	2.29	0.519	0.01	0.0672	0.0009	0.41	424	8	419	6	422	47	419.2	5.5	1.0	
615-10-b_46	198.7	3.02	15.960	0.42	0.5390	0.0200	0.63	2870	25	2771	84	2940	49	2940.0	49.0	5.7	
615-10-b_47	164.0	1.37	0.085	0.01	0.0124	0.0003	0.17	83	6	79	2	150	130	79.4	2.0	4.0	
615-10-b_48	275.0	2.27	0.892	0.02	0.1069	0.0013	0.31	647	8	654	8	612	39	654.3	7.5	1.2	
615-10-b_49	322.0	2.69	3.301	0.05	0.2613	0.0043	0.65	1479	13	1496	22	1454	26	1454.0	26.0	2.9	
615-10-b_50	39.5	1.62	5.340	0.14	0.3470	0.0067	0.53	1869	23	1919	32	1809	42	1809.0	42.0	6.1	
615-10-1	1320.0	4.75	1.289	0.08	0.1113	0.0032	0.70	839	36	680	19	1286	75	DISC	DISC	19.0	
615-10-1	593.0	1.62	3.989	0.03	0.2902	0.0020	0.55	1631	6	1642	10	1621	13	1621.0	13.0	1.3	Core
615-10-2	23.6	1.23	1.800	0.11	0.1781	0.0084	0.68	1045	40	1054	46	1080	90	1080.0	90.0	2.4	
615-10-3	198.6	0.69	12.800	0.13	0.5157	0.0056	0.65	2665	10	2680	24	2649	14	2649.0	14.0	1.2	
615-10-4	671.0	1.44	2.407	0.03	0.2066	0.0021	0.53	1244	9	1212	11	1304	24	1304.0	24.0	7.1	
615-10-5	206.8	1.56	3.990	0.15	0.2691	0.0085	0.44	1632	31	1534	43	1724	66	1724.0	66.0	11.0	
615-10-6	294.9	0.99	1.929	0.02	0.1847	0.0013	0.25	1092	7	1092	7	1088	23	1088.0	23.0	0.4	
615-10-8	215.0	0.48	0.162	0.01	0.0228	0.0003	0.15	152	5	145	2	251	79	145.1	2.1	4.5	
615-10-9	379.0	2.06	0.520	0.01	0.0676	0.0007	0.21	425	6	422	4	434	41	421.5	4.1	0.8	
615-10-11	64.1	0.71	2.122	0.06	0.1891	0.0033	0.48	1160	17	1116	18	1206	46	1206.0	46.0	7.5	
615-10-12	669.0	2.36	4.132	0.03	0.2898	0.0021	0.59	1660	6	1640	11	1686	11	1686.0	11.0	2.7	
615-10-13	459.0	2.53	3.096	0.03	0.2497	0.0019	0.49	1431	8	1437	10	1430	18	1430.0	18.0	0.5	
615-10-15	249.0	2.20	3.034	0.07	0.2440	0.0055	0.54	1419	19	1407	29	1436	51	1436.0	51.0	2.0	
615-10-16	980.0	2.82	0.059	0.00	0.0089	0.0002	0.18	58	3	57	1	68	88	56.9	1.0	1.7	Rim
615-10-16	128.9	1.50	0.180	0.02	0.0262	0.0009	0.17	167	13	167	6	120	130	166.8	5.5	0.1	Core

	[U] ppm	U/Th	$^{207}Pb/^{235}U$	2σ error	$^{206}Pb/^{238}U$	2σ error	RHO	$^{207}Pb/^{235}U$ Age Ma	2σ error	$^{206}Pb/^{238}U$ Age (Ma)	2σ error	$^{207}Pb/^{206}Pb$ Age (Ma)	2σ error	Best age (Ma)	2σ error	% Disc*	Rim or core
615-10-17	506.0	1.25	0.075	0.00	0.0113	0.0002	0.10	74	3	73	1	108	85	72.6	1.3	1.2	
615-10-18	214.8	1.92	0.216	0.01	0.0302	0.0005	0.20	198	7	192	3	261	85	192.0	3.0	2.8	
615-10-19	1236.0	3.05	0.074	0.00	0.0106	0.0001	0.21	72	2	68	1	222	62	67.7	0.8	6.4	
615-10-20	62.9	0.09	0.622	0.02	0.0102	0.0004	0.08	491	15	65	3	4112	80	DISC	DISC	86.7	
615-10-22	596.0	0.45	0.429	0.01	0.0370	0.0006	0.51	363	8	234	4	1301	42	DISC	DISC	35.6	
615-10-23	1980.0	0.79	0.925	0.01	0.1077	0.0010	0.62	665	5	659	6	687	21	659.1	5.6	0.9	
615-10-24	28.5	1.03	2.740	0.14	0.2396	0.0082	0.69	1326	38	1382	43	1273	79	1273.0	79.0	8.6	
615-10-25	225.2	1.89	3.350	0.14	0.2259	0.0097	0.70	1490	34	1309	51	1742	60	1742.0	60.0	24.9	
615-10-26	91.6	0.41	5.431	0.08	0.3366	0.0039	0.56	1889	13	1870	19	1911	22	1911.0	22.0	2.1	
615-10-27	96.5	0.38	13.260	0.17	0.5108	0.0057	0.69	2697	13	2659	24	2718	15	2718.0	15.0	2.2	
615-10-28	133.2	0.92	3.142	0.07	0.2439	0.0033	0.49	1441	18	1407	17	1488	38	1488.0	38.0	5.4	
615-10-29	215.5	5.97	11.170	0.15	0.4426	0.0067	0.65	2538	13	2361	30	2676	23	2676.0	23.0	11.8	
615-10-30	169.0	1.82	0.636	0.03	0.0796	0.0017	0.21	498	18	494	10	513	94	494.0	10.0	0.8	Rim
615-10-30	56.4	0.67	1.562	0.09	0.1595	0.0043	0.28	955	33	954	24	960	100	960.0	100.0	0.6	Core
615-10-31	481.0	0.55	0.425	0.01	0.0574	0.0006	0.30	359	6	360	4	338	44	359.8	3.6	0.2	
615-10-33	140.9	0.76	13.280	0.19	0.4628	0.0065	0.77	2702	14	2451	29	2894	16	2894.0	16.0	15.3	Rim
615-10-33	151.9	0.95	15.770	0.23	0.5375	0.0077	0.72	2862	14	2772	32	2908	19	2908.0	19.0	4.7	Core
615-10-34	73.0	0.93	0.550	0.02	0.0695	0.0013	0.21	443	14	433	8	491	85	432.9	8.0	2.3	
615-10-36	149.9	1.02	0.178	0.01	0.0255	0.0004	0.03	166	7	162	3	219	93	162.3	2.7	2.3	
615-10-37	282.8	1.12	4.508	0.10	0.1606	0.0037	0.80	1733	19	960	20	2862	23	DISC	DISC	66.5	
615-10-38	160.0	0.98	4.931	0.08	0.3364	0.0061	0.53	1806	14	1868	29	1745	30	1745.0	30.0	7.0	
615-10-40	627.0	1.83	2.242	0.02	0.2057	0.0015	0.52	1194	7	1206	8	1177	18	1177.0	18.0	2.4	
615-10-41	57.6	0.46	4.810	0.10	0.3018	0.0043	0.38	1785	18	1699	21	1900	36	1900.0	36.0	10.6	
615-10-42	257.7	0.79	1.955	0.03	0.1776	0.0015	0.35	1099	9	1054	8	1201	25	1201.0	25.0	12.3	
615-10-43	205.0	2.36	0.079	0.00	0.0127	0.0002	0.08	77	4	82	1	-10	110	81.5	1.3	5.6	
615-10-44	459.0	1.30	0.072	0.00	0.0113	0.0002	0.10	71	3	73	1	55	83	72.7	1.2	2.1	
615-10-46	143.4	1.30	13.470	0.25	0.5356	0.0086	0.55	2712	18	2764	36	2677	25	2677.0	25.0	3.2	
615-10-47	354.1	0.73	4.858	0.06	0.3003	0.0022	0.27	1793	11	1692	11	1911	23	1911.0	23.0	11.5	
615-10-48	42.0	0.59	1.492	0.06	0.1563	0.0040	0.55	927	26	935	22	909	75	909.0	75.0	2.9	
615-10-49	1257.0	1.40	3.359	0.04	0.1254	0.0014	0.46	1494	9	762	8	2778	16	DISC	DISC	49.0	
615-10-51	182.0	1.14	2.236	0.03	0.2047	0.0018	0.33	1191	10	1201	10	1182	26	1182.0	26.0	1.6	
615-10-52	216.4	1.97	0.255	0.01	0.0367	0.0004	0.06	230	6	232	2	211	64	232.3	2.4	0.9	
615-10-53	91.0	1.08	3.087	0.07	0.2440	0.0030	0.53	1428	17	1412	16	1450	32	1450.0	32.0	2.6	
615-10-55	140.0	2.89	14.380	0.19	0.5327	0.0060	0.76	2774	13	2752	25	2798	15	2798.0	15.0	1.6	
615-10-56	68.7	0.50	2.884	0.09	0.2189	0.0047	0.62	1378	22	1275	25	1560	43	1560.0	43.0	18.3	

	[U] ppm	U/Th	$^{207}Pb/^{235}U$	2σ error	$^{206}Pb/^{238}U$	2σ error	RHO	$^{207}Pb/^{235}U$ Age Ma	2σ error	$^{206}Pb/^{238}U$ Age (Ma)	2σ error	$^{207}Pb/^{206}Pb$ Age (Ma)	2σ error	Best age (Ma)	2σ error	% Disc*	Rim or core
615-10-57	99.5	1.54	3.075	0.05	0.2468	0.0029	0.49	1428	13	1421	15	1426	26	1426.0	26.0	0.4	
615-10-58	108.1	0.51	3.653	0.06	0.2621	0.0030	0.46	1562	12	1502	16	1640	28	1640.0	28.0	8.4	
615-10-59	316.0	0.69	0.067	0.00	0.0103	0.0002	0.09	66	3	66	1	98	89	66.0	1.2	0.2	
615-10-60	88.9	0.78	2.542	0.05	0.2217	0.0030	0.36	1282	13	1290	16	1262	37	1262.0	37.0	2.2	
615-10-61	891.0	1.77	1.883	0.02	0.1367	0.0013	0.54	1074	8	826	7	1597	22	DISC	DISC	23.1	
615-10-63	721.0	1.18	0.099	0.00	0.0152	0.0002	0.32	96	3	97	1	71	55	97.3	1.2	1.6	
615-10-64	226.7	0.86	0.512	0.01	0.0660	0.0007	0.31	419	7	412	4	453	47	412.2	4.1	1.7	
615-10-65	103.4	0.50	5.880	0.10	0.3605	0.0063	0.58	1959	15	1983	30	1935	30	1935.0	30.0	2.5	
615-10-66	150.3	1.34	1.716	0.04	0.1656	0.0019	0.30	1014	14	989	11	1069	46	1069.0	46.0	7.5	
615-10-67	126.8	0.39	0.033	0.01	0.0039	0.0002	0.22	33	6	25	2	390	350	DISC	DISC	23.6	
615-10-68	70.9	1.70	1.990	0.04	0.1902	0.0024	0.43	1112	15	1122	13	1094	38	1094.0	38.0	2.6	
615-10-69	101.1	1.71	2.748	0.05	0.2293	0.0026	0.19	1342	14	1330	14	1353	40	1353.0	40.0	1.7	
615-10-70	135.5	0.50	3.058	0.04	0.2470	0.0025	0.45	1423	9	1424	13	1423	24	1423.0	24.0	0.1	
615-10-71	219.0	2.31	2.086	0.03	0.1961	0.0017	0.36	1144	9	1154	9	1128	25	1128.0	25.0	2.3	
615-10-72	477.0	0.79	1.940	0.05	0.0821	0.0010	0.08	1095	17	508	6	2569	46	DISC	DISC	53.6	
615-10-73	124.7	1.94	2.308	0.05	0.2049	0.0026	0.39	1217	14	1202	14	1232	38	1232.0	38.0	2.4	
615-10-74	40.1	1.09	1.695	0.06	0.1708	0.0042	0.59	1000	22	1016	23	987	65	987.0	65.0	2.9	
615-10-75	111.2	0.47	2.775	0.07	0.2320	0.0051	0.58	1346	20	1344	26	1358	43	1358.0	43.0	1.0	
615-10-77	154.9	1.19	2.000	0.10	0.1593	0.0017	0.11	1118	34	953	10	1428	93	DISC	DISC	33.3	
615-10-78	329.0	2.02	1.864	0.02	0.1786	0.0014	0.39	1068	6	1059	8	1078	21	1078.0	21.0	1.8	
615-10-79	180.9	1.22	1.981	0.04	0.1860	0.0017	0.40	1108	12	1101	9	1126	31	1126.0	31.0	2.2	
615-10-80	278.0	1.10	0.503	0.01	0.0661	0.0008	0.27	413	7	413	5	417	45	412.5	4.7	0.2	
615-10-81	165.0	0.63	2.841	0.04	0.2382	0.0026	0.26	1367	11	1377	13	1350	29	1350.0	29.0	2.0	
615-10-82	268.0	1.09	1.762	0.05	0.1719	0.0043	0.32	1030	19	1022	24	1044	70	1044.0	70.0	2.1	Rim
615-10-82	131.5	1.71	2.752	0.07	0.2320	0.0039	0.54	1344	18	1345	20	1341	37	1341.0	37.0	0.3	Core
615-10-83	385.0	4.42	3.870	0.23	0.1416	0.0062	0.88	1603	46	853	35	2825	43	DISC	DISC	69.8	Rim
615-10-83	54.9	1.63	16.010	0.42	0.5580	0.0150	0.84	2872	25	2861	62	2884	23	2884.0	23.0	0.8	Core
615-10-84	46.0	0.57	1.474	0.06	0.1505	0.0040	0.51	925	22	905	22	930	70	930.0	70.0	2.7	
615-10-85	190.7	1.66	0.729	0.02	0.0879	0.0012	0.32	556	10	543	7	635	49	542.9	7.4	2.3	
615-10-86	443.9	48.00	1.941	0.08	0.1784	0.0048	0.75	1094	29	1058	26	1196	61	1196.0	61.0	11.5	Rim
615-10-86	119.8	1.76	2.897	0.04	0.2357	0.0023	0.28	1381	11	1364	12	1417	27	1417.0	27.0	3.7	Core
615-10-87	1036.0	1.22	2.129	0.02	0.1865	0.0013	0.62	1159	7	1102	7	1259	15	1259.0	15.0	12.5	
615-10-88	287.4	1.62	1.878	0.05	0.1800	0.0029	0.46	1072	17	1067	16	1090	45	1090.0	45.0	2.1	
615-10-89	71.2	1.72	1.229	0.05	0.0856	0.0025	0.12	810	23	532	14	1741	95	DISC	DISC	34.3	
615-10-90	88.0	1.06	1.779	0.04	0.1742	0.0033	0.31	1040	16	1034	18	1004	50	1004.0	50.0	3.0	

	[U] ppm	U/Th	$^{207}Pb/^{235}U$	2σ error	$^{206}Pb/^{238}U$	2σ error	RHO	$^{207}Pb/^{235}U$ Age Ma	2σ error	$^{206}Pb/^{238}U$ Age (Ma)	2σ error	$^{207}Pb/^{206}Pb$ Age (Ma)	2σ error	Best age (Ma)	2σ error	% Disc*	Rim or core
615-10-91	504.0	0.93	3.956	0.04	0.2687	0.0018	0.54	1627	7	1534	9	1748	15	1748.0	15.0	12.2	
615-10-93	266.7	2.55	4.367	0.04	0.2947	0.0026	0.44	1705	8	1665	13	1754	18	1754.0	18.0	5.1	
615-10-94	129.1	1.13	0.072	0.01	0.0106	0.0002	0.03	68	5	68	2	90	140	68.1	1.5	0.3	
615-10-95	63.8	0.76	1.767	0.05	0.1662	0.0029	0.31	1034	18	994	16	1103	55	1103.0	55.0	9.9	
615-10-96	308.9	1.58	2.655	0.08	0.2235	0.0055	0.74	1319	20	1299	29	1383	37	1383.0	37.0	6.1	
615-10-97	279.6	1.15	2.018	0.06	0.1661	0.0018	0.10	1118	18	991	10	1384	44	1384.0	44.0	28.4	
615-10-98	25.3	0.48	13.240	0.55	0.5130	0.0200	0.88	2703	37	2661	83	2719	34	2719.0	34.0	2.1	
615-10-99	141.7	1.15	1.991	0.03	0.1828	0.0018	0.32	1112	10	1082	10	1168	29	1168.0	29.0	7.4	
615-10-100	51.0	1.55	2.905	0.09	0.2348	0.0037	0.31	1375	23	1359	19	1410	46	1410.0	46.0	3.6	
615-10-101	206.9	0.66	4.746	0.06	0.2940	0.0028	0.54	1774	11	1663	14	1908	20	1908.0	20.0	12.8	
615-10-102	404.0	1.18	0.076	0.00	0.0115	0.0002	0.16	75	3	74	1	101	94	73.7	1.2	1.1	
615-10-103	196.9	0.49	12.170	0.14	0.4866	0.0046	0.70	2617	11	2556	20	2663	14	2663.0	14.0	4.0	
615-10-104	825.0	2.81	1.752	0.02	0.1748	0.0013	0.40	1028	6	1038	7	1011	19	1011.0	19.0	2.7	
615-10-105	112.6	0.96	4.305	0.06	0.2942	0.0033	0.51	1694	11	1662	16	1746	24	1746.0	24.0	4.8	
615-10-106	95.5	1.67	0.224	0.01	0.0327	0.0006	0.20	206	9	208	4	162	92	207.5	3.5	0.9	
615-10-107	369.0	2.23	2.929	0.04	0.2329	0.0031	0.61	1390	10	1349	16	1463	22	1463.0	22.0	7.8	
615-10-108	353.1	1.61	1.999	0.04	0.1792	0.0039	0.68	1114	12	1062	21	1205	32	1205.0	32.0	11.9	
615-10-109	97.4	1.02	2.750	0.08	0.2221	0.0060	0.62	1337	23	1299	33	1399	45	1399.0	45.0	7.1	
615-10-110	111.1	1.15	12.020	0.23	0.4820	0.0100	0.70	2603	18	2534	45	2648	26	2648.0	26.0	4.3	
615-10-111	384.0	1.44	2.929	0.09	0.2205	0.0063	0.57	1392	24	1283	33	1556	54	1556.0	54.0	17.5	
615-10-112	101.2	10.80	1.647	0.08	0.1725	0.0053	0.09	985	29	1025	29	910	120	910.0	120.0	12.6	Rim
615-10-112	68.1	0.72	3.476	0.07	0.2612	0.0039	0.37	1521	17	1495	20	1576	40	1576.0	40.0	5.1	Core
615-10-113	95.2	1.28	2.091	0.06	0.1910	0.0040	0.48	1144	20	1129	22	1188	54	1188.0	54.0	5.0	
615-10-114	232.0	1.39	2.843	0.03	0.2372	0.0018	0.53	1367	8	1372	10	1367	19	1367.0	19.0	0.4	
615-10-115	80.1	0.74	2.652	0.05	0.2254	0.0030	0.58	1317	14	1310	16	1331	30	1331.0	30.0	1.6	
615-10-116	392.0	1.58	0.205	0.01	0.0294	0.0003	0.05	189	4	187	2	224	57	186.6	2.0	1.2	
615-10-117	47.2	0.48	3.060	0.10	0.2382	0.0080	0.56	1423	27	1375	42	1512	57	1512.0	57.0	9.1	
615-10-118	109.7	0.93	2.399	0.05	0.2126	0.0036	0.42	1241	16	1242	19	1241	41	1241.0	41.0	0.1	
615-10-119	211.2	1.29	0.059	0.00	0.0098	0.0003	0.11	58	4	63	2	-20	120	62.7	1.6	7.4	
615-10-120	160.9	1.94	0.251	0.01	0.0362	0.0006	0.04	228	8	229	4	233	85	229.1	3.8	0.6	

U-Pb isotopic data for sample 615-11

	[U] ppm	U/Th	^{207}Pb/^{235}U	2σ error	^{206}Pb/^{238}U	2σ error	RHO	^{207}Pb/^{235}U Age Ma	2σ error	^{206}Pb/^{238}U Age (Ma)	2σ error	^{207}Pb/^{206}Pb Age (Ma)	2σ error	Best age (Ma)	2σ error	% Disc*	Rim or core
615-11-b_1	112.2	1.83	0.081	0.01	0.0114	0.0004	0.01	79	7	73	3	220	170	73.3	2.5	6.6	
615-11-b_2	731.0	29.10	1.992	0.05	0.1914	0.0041	0.60	1111	16	1128	22	1070	41	1070.0	41.0	5.4	
615-11-b_3	63.5	1.47	1.767	0.05	0.1765	0.0046	0.39	1037	17	1046	25	1015	57	1015.0	57.0	3.1	
615-11-b_4	535.0	1.23	0.029	0.00	0.0044	0.0001	0.11	29	2	28	1	100	120	28.2	0.7	1.4	
615-11-b_5	409.0	6.64	1.646	0.06	0.1673	0.0073	0.75	987	22	997	40	970	56	970.0	56.0	2.8	
615-11-b_5	112.0	2.50	2.229	0.05	0.2047	0.0036	0.62	1187	16	1200	19	1167	38	1167.0	38.0	2.8	
615-11-b_6	74.7	1.79	2.029	0.05	0.1962	0.0037	0.43	1121	16	1154	20	1072	49	1072.0	49.0	7.6	
615-11-b_7	64.3	0.77	4.970	0.18	0.3330	0.0140	0.55	1804	30	1845	66	1797	65	1797.0	65.0	2.7	
615-11-b_8	128.9	2.73	5.125	0.09	0.3409	0.0067	0.56	1837	15	1889	32	1781	32	1781.0	32.0	6.1	
615-11-b_9	80.4	1.62	0.027	0.01	0.0048	0.0003	0.06	27	6	31	2	-140	350	DISC	DISC	13.8	
615-11-b_10	105.3	31.90	1.894	0.04	0.1808	0.0024	0.24	1076	13	1071	13	1081	43	1081.0	43.0	0.9	
615-11-b_11	220.0	1.36	4.120	0.16	0.2810	0.0140	0.65	1645	34	1585	68	1740	68	1740.0	68.0	8.9	
615-11-b_12	122.0	3.38	3.060	0.10	0.2481	0.0080	0.53	1418	24	1425	41	1417	57	1417.0	57.0	0.6	
615-11-b_13	81.7	0.94	1.999	0.05	0.1944	0.0040	0.29	1111	19	1144	22	1044	62	1044.0	62.0	9.6	
615-11-b_14	115.2	1.26	1.991	0.04	0.1898	0.0027	0.36	1110	14	1120	15	1099	42	1099.0	42.0	1.9	
615-11-b_15	78.1	1.17	2.376	0.06	0.2193	0.0039	0.33	1232	17	1277	21	1158	46	1158.0	46.0	10.3	
615-11-b_16	176.6	3.17	0.234	0.01	0.0331	0.0014	0.48	212	10	211	8	272	90	210.8	8.4	0.6	
615-11-b_17	60.1	0.93	13.010	0.31	0.4970	0.0120	0.55	2680	21	2595	51	2743	37	2743.0	37.0	5.4	
615-11-b_18	94.0	1.97	3.852	0.09	0.2821	0.0066	0.35	1598	19	1599	33	1612	46	1612.0	46.0	0.8	
615-11-b_19	175.3	1.58	4.311	0.08	0.3027	0.0052	0.54	1693	14	1704	26	1678	31	1678.0	31.0	1.5	
615-11-b_20	34.7	23.10	11.110	0.55	0.4670	0.0250	0.59	2520	49	2470	110	2587	81	2587.0	81.0	4.5	
615-11-b_21	93.0	2.49	0.229	0.01	0.0317	0.0008	0.12	207	11	201	5	260	110	201.2	4.8	2.8	
615-11-b_22	38.8	2.46	2.692	0.09	0.2127	0.0056	0.24	1321	26	1241	30	1447	77	1447.0	77.0	14.2	
615-11-b_23	22.2	3.79	3.200	0.12	0.2012	0.0074	0.34	1449	30	1185	41	1848	81			35.9	
615-11-b_24	140.0	0.83	3.680	0.10	0.2771	0.0074	0.62	1560	23	1573	37	1525	46	1525.0	46.0	3.1	
615-11-b_25	121.7	1.80	2.886	0.06	0.2341	0.0043	0.43	1375	16	1355	22	1378	41	1378.0	41.0	1.7	
615-11-b_26	293.0	8.20	9.020	0.40	0.3930	0.0180	0.68	2329	43	2125	86	2520	61	2520.0	61.0	15.7	
615-11-b_27	285.0	1.27	4.420	0.10	0.3015	0.0071	0.63	1716	18	1697	35	1716	37	1716.0	37.0	1.1	
615-11-b_28	83.9	6.50	5.940	0.26	0.3670	0.0180	0.54	1946	38	2011	88	1890	79	1890.0	79.0	6.4	
615-11-b_29	311.0	5.87	1.980	0.10	0.1870	0.0100	0.57	1101	36	1099	56	1110	100	1110.0	100.0	1.0	
615-11-b_30	161.0	36.20	1.836	0.10	0.1730	0.0080	0.70	1042	34	1031	45	1043	81	1043.0	81.0	1.2	
615-11-b_31	23.4	1.45	18.630	0.33	0.6153	0.0097	0.44	3018	17	3098	39	2951	29	2951.0	29.0	5.0	
615-11-b_32	158.0	13.00	2.723	0.08	0.2336	0.0057	0.52	1327	22	1351	30	1262	55	1262.0	55.0	7.1	

	[U] ppm	U/Th	$^{207}Pb/^{235}U$	2σ error	$^{206}Pb/^{238}U$	2σ error	RHO	$^{207}Pb/^{235}U$ Age Ma	2σ error	$^{206}Pb/^{238}U$ Age (Ma)	2σ error	$^{207}Pb/^{206}Pb$ Age (Ma)	2σ error	Best age (Ma)	2σ error	% Disc*	Rim or core
615-11-b_33	156.6	2.59	1.950	0.06	0.1873	0.0045	0.21	1093	20	1106	24	1041	43	1041.0	43.0	6.2	
615-11-b_34	100.0	3.71	2.095	0.09	0.1918	0.0073	0.65	1136	30	1132	41	1126	71	1126.0	71.0	0.5	
615-11-b_35	75.0	2.41	2.002	0.06	0.1945	0.0049	0.50	1109	21	1144	26	1042	57	1042.0	57.0	9.8	
615-11-b_36	31.5	3.26	4.480	0.15	0.3163	0.0095	0.49	1719	29	1768	47	1647	61	1647.0	61.0	7.3	
615-11-b_37	55.7	1.11	0.031	0.01	0.0047	0.0003	0.02	30	9	31	2	-220	440	30.5	1.9	1.0	
615-11-b_38	168.0	2.38	3.710	0.21	0.2550	0.0150	0.57	1547	44	1450	75	1666	94	1666.0	94.0	13.0	
615-11-b_39	172.0	4.90	3.240	0.13	0.2650	0.0120	0.65	1457	30	1513	62	1399	62	1399.0	62.0	8.1	
615-11-b_40	128.0	0.77	0.233	0.02	0.0257	0.0011	0.33	214	13	163	7	730	130			23.6	
615-11-b_41	99.1	1.58	0.036	0.01	0.0037	0.0002	0.22	35	6	24	2	480	330			32.7	
615-11-b_42	68.5	1.13	13.980	0.33	0.5610	0.0140	0.63	2742	22	2865	59	2645	33	2645.0	33.0	8.3	
615-11-b_43	289.0	1.69	0.125	0.01	0.0179	0.0004	0.07	120	5	114	2	200	100	114.3	2.4	4.4	
615-11-b_44	213.0	2.90	2.159	0.05	0.2053	0.0044	0.63	1164	17	1203	23	1072	40	1072.0	40.0	12.2	
615-11-b_45	36.8	0.99	6.310	0.20	0.3830	0.0100	0.57	2013	26	2086	49	1912	45	1912.0	45.0	9.1	
615-11-b_46	1120.0	12.74	4.502	0.07	0.3059	0.0059	0.62	1731	13	1719	29	1733	26	1733.0	26.0	0.8	
615-11-b_47	216.0	1.42	1.759	0.04	0.1499	0.0038	0.50	1026	16	899	21	1290	49			30.3	
615-11-b_48	113.0	1.87	7.720	0.20	0.4160	0.0110	0.51	2193	23	2234	51	2168	46	2168.0	46.0	3.0	
615-11-b_49	83.7	4.06	2.528	0.07	0.2322	0.0061	0.55	1273	21	1344	32	1158	53	1158.0	53.0	16.1	
615-11-b_50	126.7	3.15	2.590	0.11	0.2260	0.0091	0.51	1281	32	1308	47	1226	79	1226.0	79.0	6.7	
615-11-1	111.0	1.01	5.833	0.08	0.3574	0.0045	0.59	1952	12	1972	21	1940	22	1940.0	22.0	1.6	
615-11-2	193.9	1.08	4.194	0.04	0.2940	0.0022	0.49	1673	8	1661	11	1691	17	1691.0	17.0	1.8	
615-11-3	137.2	1.24	2.884	0.04	0.2444	0.0020	0.40	1376	11	1409	11	1335	25	1335.0	25.0	5.5	
615-11-4	63.3	0.51	2.660	0.06	0.2231	0.0042	0.48	1314	17	1300	22	1353	44	1353.0	44.0	3.9	
615-11-5	63.3	1.27	3.269	0.06	0.2543	0.0043	0.48	1471	15	1460	22	1494	35	1494.0	35.0	2.3	
615-11-7	126.6	0.83	3.288	0.05	0.2560	0.0026	0.52	1477	12	1469	13	1489	23	1489.0	23.0	1.3	
615-11-8	103.8	0.63	13.820	0.15	0.5458	0.0055	0.64	2736	10	2809	23	2682	16	2682.0	16.0	4.7	
615-11-9	235.0	5.40	13.000	0.10	0.5169	0.0049	0.63	2681	7	2685	21	2676	13	2676.0	13.0	0.3	
615-11-10	325.0	0.22	0.827	0.01	0.0991	0.0007	0.24	612	7	609	4	628	34	609.0	4.3	0.5	
615-11-11	116.8	0.92	3.303	0.06	0.2626	0.0031	0.45	1483	15	1503	16	1451	36	1451.0	36.0	3.6	
615-11-12	245.0	0.92	5.330	0.06	0.3355	0.0030	0.48	1873	10	1865	15	1883	18	1883.0	18.0	1.0	
615-11-13	429.0	1.08	0.201	0.00	0.0290	0.0003	0.22	186	4	184	2	189	51	184.2	1.7	0.8	
615-11-14	166.1	0.39	12.730	0.11	0.5120	0.0036	0.57	2659	8	2665	15	2656	12	2656.0	12.0	0.3	
615-11-15	79.7	0.93	1.930	0.04	0.1863	0.0025	0.47	1090	14	1101	13	1043	41	1043.0	41.0	5.6	
615-11-16	75.0	1.04	2.704	0.05	0.2325	0.0037	0.38	1328	14	1347	19	1305	40	1305.0	40.0	3.2	
615-11-17	313.0	2.07	2.040	0.02	0.1883	0.0017	0.33	1129	8	1112	9	1161	25	1161.0	25.0	4.2	

	[U] ppm	U/Th	$^{207}Pb/^{235}U$	2σ error	$^{206}Pb/^{238}U$	2σ error	RHO	$^{207}Pb/^{235}U$ Age Ma	2σ error	$^{206}Pb/^{238}U$ Age (Ma)	2σ error	$^{207}Pb/^{206}Pb$ Age (Ma)	2σ error	Best age (Ma)	2σ error	% Disc*	Rim or core
615-11-18	201.6	1.32	1.909	0.03	0.1850	0.0019	0.38	1083	9	1094	11	1057	25	1057.0	25.0	3.5	
615-11-19	166.8	0.71	4.924	0.07	0.3149	0.0037	0.50	1805	11	1764	18	1847	24	1847.0	24.0	4.5	
615-11-20	73.3	0.66	4.938	0.08	0.3167	0.0040	0.59	1808	14	1773	20	1846	26	1846.0	26.0	4.0	
615-11-21	276.0	2.00	1.769	0.02	0.1723	0.0013	0.28	1034	8	1025	7	1058	25	1058.0	25.0	3.1	
615-11-22	809.0	33.50	0.554	0.02	0.0710	0.0008	0.36	447	13	442	5	478	66	442.2	4.6	1.1	Rim
615-11-22	228.0	1.22	4.082	0.08	0.2807	0.0042	0.46	1649	16	1595	21	1706	33	1706.0	33.0	6.5	Core
615-11-23	112.4	1.28	1.869	0.04	0.1785	0.0026	0.55	1072	15	1058	14	1088	39	1088.0	39.0	2.8	
615-11-24	313.0	2.56	1.833	0.02	0.1747	0.0015	0.33	1057	8	1038	8	1109	25	1109.0	25.0	6.4	
615-11-25	351.0	0.89	0.048	0.00	0.0047	0.0001	0.13	48	4	30	1	920	170			36.7	
615-11-26	127.7	1.34	3.399	0.07	0.2622	0.0026	0.54	1500	15	1500	13	1506	33	1506.0	33.0	0.4	
615-11-27	278.0	1.47	1.797	0.02	0.1764	0.0012	0.20	1045	8	1047	7	1031	25	1031.0	25.0	1.6	
615-11-28	131.5	1.47	1.762	0.03	0.1691	0.0017	0.48	1031	11	1007	9	1085	31	1085.0	31.0	7.2	
615-11-29	161.9	1.50	2.927	0.04	0.2377	0.0022	0.35	1391	10	1376	11	1408	25	1408.0	25.0	2.3	
615-11-31	288.0	0.83	0.169	0.01	0.0254	0.0003	0.06	159	5	162	2	128	69	161.6	2.0	1.6	
615-11-32	306.2	1.16	3.097	0.06	0.2446	0.0029	0.59	1434	17	1411	15	1467	37	1467.0	37.0	3.8	
615-11-33	59.3	1.09	1.920	0.05	0.1881	0.0028	0.41	1091	17	1112	15	1051	51	1051.0	51.0	5.8	
615-11-34	924.0	2.51	10.850	0.16	0.4547	0.0044	0.46	2513	14	2416	20	2581	20	2581.0	20.0	6.4	
615-11-36	130.3	0.61	0.062	0.01	0.0064	0.0002	0.07	59	7	41	2	720	220			31.0	
615-11-37	429.0	2.60	2.524	0.04	0.2187	0.0028	0.55	1279	12	1275	15	1277	27	1277.0	27.0	0.2	
615-11-38	121.3	1.25	3.121	0.04	0.2529	0.0026	0.45	1436	10	1453	14	1412	26	1412.0	26.0	2.9	
615-11-39	3179.0	28.80	1.918	0.03	0.1842	0.0024	0.60	1087	11	1090	13	1086	24	1086.0	24.0	0.4	Rim
615-11-39	1492.0	12.23	3.197	0.03	0.2534	0.0021	0.57	1456	7	1456	11	1454	15	1454.0	15.0	0.1	Core
615-11-40	309.0	1.49	3.670	0.12	0.2630	0.0079	0.33	1563	27	1505	40	1636	72	1636.0	72.0	8.0	Rim
615-11-40	108.7	0.87	4.865	0.07	0.3280	0.0033	0.60	1794	12	1832	16	1745	22	1745.0	22.0	5.0	Core
615-11-41	148.0	1.42	0.065	0.00	0.0095	0.0002	0.09	64	5	61	1	220	130	61.0	1.3	4.4	
615-11-42	124.6	0.90	1.590	0.03	0.1632	0.0019	0.35	965	12	974	10	946	36	946.0	36.0	3.0	
615-11-43	140.5	0.89	2.465	0.04	0.2097	0.0024	0.21	1260	12	1227	13	1307	35	1307.0	35.0	6.1	
615-11-44	535.0	1.85	4.761	0.05	0.3168	0.0031	0.50	1779	9	1775	15	1769	18	1769.0	18.0	0.3	
615-11-45	1841.0	9.55	2.262	0.02	0.2059	0.0015	0.39	1200	6	1207	8	1191	14	1191.0	14.0	1.3	
615-11-46	952.0	3.28	2.419	0.02	0.2159	0.0014	0.50	1248	6	1260	7	1218	14	1218.0	14.0	3.5	
615-11-47	307.0	0.90	3.122	0.05	0.2489	0.0031	0.63	1437	12	1432	16	1445	25	1445.0	25.0	0.9	
615-11-48	79.7	1.10	1.820	0.05	0.1769	0.0028	0.41	1057	17	1050	15	1073	47	1073.0	47.0	2.1	
615-11-50	275.3	2.07	0.243	0.01	0.0323	0.0003	0.16	221	6	205	2	384	69	204.8	2.1	7.4	
615-11-51	139.6	4.10	1.822	0.04	0.1745	0.0021	0.40	1056	15	1037	12	1099	36	1099.0	36.0	5.6	
615-11-52	190.0	1.79	3.558	0.05	0.2725	0.0026	0.40	1544	10	1553	13	1519	24	1519.0	24.0	2.2	

	[U] ppm	U/Th	^{207}Pb/ ^{235}U	2σ error	^{206}Pb/ ^{238}U	2σ error	RHO	^{207}Pb/^{235}U Age Ma	2σ error	^{206}Pb/^{238}U Age (Ma)	2σ error	^{207}Pb/^{206}Pb Age (Ma)	2σ error	Best age (Ma)	2σ error	% Disc*	Rim or core
615-11-53	47.5	2.31	2.218	0.06	0.1987	0.0035	0.49	1186	20	1170	19	1190	50	1190.0	50.0	1.7	
615-11-54	22.4	2.23	14.410	0.71	0.5500	0.0270	0.89	2772	49	2810	110	2749	36	2749.0	36.0	2.2	
615-11-55	103.3	0.97	1.926	0.04	0.1852	0.0021	0.43	1094	13	1095	12	1074	35	1074.0	35.0	2.0	
615-11-57	47.0	1.02	14.720	0.22	0.5576	0.0092	0.75	2796	14	2853	38	2743	19	2743.0	19.0	4.0	
615-11-58	46.7	1.23	2.307	0.06	0.1996	0.0042	0.58	1213	19	1174	22	1283	49	1283.0	49.0	8.5	
615-11-59	57.1	0.98	13.460	0.20	0.5291	0.0078	0.75	2713	14	2739	33	2684	16	2684.0	16.0	2.0	
615-11-60	807.0	0.94	0.056	0.00	0.0087	0.0001	0.15	56	2	56	1	62	74	56.0	0.8	0.5	
615-11-61	114.2	1.53	1.756	0.03	0.1688	0.0020	0.41	1027	12	1005	11	1069	35	1069.0	35.0	6.0	
615-11-62	562.0	0.92	0.100	0.00	0.0155	0.0002	0.03	97	3	99	1	55	62	99.0	1.1	2.1	
615-11-63	438.0	1.54	14.730	0.10	0.5461	0.0044	0.64	2798	7	2811	19	2779	11	2779.0	11.0	1.2	
615-11-64	170.0	2.43	1.067	0.06	0.0876	0.0011	0.47	724	31	542	7	1310	110			25.1	
615-11-65	58.9	1.07	3.307	0.08	0.2637	0.0049	0.54	1485	18	1510	25	1438	40	1438.0	40.0	5.0	
615-11-66	161.6	1.32	1.951	0.03	0.1836	0.0019	0.41	1097	10	1087	10	1114	31	1114.0	31.0	2.4	
615-11-67	140.8	0.83	1.789	0.03	0.1764	0.0017	0.55	1040	9	1047	9	1016	25	1016.0	25.0	3.1	
615-11-68	546.0	16.40	1.870	0.02	0.1817	0.0015	0.41	1071	9	1076	8	1059	25	1059.0	25.0	1.6	Rim
615-11-68	347.0	0.48	4.352	0.06	0.3080	0.0054	0.63	1703	11	1731	26	1672	26	1672.0	26.0	3.5	Core
615-11-69	184.7	2.50	1.929	0.05	0.1775	0.0052	0.50	1086	19	1052	28	1167	59	1167.0	59.0	9.9	
615-11-70	229.0	1.84	4.417	0.04	0.3062	0.0024	0.54	1714	8	1723	12	1702	18	1702.0	18.0	1.2	
615-11-71	46.4	1.39	11.160	0.24	0.4810	0.0100	0.79	2534	20	2533	44	2551	24	2551.0	24.0	0.7	
615-11-72	447.0	2.30	3.337	0.09	0.2373	0.0051	0.88	1487	22	1372	26	1669	24	1669.0	24.0	17.8	
615-11-73	105.0	0.75	14.200	0.21	0.5437	0.0078	0.77	2761	14	2797	33	2736	17	2736.0	17.0	2.2	
615-11-74	646.0	4.01	5.070	0.11	0.3052	0.0033	0.48	1829	18	1717	16	1964	34	1964.0	34.0	12.6	
615-11-75	242.4	1.25	3.309	0.04	0.2593	0.0022	0.52	1483	9	1486	11	1474	21	1474.0	21.0	0.8	
615-11-76	312.0	4.27	13.170	0.16	0.5165	0.0052	0.68	2691	12	2684	22	2692	15	2692.0	15.0	0.3	
615-11-77	364.0	1.03	0.034	0.00	0.0050	0.0001	0.15	34	3	32	1	180	130	32.3	0.7	5.1	
615-11-78	265.8	2.42	0.810	0.01	0.0982	0.0012	0.30	602	8	604	7	596	41	603.6	6.8	0.2	
615-11-79	126.9	1.69	1.777	0.04	0.1756	0.0021	0.21	1037	15	1042	11	1029	48	1029.0	48.0	1.3	
615-11-80	87.3	0.56	4.147	0.06	0.2930	0.0030	0.47	1664	12	1656	15	1670	28	1670.0	28.0	0.8	
615-11-81	335.0	1.35	1.650	0.05	0.1561	0.0049	0.56	988	18	935	27	1124	55	1124.0	55.0	16.8	
615-11-82	281.0	2.40	2.891	0.06	0.2235	0.0041	0.62	1380	16	1299	22	1518	36	1518.0	36.0	14.4	
615-11-83	270.3	1.11	16.190	0.19	0.4826	0.0053	0.57	2888	11	2538	23	3134	17	3134.0	17.0	19.0	
615-11-84	117.8	1.06	2.242	0.04	0.2026	0.0022	0.43	1192	11	1190	12	1216	31	1216.0	31.0	2.1	
615-11-86	463.0	0.47	11.520	0.11	0.4665	0.0063	0.72	2566	9	2467	28	2647	16	2647.0	16.0	6.8	
615-11-87	1114.0	1.98	12.440	0.12	0.5040	0.0045	0.70	2637	9	2632	19	2634	12	2634.0	12.0	0.1	
615-11-88	526.0	3.47	1.815	0.03	0.1709	0.0020	0.42	1052	10	1017	11	1131	31	1131.0	31.0	10.1	

	[U] ppm	U/Th	$^{207}Pb/^{235}U$	2σ error	$^{206}Pb/^{238}U$	2σ error	RHO	$^{207}Pb/^{235}U$ Age Ma	2σ error	$^{206}Pb/^{238}U$ Age (Ma)	2σ error	$^{207}Pb/^{206}Pb$ Age (Ma)	2σ error	Best age (Ma)	2σ error	% Disc*	Rim or core
615-11-89	1254.0	2.73	1.738	0.02	0.1710	0.0016	0.68	1022	6	1018	9	1026	16	1026.0	16.0	0.8	
615-11-90	38.1	2.24	2.990	0.09	0.2358	0.0048	0.60	1405	22	1366	25	1468	47	1468.0	47.0	6.9	
615-11-91	264.0	1.38	1.813	0.02	0.1755	0.0015	0.22	1049	9	1042	8	1056	26	1056.0	26.0	1.3	
615-11-92	14.4	0.50	1.810	0.12	0.1726	0.0081	0.79	1020	42	1021	44	1069	93	1069.0	93.0	4.5	
615-11-93	341.0	1.42	4.110	0.07	0.2863	0.0047	0.55	1656	14	1622	23	1696	25	1696.0	25.0	4.4	
615-11-94	180.0	1.60	16.210	0.19	0.5596	0.0066	0.63	2888	11	2864	27	2903	16	2903.0	16.0	1.3	
615-11-95	59.4	0.61	13.640	0.20	0.5358	0.0073	0.80	2726	13	2763	31	2684	16	2684.0	16.0	2.9	
615-11-96	182.0	1.42	1.796	0.03	0.1700	0.0018	0.37	1046	10	1012	10	1107	30	1107.0	30.0	8.6	
615-11-97	92.8	0.59	2.465	0.06	0.2101	0.0037	0.47	1260	17	1229	20	1303	43	1303.0	43.0	5.7	
615-11-98	167.3	1.37	2.218	0.04	0.1975	0.0023	0.32	1187	12	1162	13	1257	35	1257.0	35.0	7.6	
615-11-99	412.0	8.86	0.609	0.04	0.0739	0.0018	0.06	482	24	459	11	570	150	459.0	11.0	4.8	Rim
615-11-99	874.0	7.29	1.139	0.03	0.1163	0.0013	0.58	774	13	709	7	928	45	708.9	7.3	8.4	Core
615-11-99	120.0	0.86	1.598	0.04	0.1550	0.0021	0.44	970	17	931	12	1071	53	1071.0	53.0	13.1	Core
615-11-100	460.0	2.03	4.410	0.05	0.2946	0.0029	0.53	1715	9	1664	15	1776	19	1776.0	19.0	6.3	
615-11-101	260.3	0.40	0.058	0.01	0.0057	0.0002	0.06	55	6	37	1	880	240			33.8	Rim
615-11-101	50.1	0.84	3.150	0.17	0.0317	0.0019	0.60	1444	39	201	12	4805	87			86.1	Core
615-11-102	344.1	2.10	2.517	0.05	0.1653	0.0044	0.73	1282	16	986	24	1808	31			45.5	
615-11-103	1132.0	0.86	0.079	0.00	0.0117	0.0001	0.15	77	2	75	1	112	53	75.2	0.8	2.3	
615-11-104	118.8	1.46	4.673	0.06	0.3160	0.0034	0.46	1761	10	1770	17	1757	23	1757.0	23.0	0.7	
615-11-105	213.0	1.42	3.214	0.03	0.2544	0.0020	0.49	1460	8	1462	10	1458	17	1458.0	17.0	0.3	
615-11-106	127.0	0.97	3.144	0.05	0.2511	0.0026	0.48	1445	11	1445	13	1424	24	1424.0	24.0	1.5	
615-11-107	329.0	1.37	2.089	0.02	0.1928	0.0015	0.45	1146	8	1137	8	1142	20	1142.0	20.0	0.4	
615-11-108	428.0	2.24	1.542	0.03	0.1396	0.0023	0.58	946	12	842	13	1180	35			11.0	
615-11-109	323.0	1.75	3.038	0.06	0.2393	0.0036	0.42	1418	14	1383	19	1466	35	1466.0	35.0	5.7	
615-11-110	259.0	1.25	3.528	0.06	0.2847	0.0040	0.46	1533	13	1615	20	1433	30	1433.0	30.0	12.7	
615-11-111	178.7	1.30	1.840	0.03	0.1706	0.0016	0.18	1060	10	1015	9	1140	29	1140.0	29.0	11.0	
615-11-112	123.6	0.64	11.600	0.21	0.4637	0.0099	0.64	2571	17	2458	43	2668	28	2668.0	28.0	7.9	
615-11-113	99.4	0.89	1.981	0.05	0.1896	0.0030	0.31	1108	16	1119	16	1087	49	1087.0	49.0	2.9	
615-11-114	232.7	1.40	0.595	0.02	0.0736	0.0011	0.39	475	11	458	7	530	61	458.0	6.8	3.6	
615-11-115	204.8	0.94	12.810	0.13	0.5071	0.0049	0.66	2667	9	2643	21	2684	13	2684.0	13.0	1.5	
615-11-116	151.5	0.79	4.554	0.06	0.3046	0.0031	0.55	1742	10	1714	15	1769	20	1769.0	20.0	3.1	
615-11-117	851.0	4.62	1.907	0.04	0.1779	0.0026	0.35	1083	13	1055	14	1141	41	1141.0	41.0	7.5	Rim
615-11-117	254.0	1.87	2.444	0.04	0.2120	0.0030	0.31	1255	12	1240	16	1282	36	1282.0	36.0	3.3	Core
615-11-118	180.1	0.93	0.030	0.00	0.0043	0.0002	0.03	30	3	28	1	70	200	27.6	1.3	6.8	
615-11-119	53.9	0.80	4.412	0.09	0.2913	0.0050	0.53	1713	17	1647	25	1778	32	1778.0	32.0	7.4	

Detrital zircon mixing models

Supplementary Figure Supp. 1 shows mixing model results — the relative fractional contribution from each parent component (tributary samples), to each mixed daughter composite (core samples) from the deep-sea fan — highlighted in Table 1. Mixing model results quantify a significant difference between daughter sample composites that can be seen qualitatively in KDEs (Fig. S1), and in the MDS map (Fig. S2). Specifically, age modes in samples measured across H20 indicate a change in sediment source area or sediment loads from tributaries in the Mississippi system between ca. 10 – 70 ka.

For all samples above H20, the Missouri River component supplies between 57 – 87% of sediment. In most samples above H20, the Upper Mississippi parent component supplies the next greatest proportion of sediment, or between 10 – 26%, except where the Red River contributes 20% to sample 615-7. The Red River contributes between 0 – 20% in other samples above H20, and the Arkansas component has reliably low contributions between 0 – 5%. The Ohio River component contributed only 1% of the sediment found in each sample above H20. These results are consistent with previously published mixing fractions from late Wisconsin age sediment cores (26).

Unmixing DZ samples below H20 results in markedly different contributions of parent components. Most striking is a large decrease in contributions from the Missouri River component (only 15 – 36%). Remaining contributions to samples below H20 are well distributed between the Upper Mississippi, Ohio, and Red River. In all but one case, the Arkansas is absent, except where it contributes 17% to sample 615-11.

We amalgamated DZ ages from samples above H20 (n = 405 grains), and below H20 (n = 426 grains), then applied the same unmixing technique to each composite (Table 1). The results from amalgamated samples above and below H20 are broadly consistent with those from each individual sample, and show a trend of low contributions from the Missouri component between MIS-4 – MIS-3, which increases significantly across H20 during MIS-2. Above H20, models suggest decreasing contributions from the Ohio, Upper Mississippi, and Red River components during the LGM. Results of unmixing late Holocene sediment mixtures form the fluvio-deltaic Mississippi support a more distributed mixture that is strikingly similar to actual measured sediment loads (26).

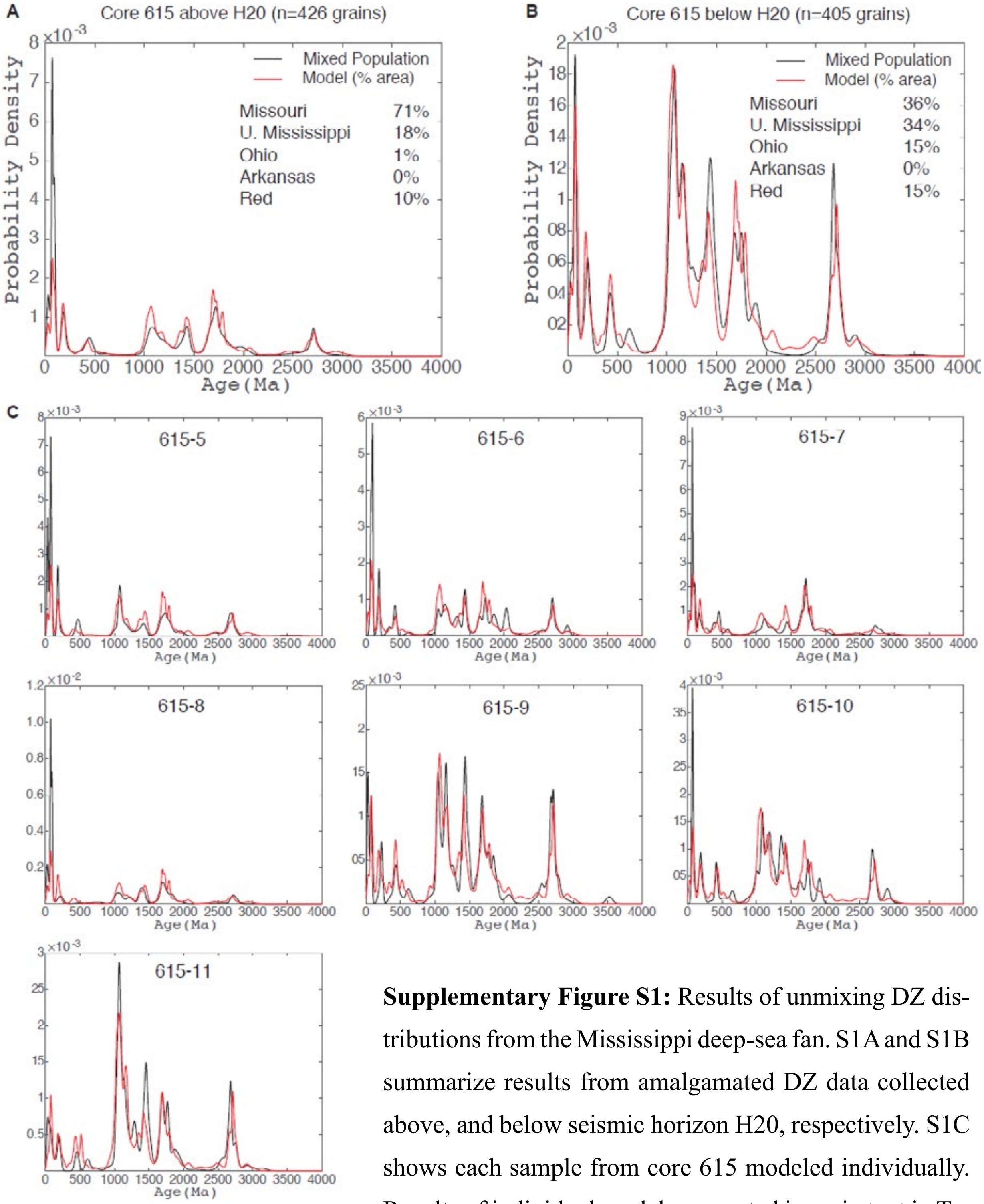

Supplementary Figure S1: Results of unmixing DZ distributions from the Mississippi deep-sea fan. S1A and S1B summarize results from amalgamated DZ data collected above, and below seismic horizon H20, respectively. S1C shows each sample from core 615 modeled individually. Results of individual models presented in main text in Table X, and graphically in Supplementary Figure S2.

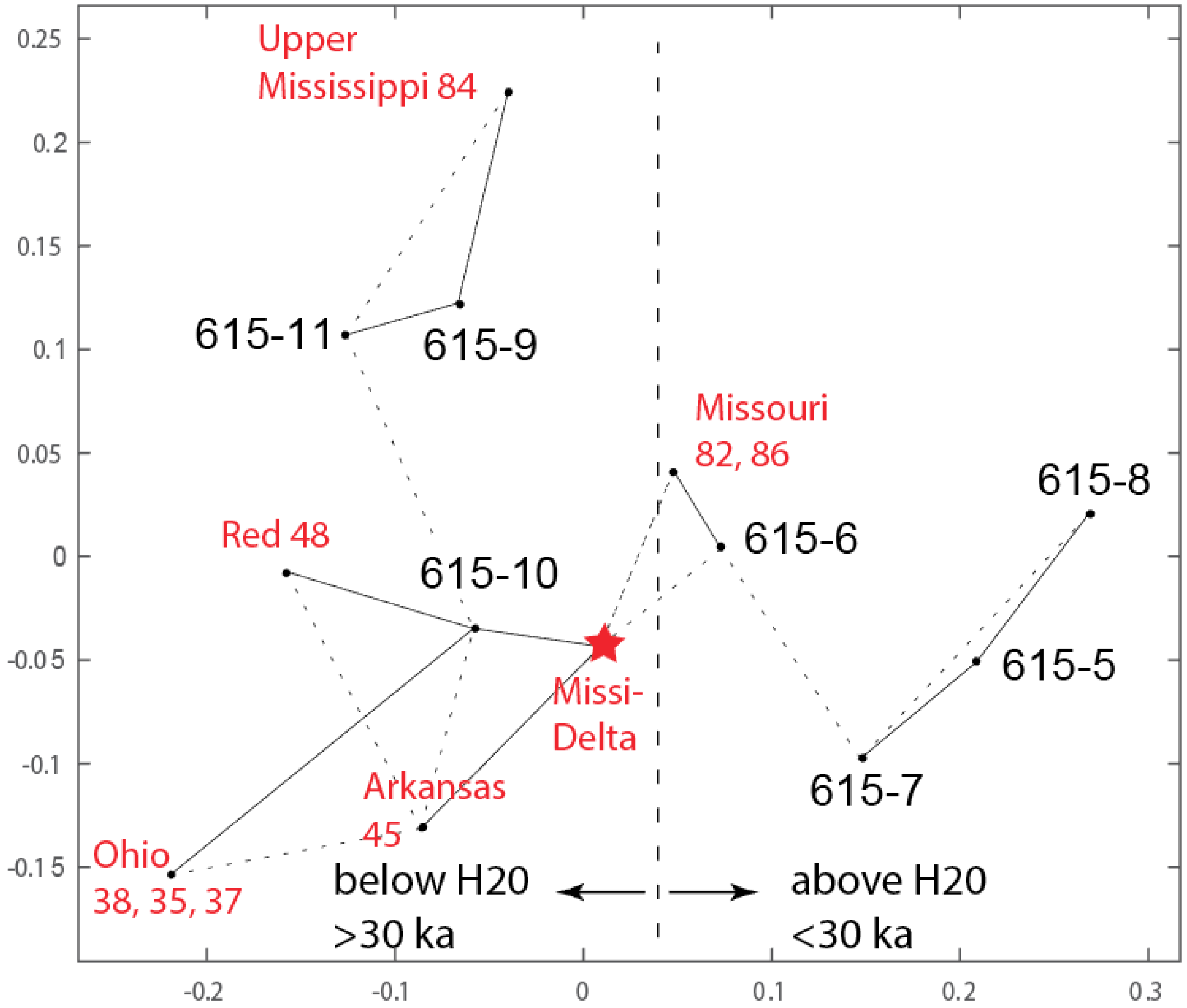

Supplementary Figure S2: Dissimilarity between samples can easily be quantified using a multidimentional scaling map (MDS) (http://www.ucl.ac.uk/~ucfbpve/provenance/). The tools use an objective statistical measure of dissimilarity related to the maximum difference in the cumulative distribution functions (CDF) between samples. MDS maps provide the best tool to visualize dissimilarity between multiple samples (this is a non-dimensional graph). We obtained a remarkable map of samples above and below the Seismic Horizon H20 and their affinities with potential sources areas (represented by individual river signatures).

Results of unmixing detrital zircon age distributions from the deep-sea Mississippi Fan

Timescale	Late Holocene	MIS-2				~10 – 30 ka	MIS-3/4		MIS-4	~30 – 70 ka
DZ sample ID (right)	*Mississippi River	Core 615-5	Core 615-6	Core 615-7	Core 615-8	all samples above H20	Core 615-9	Core 615-10	Core 615-11	all samples below H20
Parent Tributary (column below)	fraction of total contribution									
**Missouri**	0.59	0.70	0.57	0.75	0.87	**0.71**	0.23	0.34	0.15	**0.36**
**Upper Mississippi**	0.14	0.26	0.24	0.00	0.10	**0.18**	0.40	0.26	0.39	**0.34**
**Ohio**	0.13	0.01	0.01	0.00	0.01	**0.01**	0.05	0.18	0.19	**0.15**
**Arkansas**	0.00	0.03	0.00	0.05	0.00	**0.00**	0.00	0.00	0.17	**0.00**
**Red**	0.14	0.00	0.18	0.20	0.02	**0.10**	0.31	0.22	0.10	**0.15**

*Fluvio-deltaic estimates from Mason et al. (2017)

DSDP Site 615 (U-Th)/He zircon isotopic data

Sample	Age (Ma)	err. (Ma)	U (ppm)	Th (ppm)	^{147}Sm (ppm)	[U]e	Th/U	He (nmol/g)	mass (ug)	Ft	ESR	U-Pb Age (Ma)	err. (Ma)
z615-8-1	210.0	16.80	22.2	10.5	2.2	24.6	0.47	20.7	3.20	0.73	42.96	1043	42
z615-8-2	395.7	31.66	229.0	43.1	0.6	238.9	0.19	423.0	7.96	0.80	59.64	1021	10
z615-8-3	87.9	19.03	216.1	101.7	0.8	239.5	0.47	82.8	4.17	0.74	45.56	96.6	1.6
z615-8-5	266.1	21.29	109.5	73.6	0.8	126.4	0.67	149.4	10.09	0.81	61.93	1728	12
z615-8-9	1265.1	101.21	20.3	11.0	0.5	22.9	0.54	139.8	7.67	0.79	57.93	1372	35
z615-8-11	927.7	74.21	56.5	32.7	2.4	64.1	0.58	275.5	7.59	0.79	57.83	2577	10
z615-8-12	79.0	6.32	280.5	47.1	0.5	291.3	0.17	93.9	4.06	0.75	46.36	1372	15
z615-8-14	65.7	12.46	159.2	59.6	0.7	173.0	0.37	47.8	8.24	0.80	59.41	74.5	1.1
z615-8-28	182.1	30.57	44.6	26.8	0.7	50.7	0.60	29.3	3.78	0.74	44.53	233.7	3.3
z615-8-29	919.2	73.54	26.8	9.5	0.3	29.0	0.35	128.3	11.77	0.82	65.93	1944	13
z615-8-32	65.6	13.24	16.5	9.5	0.1	18.7	0.57	3.9	12.81	0.82	67.41	72.1	3.1
z615-8-35	36.8	7.74	142.0	29.9	0.4	148.9	0.21	60.7	5.71	0.78	51.55	1384	14
z615-8-45	212.3	56.98	51.8	11.4	0.6	54.4	0.22	64.1	6.52	0.79	54.20	483.8	6.4
z615-8-50	957.5	116.60	62.2	28.5	1.0	68.8	0.46	312.3	10.06	0.81	63.14	1107	38
z615-8-53	282.2	22.58	253.3	79.4	3.1	271.5	0.31	273.5	1.46	0.65	31.74	781.8	7.1
z615-8-65	92.8	13.83	62.8	26.8	0.2	69.0	0.43	28.8	5.89	0.75	46.52	110.1	1.9
z615-8-74	84.2	16.34	30.2	22.2	0.3	35.3	0.73	13.8	8.83	0.80	61.10	163.1	3.3
z615-8-78	31.7	2.53	23.1	12.0	0.7	25.9	0.52	3.3	6.25	0.76	47.79	11.8	1.1
z615-8-94	2008.3	160.66	16.7	7.2	0.3	18.4	0.43	309.1	17.67	0.84	76.73	2704	15
z615-8-109	170.1	13.61	65.3	39.5	0.5	74.4	0.60	50.0	3.04	0.72	42.02	675.5	8.3
z615-10b-27	722.2	4.14	24.0	26.9	1.5	30.2	1.12	92.8	4.13	0.75	47.29	71	1.2
z615-10-b-28	134.4	0.63	147.1	87.9	18.2	167.4	0.60	86.5	2.41	0.71	39.17	2733	20
z615-10-b-39	1171.5	5.21	57.5	40.1	7.4	66.8	0.70	401.5	20.17	0.85	79.89	1079	47
z615-10-b-40	1011.2	4.49	61.9	36.5	3.0	70.4	0.59	356.2	17.22	0.84	77.34	1153	58
z615-10-b-47	36.9	0.16	100.2	62.7	1.4	114.6	0.63	18.1	7.51	0.79	56.05	79.4	2
z615-10-b-7	83.4	0.38	143.7	61.6	1.5	157.9	0.43	54.1	4.33	0.76	47.94	81	1.6
z615-10-b-9	718.2	3.69	15.2	10.2	2.4	17.6	0.67	59.4	11.55	0.82	67.66	1130	120
z615-10-b-14	667.0	3.29	166.0	31.9	17.8	173.5	0.19	489.1	3.46	0.74	44.64	114	39

Sample	Age (Ma)	err. (Ma)	U (ppm)	Th (ppm)	^{147}Sm (ppm)	[U]e	Th/U	He (nmol/g)	mass (ug)	Ft	ESR	U-Pb Age (Ma)	err. (Ma)
z615-10-b-20	122.9	0.62	53.6	27.2	1.5	59.9	0.51	30.0	3.76	0.75	46.34	1081	29
z615-10-b-17	913.6	4.36	77.7	28.2	10.8	84.2	0.36	367.7	9.88	0.82	64.20	1644	28
z615-10-b-50	455.5	2.43	46.8	18.7	2.3	51.1	0.40	97.8	4.03	0.75	47.16	1809	42
z615-10-b-49	564.5	2.57	118.5	55.4	3.5	131.3	0.47	332.1	7.62	0.79	57.62	1454	26
z615-10-115	1096.5	5.10	52.8	48.5	3.7	64.0	0.92	306.3	3.98	0.74	45.94	1331	30
z615-10-30	184.6	0.92	115.2	48.4	16.9	126.4	0.42	96.8	4.98	0.76	48.31	960	100
z615-10-15	771.9	3.58	150.0	69.6	14.5	166.1	0.46	546.4	3.87	0.74	45.42	1436	51
z615-10-b-44	931.3	74.51	121.5	47.2	4.4	132.3	0.39	540.7	4.54	0.75	47.26	2687	15
z615-10-b-46	62.8	5.03	201.0	115.2	14.5	227.6	0.57	59.0	4.59	0.76	49.07	2940	49
z615-11-38	1178.5	5.46	42.2	18.5	2.7	46.4	0.44	275.9	13.46	0.83	71.20	1412	26
z615-11-7	962.6	4.40	29.5	17.7	4.5	33.6	0.60	167.9	38.78	0.88	98.33	1489	23
z615-11-8	1298.7	5.65	44.5	32.7	1.8	52.1	0.73	348.2	16.17	0.84	74.98	2682	16
z615-11-b-6	800.7	3.67	48.7	29.3	2.0	55.4	0.60	202.5	7.42	0.79	57.14	1072	49
z615-11-b-11	367.7	1.63	142.4	90.1	2.8	163.2	0.63	254.6	4.77	0.77	50.39	1740	68
z615-11-b-46	83.6	0.41	542.1	53.3	1.7	554.4	0.10	176.6	2.08	0.70	37.56	1733	26
z615-11-b-10	447.1	2.05	101.9	47.2	3.3	112.7	0.46	217.8	5.92	0.77	52.02	1081	43
z615-11-b-36	338.0	2.52	25.8	11.7	2.3	28.5	0.45	38.9	3.01	0.73	43.23	1647	61
z615-11-b-37	26.1	0.12	57.9	55.5	2.9	70.7	0.96	7.2	3.11	0.72	41.65	30.5	1.9
z615-11-b-31	2468.8	95.18	1.2	12.1	1.1	4.0	9.76	44.1	3.13	0.72	43.41	2951	29
z615-11-b-34	718.7	62.83	2.7	0.7	-0.3	2.9	0.26	8.2	2.12	0.70	37.83	1126	71
z615-11-b-1	77.8	0.35	116.5	61.6	1.1	130.7	0.53	44.4	9.93	0.80	60.98	73.3	2.5
z615-11-b-13	1000.3	4.39	68.0	58.6	5.4	81.5	0.86	366.4	5.75	0.77	51.51	1044	62
z615-11-b-42	298.3	1.87	26.2	17.5	0.4	30.3	0.67	36.7	3.70	0.74	44.43	2645	33
z615-9b-32	46.2	3.70	215.0	159.0	2.6	251.6	0.74	50.1	7.56	0.79	58.05	49.1	1.4
z615-9b-39	30.1	2.41	205.6	78.9	1.8	223.7	0.38	29.7	11.30	0.81	63.80	27.07	0.73
z615-9b-8	441.3	35.31	84.0	28.4	3.2	90.5	0.34	181.1	9.30	0.81	62.63	1027	73
z615-9b-3	719.3	57.55	124.9	11.8	2.0	127.6	0.09	466.7	35.57	0.88	98.46	1047	66
z615-9b-6	465.2	37.21	346.3	221.8	15.2	397.5	0.64	848.5	10.98	0.82	66.57	1390	34
z615-9b-37	550.9	44.07	84.1	54.3	1.7	96.6	0.65	241.1	8.76	0.80	61.23	1628	53

Sample	Age (Ma)	err. (Ma)	U (ppm)	Th (ppm)	^{147}Sm (ppm)	[U]e	Th/U	He (nmol/g)	mass (ug)	Ft	ESR	U-Pb Age (Ma)	err. (Ma)
z615-9b-42	347.9	27.83	105.1	29.1	7.9	111.9	0.28	180.1	13.75	0.83	71.76	1488	40
z615-9b-7	157.9	12.63	58.5	40.4	3.2	67.8	0.69	49.4	17.65	0.84	77.68	1674	31
z615-9b-12	54.9	4.40	132.2	99.4	13.5	155.2	0.75	37.9	11.26	0.82	66.41	1838	38
z615-9b-47	648.3	51.87	161.8	66.7	46.6	177.4	0.41	557.1	19.00	0.85	79.01	1667	35
z615-9b-31	258.6	20.69	317.1	180.0	20.7	358.6	0.57	404.4	7.30	0.79	57.18	2757	29
z615-9b-43	407.2	32.58	167.1	59.1	6.1	180.7	0.35	313.1	4.53	0.77	49.53	2724	50
z615-9-32	1049.0	83.92	33.4	22.9	9.5	38.7	0.68	202.3	15.97	0.84	74.39	2638	20
z615-9-15	441.4	35.31	51.4	13.3	1.1	54.4	0.26	108.0	8.44	0.80	60.14	2804	34
z615-9-68	462.2	36.97	52.9	29.2	2.0	59.6	0.55	130.8	18.70	0.85	78.72	419.4	7.1
z615-9-79	253.0	20.24	157.0	45.0	1.8	167.3	0.29	193.4	13.16	0.83	69.97	368.9	7.4
z614-1-67	60.3	4.82	202.9	79.5	0.5	221.2	0.39	53.9	4.40	0.75	45.56	57.8	1.7
z614-1-41	60.9	4.87	292.4	104.8	0.9	316.5	0.36	80.4	5.49	0.77	50.49	62.39	0.91
z614-1-20	63.2	5.06	201.4	34.7	0.1	209.4	0.17	51.7	2.59	0.72	40.64	64.5	1.6
z614-1-77	61.8	4.95	211.1	91.5	0.7	232.2	0.43	53.0	1.92	0.68	35.59	84.5	2.4
z614-1-75	84.4	6.75	214.2	82.8	0.4	233.2	0.39	85.0	8.04	0.79	57.58	93.4	1.4
z614-1-61	136.0	10.88	318.8	153.9	0.9	354.2	0.48	190.4	4.01	0.73	42.09	97.2	1.4
z614-1-46	505.5	40.44	48.1	21.6	0.8	53.1	0.45	107.4	2.58	0.72	40.50	1022	18
z614-1-80	267.7	21.41	82.7	44.6	1.6	93.0	0.54	88.8	1.47	0.65	32.11	1076	36
z614-1-55	429.3	34.35	193.7	50.5	2.0	205.3	0.26	344.4	2.29	0.70	38.04	1168	24
z614-1-64	626.9	50.15	116.6	50.4	2.6	128.2	0.43	355.8	6.04	0.78	53.72	1428	26
z614-1-115	439.3	35.14	64.7	43.4	0.9	74.6	0.67	129.6	2.95	0.71	40.07	1466	23
z614-1-53	114.4	9.15	45.8	42.0	1.1	55.5	0.92	24.9	2.95	0.72	41.71	1672	35
z614-1-59	76.1	6.09	133.8	61.9	0.4	148.0	0.46	46.4	4.46	0.76	48.36	1725	14
z614-1-29	221.3	17.70	118.4	65.4	0.5	133.5	0.55	123.8	4.92	0.76	49.95	1741	19
z614-1-72	908.8	72.70	64.9	92.1	2.2	86.1	1.42	356.7	7.55	0.79	57.15	2655	15
z614-1-23	304.2	24.34	108.5	56.9	1.1	121.7	0.52	157.4	5.61	0.77	51.28	2713	10
z614-1-1	696.2	55.69	73.0	53.0	2.1	85.2	0.73	243.6	3.11	0.72	42.24	2723	12
z621-9-12	339.2	27.14	8.9	2.8	0.1	9.6	0.32	14.7	11.66	0.82	64.55	1113	71
z621-9-19	67.1	13.37	138.5	44.5	0.4	148.7	0.32	31.1	4.83	0.75	45.24	67	1.8

Sample	Age (Ma)	err. (Ma)	U (ppm)	Th (ppm)	^{147}Sm (ppm)	[U]e	Th/U	He (nmol/g)	mass (ug)	Ft	ESR	U-Pb Age (Ma)	err. (Ma)
z621-9-22	724.5	57.96	332.6	156.7	12.5	368.8	0.47	1260.4	13.07	0.82	67.06	1782	22
z621-9-34	9.3	4.74	13.7	7.2	0.3	15.3	0.53	1.0	16.58	0.82	64.83	9.96	0.8
z621-9-40	449.2	35.93	179.8	58.1	0.8	193.1	0.32	377.8	6.41	0.78	53.17	1075	19
z621-9-45	83.6	144.29	28.9	17.5	0.5	32.9	0.61	316.1	8.91	0.80	58.78	995	68
z621-9-46	2147.3	171.78	17.2	10.9	0.1	19.7	0.63	319.2	14.88	0.82	67.58	2689	16
z621-9-48	343.9	27.51	175.6	29.7	2.9	182.4	0.17	271.3	6.54	0.78	53.00	1765	39
z621-9-50	113.2	9.06	173.1	20.4	0.4	177.8	0.12	85.6	5.84	0.78	53.03	227.5	3.6
z621-9-53	53.9	4.31	157.5	97.6	1.1	180.0	0.62	38.4	3.29	0.73	43.11	1716	23
z621-9-55	280.5	25.08	113.5	27.6	0.2	119.9	0.24	160.6	5.48	0.77	51.30	270.8	3.5
z621-9-59	93.3	15.47	343.4	105.6	0.9	367.7	0.31	157.6	8.41	0.79	56.05	91	1.6
z621-9-61	36.8	6.94	67.1	22.0	0.3	72.2	0.33	8.0	16.38	0.82	67.28	36.7	1.3
z621-9-64	695.3	55.62	621.0	179.3	11.6	662.3	0.29	1852.3	2.84	0.71	39.05	2712	21
z621-9-66	2230.5	178.44	31.9	15.5	0.9	35.4	0.48	500.0	6.05	0.78	52.84	2703	17
z621-9-71	102.3	8.19	31.8	9.3	0.1	34.0	0.29	14.8	6.37	0.78	54.24	156.4	7.7
z621-9-75	125.4	26.03	152.6	68.4	0.2	168.3	0.45	75.8	6.09	0.78	53.14	186.5	3
z621-9-77	83.3	6.66	391.6	161.2	0.9	428.7	0.41	153.9	9.53	0.79	57.24	1119	17
z621-9-78	63.4	21.07	71.8	34.6	0.3	79.7	0.48	25.6	4.40	0.74	45.00	71	2.1

DSDP Site 615 sand framework analysis

Sample[1]	Depth (mbsf)	$Quartz_m$	$Quartz_p$	$Feldspar_{plg}$	$Feldspar_{ksp}$	$Lithic_{volc}$	$Lithic_{plut}$	$Lithic_{meta}$	$Lithic_{sed\text{-}Clastic}$	$Lithic_{sed\text{-}Carb}$	Mica	Heavy min	Opaque	Hornblende	Pyroxene	$Organic_{notCarb}$
615-4-4sz	25.17	199	21	45	25	24	0	6	8	18	3	4	7	3	1	3
615-7-4sz	53.90	158	18	35	17	17	0	2	15	15	1	1	4	2	1	1
615-10-6sz	85.40	145	9	48	29	24	4	5	9	23	0	2	9	1	0	2
615-16-1sz	135.01	175	18	32	34	14	6	1	13	11	1	4	4	2	2	1
615-29-5sz	264.20	195	6	30	29	7	2	2	8	11	6	4	4	3	1	3
615-36-1s	333.75	175	6	47	26	24	2	2	0	16	6	2	7	0	0	4
615-43-5sz	415.68	160	6	42	27	12	2	2	8	24	3	2	4	3	1	1
615-47-1sz	457.02	154	9	61	36	7	0	0	4	29	3	2	4	2	1	0

[1]samples are were collected from the IODP Gulf Coast Repository in College Station, TX USA. Names defined by Site#-Core#-Sec#; s/sz = sand

DSDP Site 615 mud geochemistry

Method		GO_XRF76V													
DL		-10	0.01	0.01	0.01	0.01	0.01	0.01	0.01	0.01	0.01	0.01	0.01	0.01	0
Sample[1]	Depth (mbsf)	LOI %	SiO_2 %	Al_2O_3 %	Fe_2O_3 %	MgO %	CaO %	K_2O %	Na_2O %	TiO_2 %	MnO %	P_2O_5 %	Cr_2O_3 %	V_2O_5 %	Sum %
615-1-1m	0.5	16	49.5	15.4	6.04	2.99	3.62	2.81	2.89	0.62	0.12	0.16	0.02	0.03	100.2
615-3-1m	13.07	13.6	52.8	15.5	6.24	3.12	2.93	2.91	2.33	0.66	0.12	0.18	0.02	0.03	100.4
615-4-1m	20.75	11.9	56.3	13.8	5.39	2.92	3.38	2.48	2.48	0.59	0.13	0.16	0.01	0.02	99.6
615-6-2m	41.63	14.5	52.4	14.5	5.91	3.03	4.35	2.47	1.96	0.6	0.13	0.16	0.01	0.03	100.1
615-8-1m	58.15	12.5	58.9	12.1	4.26	3	5.04	2.28	1.8	0.53	0.1	0.15	0.01	0.02	100.6
615-9-3m	70.8	11.7	55.1	15.7	5.81	2.87	3.36	2.64	1.83	0.62	0.1	0.15	0.01	0.03	99.9
615-11-3m	90.52	10.8	59.5	12.3	4.61	2.94	4.46	2.45	1.77	0.6	0.11	0.16	0.01	0.03	99.8
615-18-1m	153.96	12.3	57.4	13.9	5.05	2.76	3.73	2.55	1.73	0.58	0.11	0.16	0.01	0.02	100.3
615-19-1m	162.7	11.6	54.5	15.6	6.39	2.82	3.55	2.69	1.61	0.62	0.11	0.16	0.01	0.03	99.7
615-22-2m	193.26	12.9	53.9	16.3	6.15	2.79	3.14	2.69	1.52	0.62	0.09	0.16	0.01	0.03	100.3
615-23-1m	200.28	11.3	59.9	13.1	4.59	2.5	3.66	2.49	1.6	0.54	0.1	0.14	<0.01	0.02	100
615-27-1m	238.42	12	54.2	14.4	5.94	3.11	4.62	2.83	1.58	0.63	0.14	0.14	0.01	0.03	99.6
615-29-1m	257.47	11.6	56.9	13.2	5.01	3.04	4.93	2.72	1.55	0.59	0.12	0.14	<0.01	0.02	99.8
615-32-1m	286.53	13.7	52.5	13.9	5.41	3.29	5.77	2.83	1.54	0.62	0.13	0.15	0.02	0.02	99.8
615-33-1m	305.38	13.1	55.9	11.5	4.26	3.58	6.71	2.54	1.42	0.53	0.11	0.13	0.01	0.02	99.9
615-34-3m	317.24	12.5	55.5	13.7	5.24	2.81	5.21	2.71	1.47	0.59	0.17	0.16	0.02	0.03	100.1
615-36-1m	333.29	12.3	56	13.2	4.75	2.94	5.68	2.75	1.44	0.6	0.2	0.14	0.01	0.03	100.1
615-38-1m	352.52	13.9	51.2	15.4	6.64	2.95	4.77	2.86	1.56	0.62	0.2	0.17	0.02	0.03	100.3
615-40-1m	371.71	13.9	51.3	14.5	5.66	3.64	5.59	3.15	1.38	0.64	0.09	0.14	0.02	0.02	100
615-44-1m	418.71	12.9	56.7	12.5	4.22	3.37	5.33	2.7	1.44	0.57	0.09	0.13	<0.01	0.02	99.9
615-47-1m	457.25	13.7	51	14.5	6.13	3.38	5.45	2.99	1.66	0.63	0.1	0.15	0.01	0.03	99.7
REP-615-40-1m															
REP-615-47-1m															

DSDP Site 615 mud geochemistry

Method		GE_ICM90A													
DL		0.01	10	5	0.1	10	10	0.01	0.1	10	0.01	10	5	0.01	5
Sample[1]	Depth (mbsf)	Al	Ba	Be	Ca	Cr	Cu	Fe	K	Li	Mg	Mn	Ni	P	Sc
		%	ppm	ppm	%	ppm	ppm	%	%	ppm	%	ppm	ppm	%	ppm
615-1-1m	0.5	8.69	445	<5	2.7	87	46	4.38	2.5	53	1.78	889	41	0.07	14
615-3-1m	13.07	8.84	512	<5	2	97	38	4.56	2.5	49	1.91	885	41	0.08	15
615-4-1m	20.75	7.45	522	<5	2.3	86	30	3.94	2.1	44	1.71	932	39	0.07	11
615-6-2m	41.63	8.22	504	<5	3.1	87	32	4.28	2.2	49	1.87	897	40	0.07	13
615-8-1m	58.15	6.65	546	<5	3.4	65	27	3.01	2	34	1.82	755	32	0.07	10
615-9-3m	70.8	8.59	530	<5	2.3	90	31	4.23	2.2	51	1.69	745	49	0.07	14
615-11-3m	90.52	6.89	601	<5	3.1	76	28	3.4	2.1	38	1.73	778	38	0.07	10
615-18-1m	153.96	7.84	550	<5	2.6	74	35	3.63	2.3	43	1.7	754	36	0.07	12
615-19-1m	162.7	8.65	492	<5	2.5	89	32	4.68	2.3	50	1.65	787	42	0.07	13
615-22-2m	193.26	9.26	472	<5	2.2	88	34	4.42	2.3	51	1.72	693	41	0.07	15
615-23-1m	200.28	7.47	551	<5	2.6	71	29	3.32	2.3	39	1.55	690	39	0.06	11
615-27-1m	238.42	7.85	528	<5	3.2	80	30	4.35	2.4	49	1.8	979	48	0.06	12
615-29-1m	257.47	7.35	512	<5	3.4	83	30	3.66	2.3	43	1.78	815	38	0.06	11
615-32-1m	286.53	7.99	452	<5	4.1	78	33	3.92	2.5	51	2.02	985	39	0.06	12
615-33-1m	305.38	6.35	510	<5	4.6	72	25	3.07	2.1	37	2.12	772	40	0.05	9
615-34-3m	317.24	7.73	505	<5	3.6	75	36	3.75	2.4	46	1.73	1214	44	0.07	12
615-36-1m	333.29	7.24	508	<5	3.9	81	32	3.5	2.3	45	1.76	1535	50	0.06	11
615-38-1m	352.52	8.51	450	<5	3.2	81	32	4.7	2.4	52	1.79	1444	45	0.07	14
615-40-1m	371.71	8.08	437	<5	3.9	80	28	4.06	2.8	52	2.19	681	48	0.06	14
615-44-1m	418.71	6.87	542	<5	3.7	73	32	3.1	2.2	41	1.99	650	42	0.05	11
615-47-1m	457.25	7.83	466	<5	3.8	83	35	4.47	2.5	50	1.97	759	40	0.06	13
REP-615-40-1m		8.19	433	<5	3.9	82	28	4.04	2.7	51	2.24	682	50	0.06	14
REP-615-47-1m		8.12	444	<5	3.8	82	37	4.53	2.5	52	1.97	764	41	0.06	13

DSDP Site 615 mud geochemistry

Method		GE_ICM90A												
DL		0.1	10	0.01	5	5	1	5	0.1	0.2	0.1	0.5	0.1	0.05	0.05
Sample[1]	Depth (mbsf)	Si	Sr	Ti	V	Zn	Ag	As	Bi	Cd	Ce	Co	Cs	Dy	Er
		%	ppm	%	ppm	ppm	ppm	ppm	ppm	ppm	ppm	ppm	ppm	ppm	ppm
615-1-1m	0.5	23.4	166	0.37	164	114	<1	14	0.3	0.3	84	16	7	4.94	2.54
615-3-1m	13.07	25.6	143	0.38	162	113	<1	12	0.3	0.2	84.8	16	6.4	5.29	2.89
615-4-1m	20.75	25.9	145	0.34	147	97	<1	7	0.3	0.3	70	13.9	6.1	4.52	2.64
615-6-2m	41.63	25.2	147	0.36	181	109	<1	14	0.4	0.3	65.8	14.9	6.8	4.41	2.3
615-8-1m	58.15	28.2	167	0.32	123	91	<1	11	0.3	0.4	57.6	10.8	4.9	4.21	2.28
615-9-3m	70.8	24.1	130	0.37	184	131	<1	15	0.3	<0.2	64.9	18.9	8	4.16	2.46
615-11-3m	90.52	26.9	152	0.36	124	94	<1	11	0.3	0.2	63.6	11.7	5.3	4.52	2.67
615-18-1m	153.96	26.9	153	0.35	147	102	<1	11	0.4	0.4	65.4	12.5	6.5	4.47	2.43
615-19-1m	162.7	25.5	138	0.37	177	116	<1	9	0.4	0.3	67.2	14.5	7.9	4.23	2.41
615-22-2m	193.26	25.8	135	0.37	186	111	<1	13	0.4	0.3	67.1	15.1	8.2	4.54	2.38
615-23-1m	200.28	28.4	161	0.32	136	99	<1	20	0.4	0.5	61.8	13	6.1	4.28	2.32
615-27-1m	238.42	24.6	140	0.37	150	107	<1	15	0.3	0.2	66.6	16.7	7.1	4.45	2.55
615-29-1m	257.47	26.4	156	0.34	131	89	<1	8	0.3	0.2	64.7	13.4	6.2	4.25	2.49
615-32-1m	286.53	24.4	167	0.37	149	89	<1	11	0.3	0.4	67.4	15.6	6.5	4.62	2.58
615-33-1m	305.38	25.7	164	0.32	107	75	<1	7	0.2	0.4	60.7	11.3	5.1	4.05	2.44
615-34-3m	317.24	26	181	0.35	153	96	<1	12	0.3	0.4	64.2	15.7	6.4	4.62	2.54
615-36-1m	333.29	24.9	174	0.35	146	126	<1	15	0.3	0.8	71.1	18.1	6.3	4.57	2.72
615-38-1m	352.52	24	157	0.35	173	108	<1	9	0.4	0.3	69.9	16.5	7.7	4.79	2.55
615-40-1m	371.71	24.7	145	0.38	146	90	<1	35	0.3	0.3	69.6	20.3	7	4.53	2.54
615-44-1m	418.71	26.2	153	0.35	111	88	<1	29	0.3	0.4	67	13.5	5.7	4.53	2.76
615-47-1m	457.25	23.9	147	0.38	140	100	<1	7	0.3	0.3	72.7	14.4	7.1	4.7	2.77
REP-615-40-1m		24.4	143	0.37	143	94	<1	35	0.3	0.3	68.7	17.8	7.1	4.71	2.44
REP-615-47-1m		23.8	149	0.38	137	110	<1	7	0.8	1.1	72.9	14.5	7.1	4.73	2.71

DSDP Site 615 mud geochemistry

Method		GE_ICM90A													
DL		0.05	1	0.05	1	1	0.05	0.2	0.1	0.05	2	1	0.1	5	0.05
Sample[1]	Depth (mbsf)	Eu	Ga	Gd	Ge	Hf	Ho	In	La	Lu	Mo	Nb	Nd	Pb	Pr
		ppm	ppm	ppm	ppm	ppm	ppm	ppm	ppm	ppm	ppm	ppm	ppm	ppm	ppm
615-1-1m	0.5	1.34	22	5.8	2	4	0.98	<0.2	43	0.41	8	13	36.8	23	9.91
615-3-1m	13.07	1.43	22	6.07	2	5	1.05	<0.2	43.1	0.45	<2	13	37.1	21	9.96
615-4-1m	20.75	1.21	18	4.91	2	5	0.94	<0.2	35.8	0.39	<2	12	30.6	21	8.19
615-6-2m	41.63	1.2	21	4.83	2	4	0.85	<0.2	33.5	0.35	2	13	29.4	20	7.77
615-8-1m	58.15	1.08	16	4.51	2	5	0.86	<0.2	29.5	0.35	<2	12	26.1	18	6.89
615-9-3m	70.8	1.13	20	4.67	2	4	0.85	<0.2	33.9	0.39	<2	11	28.9	21	7.73
615-11-3m	90.52	1.12	15	4.72	1	5	0.9	<0.2	32.3	0.41	<2	11	27.9	20	7.54
615-18-1m	153.96	1.11	19	4.97	2	5	0.9	<0.2	33.1	0.38	<2	13	29.2	25	7.83
615-19-1m	162.7	1.13	20	4.69	2	4	0.86	<0.2	34.9	0.41	2	13	30	20	7.98
615-22-2m	193.26	1.21	23	4.94	2	4	0.9	<0.2	34.8	0.38	2	14	30.2	32	8.07
615-23-1m	200.28	1.15	18	4.63	2	5	0.88	<0.2	31.5	0.38	3	12	27.6	19	7.48
615-27-1m	238.42	1.1	18	4.71	1	4	0.89	<0.2	34.3	0.37	2	12	29.3	21	7.82
615-29-1m	257.47	1.1	17	4.64	1	4	0.87	<0.2	33.3	0.37	<2	12	28.6	21	7.82
615-32-1m	286.53	1.2	20	4.94	2	5	0.92	<0.2	33.6	0.42	2	13	30.7	19	8.06
615-33-1m	305.38	1.03	14	4.31	1	4	0.83	<0.2	30.6	0.37	2	11	26.3	19	7.21
615-34-3m	317.24	1.19	19	4.97	2	5	0.92	<0.2	32.3	0.4	2	13	29.4	19	7.8
615-36-1m	333.29	1.22	17	4.87	1	4	0.93	<0.2	35.6	0.4	3	12	31.7	21	8.48
615-38-1m	352.52	1.22	22	5.12	2	4	0.91	<0.2	35.3	0.4	2	14	31.5	20	8.4
615-40-1m	371.71	1.2	20	4.95	2	4	0.93	<0.2	34.6	0.39	2	13	30.2	20	8.27
615-44-1m	418.71	1.25	16	4.96	1	4	0.93	<0.2	33.1	0.4	3	11	28.7	22	7.79
615-47-1m	457.25	1.26	19	5.04	2	4	0.94	<0.2	36.7	0.42	3	12	31.3	23	8.45
REP-615-40-1m		1.17	21	5.04	2	4	0.94	<0.2	34.4	0.39	3	13	31.1	21	8.04
REP-615-47-1m		1.25	19	5.11	2	4	0.95	<0.2	36.7	0.4	3	12	31.7	22	8.47

DSDP Site 615 mud geochemistry

Method		GE_ICM90A													
DL		0.2	0.1	0.1	1	0.5	0.05	0.1	0.5	0.05	0.05	1	0.5	0.1	0.5	0.001
Sample[1]	Depth (mbsf)	Rb	Sb	Sm	Sn	Ta	Tb	Th	Tl	Tm	U	W	Y	Yb	Zr	WtKg
		ppm	ppm	ppm	ppm	ppm	ppm	ppm	ppm	ppm	ppm	ppm	ppm	ppm	ppm	kg
615-1-1m	0.5	119	1	7.1	2	0.7	0.83	13.5	0.7	0.41	3.68	<1	24.3	2.6	124	0.024
615-3-1m	13.07	118	1	7	2	0.8	0.88	13.7	0.7	0.44	3.1	3	26.6	2.9	137	0.031
615-4-1m	20.75	101	0.8	6	4	0.8	0.73	12.3	0.6	0.39	3.37	1	24.6	2.5	186	0.032
615-6-2m	41.63	109	1	5.5	2	0.8	0.8	11.5	0.7	0.37	2.92	2	22.1	2.2	124	0.049
615-8-1m	58.15	91.4	1	5	2	0.7	0.7	9.8	0.6	0.36	2.76	2	22.4	2.3	172	0.028
615-9-3m	70.8	117	0.9	5.4	3	0.9	0.69	12.5	0.7	0.36	3.42	2	23.1	2.4	130	0.036
615-11-3m	90.52	92.7	0.8	5.4	3	0.8	0.74	11	0.6	0.39	3.12	2	24.6	2.5	187	0.037
615-18-1m	153.96	110	1	5.7	2	0.8	0.72	11.6	0.7	0.38	2.77	1	24.1	2.4	151	0.033
615-19-1m	162.7	117	1	5.7	3	0.8	0.71	12.9	0.7	0.37	3.22	3	23.2	2.3	128	0.036
615-22-2m	193.26	129	1.1	5.7	2	0.8	0.73	13.1	0.8	0.37	3.1	3	23.8	2.5	125	0.058
615-23-1m	200.28	107	1.1	5.4	2	0.7	0.74	10.9	0.7	0.38	2.67	2	23.3	2.3	151	0.041
615-27-1m	238.42	115	0.8	5.6	3	0.8	0.69	12	0.8	0.37	3.28	2	24	2.5	138	0.046
615-29-1m	257.47	107	0.8	5.5	3	0.7	0.71	11.2	0.7	0.37	3.03	2	23.8	2.4	154	0.044
615-32-1m	286.53	120	1	5.8	2	0.8	0.83	11.5	0.7	0.42	3.06	2	24.9	2.5	148	0.043
615-33-1m	305.38	95.1	0.9	5.3	5	0.7	0.7	10	0.6	0.35	3.06	2	22.3	2.2	164	0.046
615-34-3m	317.24	115	1.2	5.7	2	0.8	0.84	11.4	0.7	0.39	2.98	2	24.4	2.5	153	0.049
615-36-1m	333.29	110	1.2	5.8	2	0.8	0.77	11.7	0.8	0.42	4.31	2	25.2	2.6	162	0.031
615-38-1m	352.52	130	1	6	2	0.8	0.8	12.7	0.8	0.39	3.01	2	24.8	2.5	129	0.041
615-40-1m	371.71	130	1.1	5.9	2	0.8	0.79	12	0.7	0.38	3.15	2	24.3	2.5	137	0.043
615-44-1m	418.71	102	1.2	5.7	3	0.7	0.78	10.8	0.6	0.39	3.58	2	25.4	2.5	158	0.053
615-47-1m	457.25	122	0.9	6	5	0.8	0.78	12.5	0.8	0.4	3.41	2	25.3	2.5	129	0.034
REP-615-40-1m		130	1	6	2	0.8	0.76	12.1	0.7	0.39	3.1	2	24.4	2.4	140	
REP-615-47-1m		122	0.9	6.1	4	0.8	0.79	12.4	1.5	0.42	3.73	2	25.8	2.5	132	

DSDP Site 615 mud geochemistry

[1]Samples are were collected from the IODP Gulf Coast Repository in College Station, TX USA. Names defined by Site#-Core#-Sec#; m = mud

[2]Mud samples were dried and pulverized in Cr steel to 85% passing 75 µm, and geochemically analyzed using multi-element analysis by sodium fusion inductively coupled plasma atomic emission spectroscopy (ICP-AES), inductively-coupled plasma mass spectrometry (ICP-MS), and multi-element ore grade analysis by borate fusion x-ray diffraction (XRF) at SGS Mineral Analytical Services.

Calculations in text:

CIA = [Al2O3 / (Al2O3 + K2O + CaO* + Na2O)] * 100 where compositions are in molar proportions and where CaO* is non-carbonate

N = normalized to chondrite (Boynton, W., 1984. Geochemistry of the rare earth elements: Meteorite studies, in P. Henderson, ed., Rare Earth Element Geochemistry, Elsevier, p. 63-114)
